# Adding the AMBER 14SB Force Field to the Stochastic Titration CpHMD Method

**DOI:** 10.1021/acs.jctc.5c00415

**Published:** 2025-06-16

**Authors:** João G. N. Sequeira, Adrian E. Roitberg, Miguel Machuqueiro

**Affiliations:** † BioISI—Instituto de Biossistemas e Ciências Integrativas, Departamento de Química e Bioquímica, Faculdade de Ciências, Universidade de Lisboa, Lisboa 1749-016, Portugal; ‡ Department of Chemistry, 3463 University of Florida, Gainesville, Florida 32611, United States

## Abstract

Incorporating pH into molecular dynamics simulations is vital for accurately capturing the fully coupled conformational, energetic, and protonation landscape of many systems. The constant-pH molecular dynamics (CpHMD) methodologies represent state-of-the-art approaches to achieve this, with stochastic titration CpHMD (st-CpHMD) currently being one of the most well-developed and validated methods. St-CpHMD is already compatible with both the GROMOS 54A7 and CHARMM 36m force fields, and we extend it here to support the AMBER 14SB force field available in the GROMACS software package. We introduce and validate a minor modification to the official atomic partial charges of ff14SB (to achieve neutralization of the main chain) to render them compatible with st-CpHMD, and we benchmark the final implementation using lysozyme and Staphylococcal nuclease proteins. Although the root-mean-square error (RMSE) values of the predictions for p*K*_a_ versus experimental data align closely with those obtained using the other supported force fields, we also identified several challenging cases where the method requires further improvement. AMBER 14SB simulations showed a lower computational cost compared to CHARMM 36m, despite being slightly higher than the GROMOS 54A7 simulations. Our findings also indicate that to further enhance computational speed, future efforts should concentrate on accelerating the PB/MC step. With this extension, we have developed the first CpHMD method implementation compatible with the three most widely used protein force fields, enabling, for the first time, a direct performance comparison among them.

## Graphical abstract

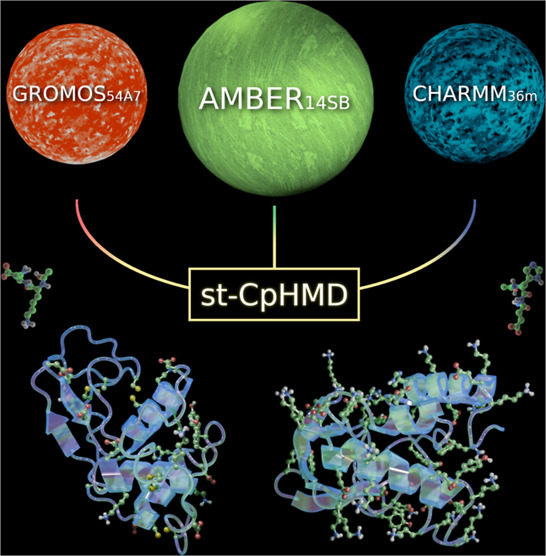

## 1 Introduction

The solution pH is a critical environmental parameter that influences biomolecular systems. By modulating the protonation state equilibria of titratable functional groups within the system, small changes in solution pH can alter the charge distribution within biomolecules, affecting their fundamental structure and function.^1,2^ However, sampling the most representative protonation states at a given pH is very challenging and often leads to pH effects being ignored in molecular dynamics (MD) simulations. Many constant-pH MD (CpHMD) methods have been developed to address these limitations,^2–30^ ^,^ ^31–52^ using different strategies to simultaneously sample the available protonation and conformational spaces according to the semigrand canonical ensemble.^3^ These strategies can be distinguished mainly by (i) type of protonation (continuous vs discrete); (ii) force field used; (iii) approximations used to deal with charge fluctuations in the simulation box (i.e., use of counterions, long-range electrostatics, etc.).^24^ In most discrete CpHMD methods,^2–27,53^ molecular dynamics are periodically interrupted for Monte Carlo (MC) sampling of protonation states, and MD simulations are run in either implicit or fully explicit solvent. Continuous CpHMD methods^28–45,54^ are often based on the λ-dynamics approach for free-energy calculations, where a set of fictitious λ coordinates, with end points (0 and 1) representing the two protonation states, are propagated at the same time as the spatial coordinate.^28^

Most methods are confined to a single software package and/or force field, with only a few exceptions.^7,24,41,43,54^ Comparative studies of p*K*_a_ predictors across various CpHMD methods^55,56^ have been limited, primarily due to limited support across the software packages and force fields used. Notwithstanding, the recent advances in continuous CpHMD methods fully integrated in GROMACS^43,54^ should leverage many of these comparative studies in the future.

In the original implementation of the stochastic titration method (st-CpHMD) by Baptista et al.,^3,5^ the system’s protonation states are periodically updated with those sampled with MC from Poisson–Boltzmann­(PB)-derived energies, with all bonded and nonbonded terms being updated when a protonation/tautomer change occurs. Currently, these implementations are compatible with GROMACS software^57^ and they support both the GROMOS force field family^3,5,58,59^ and CHARMM 36m.^24,60^ The st-CpHMD method has been developed over the last 20 years and currently has two different implementations, based on different PB solvers, MEAD^3,5,22^ and DelPHI.^13,15–17,19,23–27,53^ A major advantage of this method is that it can be used to study complex systems like those involving membranes,^13,15,16,23,27^ nucleic acids,^26^ peptide dendrimers,^25,53^ and ligands/drugs,^19,23,61^ with little effort. Still, the very popular all-atom AMBER force field family,^62^ which has been the one found to better mimic the experimental data of pH-dependent transmembrane proteins^63^ and to better model intrinsically disordered proteins,^64,65^ is yet to be supported. There is an AMBER native implementation of a discrete CpHMD method^4^ that uses the generalized Born (GB) method to obtain the energies used to sample protonation states with MC.^2^ A limitation of the current st-CpHMD implementations is that there cannot be charge propagation between the side and the main chains of the pH-sensitive residues,^5,7^ whereas, with the GB formalism, the whole residue can easily be used to calculate the energies.^66^ Since the charge parametrization procedure of AMBER allows side chain charge propagation to the main chain, until now, direct comparisons between all the supported force fields of the different methods have not been made.

With the AMBER and CHARMM force field families, the electrostatic interactions are primarily treated using the particle mesh Ewald (PME) approach,^67^ which needs system charge neutralization, presenting a significant constraint to its CpHMD implementations^24,36,38,43,68^ as charge fluctuates with residue titration. A straightforward approach is to add a fixed number of counterions to neutralize the solute’s charge at a given pH, bringing the system charge fluctuations near zero.^5,22,24,69,70^ These charge fluctuations can still be corrected using a uniform neutralizing background charge, possibly without creating unwanted artifacts,^71^ a method that is implemented in the GROMACS software package.^57^ There are other methods to ensure complete charge neutrality where, p.e., ions^36,38,43,68^ coupled to titratable sites as charge buffers, some already available in GROMACS.^43^

Our stochastic titration CpHMD method derives all PB parameters from the underlying force field.^24^ However, model compound treatment maintains some theoretical vagueness regarding their molecular definition and p*K*_a_ values.^7^ A site’s model compound is a nonphysical fragment, representing the side chain of an amino acid. The p*K*_a_ value of this model compound (p*K* ^mod^) is then calibrated using experimental data of simple systems.^7^ As previously detailed,^24^ we use the p*K*_a_ values measured from NMR data on alanine-based pentapeptides,^72,73^ thus removing systematic errors introduced by the PB parameters.

The protein hen egg-white lysozyme (HEWL) is a widely used test system for p*K*_a_ predictors, particularly within CpHMD advancements,^2,4,9,17,24,28,33,49,74–77^ with many residues, some of which with highly shifted p*K*_a_ values, having available experimental data.^78–80^ Another commonly used benchmark system is the *Staphylococcus aureus* nuclease (SNase) which also has available experimental data for several residues^81,82^ and some of them being significantly hard to predict.

In this work, we extended our implementation of the st-CpHMD method to the AMBER 14SB^83^ force field. Since charge localization is needed to use PB, and the charge parametrization of AMBER allows side chain charge propagation to the main chain, we propose and validate the impact of a small modification to the official ff14SB atomic partial charges to make them st-CpHMD-compatible. We also compare the results obtained from our simulations with the experimental data of two commonly used protein systems (HEWL and SNase) and to the ones obtained with CHARMM 36m and GROMOS 54A7. This provides the first direct benchmark to compare the three most used force fields in the CpHMD community.

## 2 Methods

### 2.1 CpHMD Extension to AMBER 14SB

To extend the current implementation of the st-CpHMD method^3^ to AMBER 14SB, it is necessary to make this force field compatible with the PB formalism, meaning that no charge propagation should occur between the side and main chains of the titratable amino acid residues. In the previous implementations of this method, with GROMOS 54A7 and CHARMM 36m, the issue did not arise as the charges for each residue were parametrized so that both main and side chains have integer total charges. Since this is not the case with the AMBER family of force fields (Table S1 of Supporting Information), we performed a charge reallocation in all titrating amino acid residues, inspired by the work done in Mongan et al.^4^ To minimize the impact of these changes, we confined the charge transfer solely between the Cβ (and bound hydrogen atoms) to the Cα (and Hα), according to Table S1 of Supporting Information

The AMBER 14SB does not provide amino acid parameters for the neutral forms of their termini. To allow for the pH titration of the termini, we simplified the force field and averaged out the main chain atomic partial charges of all 18 NXXX and CXXX blocks (Prolines and Glycines were excluded), with a charge adjustment in the Cα and Hα to ensure that the N- and C- termini followed the integer charge rule. For the neutral N-termini, the charges of the amine group from the neutral lysine (LYN) in AMBER 14SB were adapted for N, H1, H2, and H3, and the average charges were used in the other atoms, including the previously mentioned Cα and Hα charge correction. For the neutral C-termini, a similar procedure was adopted and the charges of the carboxyl group in the protonated aspartic acid (ASH) were used for C, OC1, OC2, HC11, HC12, HC21, and HC22 atoms. The Cβ and bound hydrogen atoms were also adjusted according to these termini residues to ensure side chain charge localization. For the termini Gly residues, the procedure was similar, although the charged NGLY state was kept in its default form. For the termini Pro residue, the default charges were kept, followed by the charge correction on both the Cα and Cδ, and their bound hydrogen atoms. Likewise, the side chain charge correction was now applied to the Cβ and Cγ and their hydrogen atoms. For the neutral state, the same protocol of using the amino and carboxyl groups from the neutral lysine and protonated aspartic acid was used but, for the remaining atoms, we used the average charges between the N- or C-termini proline block and the regular proline block. This was an approximation that should be corrected when these protonation states are introduced in the AMBER force field. From this point forward, the new charge set will be referred to as st-AMBER 14SB.

To assess the impact of this charge readjustment protocol on the simulations using AMBER 14SB, we used both the GROMACS and AMBER suites to simulate the conformational landscape of dipeptides involving all titratable residues, including the two neutral tautomeric forms of histidine, and two termini-free Alanine dipeptides (Table S2 of Supporting Information). The simulations were performed in triplicates of 100 ns, using both the default (default-amber) and the altered (st-AMBER 14SB) charge sets, and solvated using TIP3P waters,^84,85^ constrained with the SETTLE algorithm^86^ (Table S2 of Supporting Information). The protonation states of the simulated dipeptides were set to the most prevalent ones at physiological pH. To test the new charge set of each residue (excluding the termini), all dipeptides were capped with ACE and NHE groups. A two-step minimization procedure was applied to all systems. The steepest descent minimization algorithm was used with no constraints in the solute for the first step, while the p-LINCS algorithm^87^ was turned on in the second. Each replicate was initialized for 100 ps in *NVT* with an integration step of 1 fs, followed by another 50 ps in *NPT* with an integration step of 2 fs. In the *NVT* ensemble, the v-rescale^88^ (GROMACS) and Langevin^89^ (Amber) thermostats were used to keep the temperature at 310 K (coupling constant of 0.01 ps). In the *NPT* ensemble, the v-rescale thermostat^88^ (coupling constant of 0.1 ps) was used in combination with the Parrinello–Rahman barostat^90^ (coupling constant of 2.0 ps and isothermal compressibility of 4.5 × 10^–5^ bar^–1^) for the GROMACS runs, and the Langevin thermostat^89^ (damping frequency of 1 ps^–1^) combined with the MC barostat^91,92^ were used for the Amber runs.

### 2.2 Protein System Setup

Two proteins were used to test the AMBER FF extension to the st-CpHMD method (Table S3 of Supporting Information): Lysozyme (HEWL; pdb id: 4LZT)^93^ and *S. aureus* nuclease (SNase; pdb id: 1STN).^94^ All systems were solvated in a rhombic dodecahedral box with periodic boundary conditions. The PypKa Web server^95,96^ was used to estimate an initial number of counterions to add at each pH value (Table S4 of Supporting Information) and all charge fluctuations were corrected by the PME background charge correction in GROMACS. It should be noted that in a significant amount of the simulation time, the system had a non-neutral charge. However, with our approach, the system’s total charge usually only varied between ∼±3, which is not expected to create unwanted artifacts.^71^

In parallel to CpHMD and to further test the impact of this new charge set on the sampled conformational landscape, 1 μs long MD simulations were done using both the new and the default charge sets. These simulations were run on the *Gallus gallus* lysozyme (PDB ID: 4LZT), in a system solvated with 6.0k TIP3P waters^84,85^ and 9 Cl^–^ ions in a rhombic dodecahedral box with periodic boundary conditions.

### 2.3 Constant-pH MD Settings

All systems were simulated in triplicate for 100 ns, across all integer pH values ranging from 1 to 12, underwent a two-step minimization procedure, and were initialized as described for the GROMACS simulations in Section 2.1.

In the CpHMD scheme, production MD simulations were interrupted every 20 ps and new protonation states, including PB-derived free-energy terms, were proposed as MC candidates for all possible titrating residues in the systems.^3,5,17^ If a protonation state has been accepted, and before the subsequent production of MD segments, a brief solvent relaxation step (0.2 ps) was conducted with a frozen solute to allow the solvent to adjust to the new protonation states. All CpHMD simulations were performed using the AMBER ff14SB force field (with st-CpHMD adapted charges) and with an integration step of 2 fs using GROMACS 2021.5.^57^ The nonbonded interactions were treated with a single cutoff of 1.0 nm, updating the neighbor list every 10 steps. Beyond the cutoff, all van der Waals interactions were truncated, and the Coulombic interactions were treated with the particle mesh Ewald (PME) method,^67^ using a Fourier space grid of 0.125 nm. The systems were considered to equilibrate within the initial 30 ns (see “System Equilibration and Stability” section). Hence, only the 30–100 ns segment was used for production analysis (see Results and Discussion), except where stated otherwise.

PB calculations were performed using DelPhi v5.0 ^97^ using a two-focusing procedure.^98^ Dielectric constants of 80 for solvent and 2 for protein (to attenuate the absence of polarization effects in fixed charge models)^3,5^ were considered. The focusing procedure used a grid space of 0.1 nm for the larger grid, which was reduced to 0.025 nm in the smaller (focusing) grid. Both grids were centered in the titratable group and contained 81 grid nodes on each side. The convergence threshold was set to 0.01 *k*_b_ *T*/e.^99^ The MC runs were performed, using PETIT v1.6.1,^100^ for 10^5^ cycles, with random attempts made in each cycle to change the protonation state of every titrable group and of all pairs of interacting sites (PB-interaction energies >2 p*K* units).

### 2.4 MD Settings

All long MD simulations were performed using GROMACS 2021.5,^57^ both with the modified and the default AMBER ff14SB force fields,^83^ and the TIP3P water model.^84,85^ We simulated triplicates of 1 μs each, with a 2 fs integration step, of systems that underwent a two-step minimization procedure and were initialized as described for the GROMACS simulations in Section 2.1. The nonbonded interactions were treated with a single cutoff of 1.0 nm, updating the neighbor list every 20 steps. Beyond the cutoff, all van der Waals interactions were truncated, and the Coulombic interactions were treated with the Particle-Mesh Ewald (PME) method^67^ using a Verlet scheme cutoff of 1.0 nm and a Fourier grid spacing of 0.125 nm. The LINCS^87^ and SETTLE^86^ algorithms were used to constrain bond length between H and heavy atoms of solute and water molecules, respectively.

### 2.5 p*K* ^mod^ Calibration

A model compound shares a chemical group with a titratable site in the protein, for which p*K*_a_ values are known.^7^ Here, the model compounds are fragments of the titratable amino acid residues, and their p*K*_a_ values (p*K* ^mod^) require calibration to the AMBER ff14SB force field. This was done using a previously reported protocol^7,16,24^ based on experimental data of Alanine-based pentapeptides (Ala_2_-X-Ala_2_, where X is a pH titrating residue—i.e. Asp, Cys, Glu, His, Lys or Tyr)^72,73^ with capped termini. The calibration procedure consisted of running CpHMD simulations (3 replicates of 50 ns) at pH values near an initial p*K* ^mod^ guess and obtaining a p*K*_a_ shift, based on the difference between the experimental p*K*_a_ values and the complete pH titration curve of the simulated pentapeptide, which allows the calculation of the final p*K* ^mod^ (Table S5 of Supporting Information). As was the case with both previously st-CpHMD supported force fields,^24^ both termini show significant shifts, associated with the model compound definition. Noteworthy, the largest shift obtained was with the cysteine residue, something that could be also related to the poor description of solvation around the thiolate anion in the AMBER force field,^101^ that lacks specific Lennard-Jones parameters for thiolate sulfur.^102^ Most of these limitations are systematic and should be corrected in the used p*K* ^mod^ calibration step.

### 2.6 Analyses and Error Calculations

Analyses were performed using GROMACS,^57^ AmberTools21 ^62^ and in-house tools, visualized with PyMOL^103^ or Visual Molecular Dynamics (VMD),^104^ and plotted with Gnuplot.^105^ The error values were calculated using the standard error of the mean from the 3 replicate simulations. In the case of p*K*_a_ values, we used a leave-one-out approach (jackknife) in the Hill curve fitting procedure to estimate the error values.^106^

## 3 Results and Discussion

### 3.1 St-AMBER 14SB Validation

#### 3.1.1 Dipeptide MD Simulations

An important objective of this work was to test the newly developed AMBER 14SB charge set that will be used in the st-CpHMD method. We have tested the impact of this new charge set on the conformational landscape sampled in several systems, including different dipeptide combinations of titratable amino acids. In the analysis of the distance between the side chains of the different residues (Figures S1 and S2 of the Supporting Information), the pseudodihedral between Cβ–Cα–Cα–Cβ (Figures S3 and S4 of the Supporting Information) and the Φ and Ψ dihedrals (Figures S5 and S6 of the Supporting Information) the sampled landscape was very similar between the charge sets, with Kullbach–Leibler divergence (*D*_KL_) values^107^ consistently below 0.04. Since the distribution of the Cβ–Cα–Cα–Cβ pseudodihedral appeared to be consistently bimodal across all simulated dipeptides and regardless of charge set, using the distribution of this angle across all simulations (Figures S7 and S8 of the Supporting Information) we were able to determine the regions [−180, −40.5] and [118.5, 180] as one region (angle1) and [−40.5, 118.5] as the other (angle2). Looking at the prevalence of each angle region in the simulations with different charge sets (Tables S6 and S7 of Supporting Information), it is seen that different charge sets used did not appear to lead to large discrepancies (average difference between the simulations with distinct charge sets is just 3% and 4% for the GROMACS and Amber runs, respectively).

The potential energies landscapes of the dipeptides “in a vacuum” (i.e., of the dipeptides without any solvent influence) were obtained using 1000 structures (one every 0.1 ns) from each replicate and showed very similar profiles regardless of charge set and simulation suite used (Figures S9 and S10 of Supporting Information). There are some cases where the landscape appears to be slightly shifted (around 50 kcal/mol) depending on the charge set (p.e. NGLU-CHID in the GROMACS runs) (Figure 1A,B). This small difference is completely dissipated in the distribution fluctuations once the solvent is considered in the energy calculations (Figures 1C,D, and S11 and S12 of Supporting Information). In summary, the distributions are very similar (very low *D*_KL_ values) across charge sets, simulation suites, and dipeptides, further supporting that the new charge set is fully compatible with the AMBER 14SB official force field.

**Figure 1:**
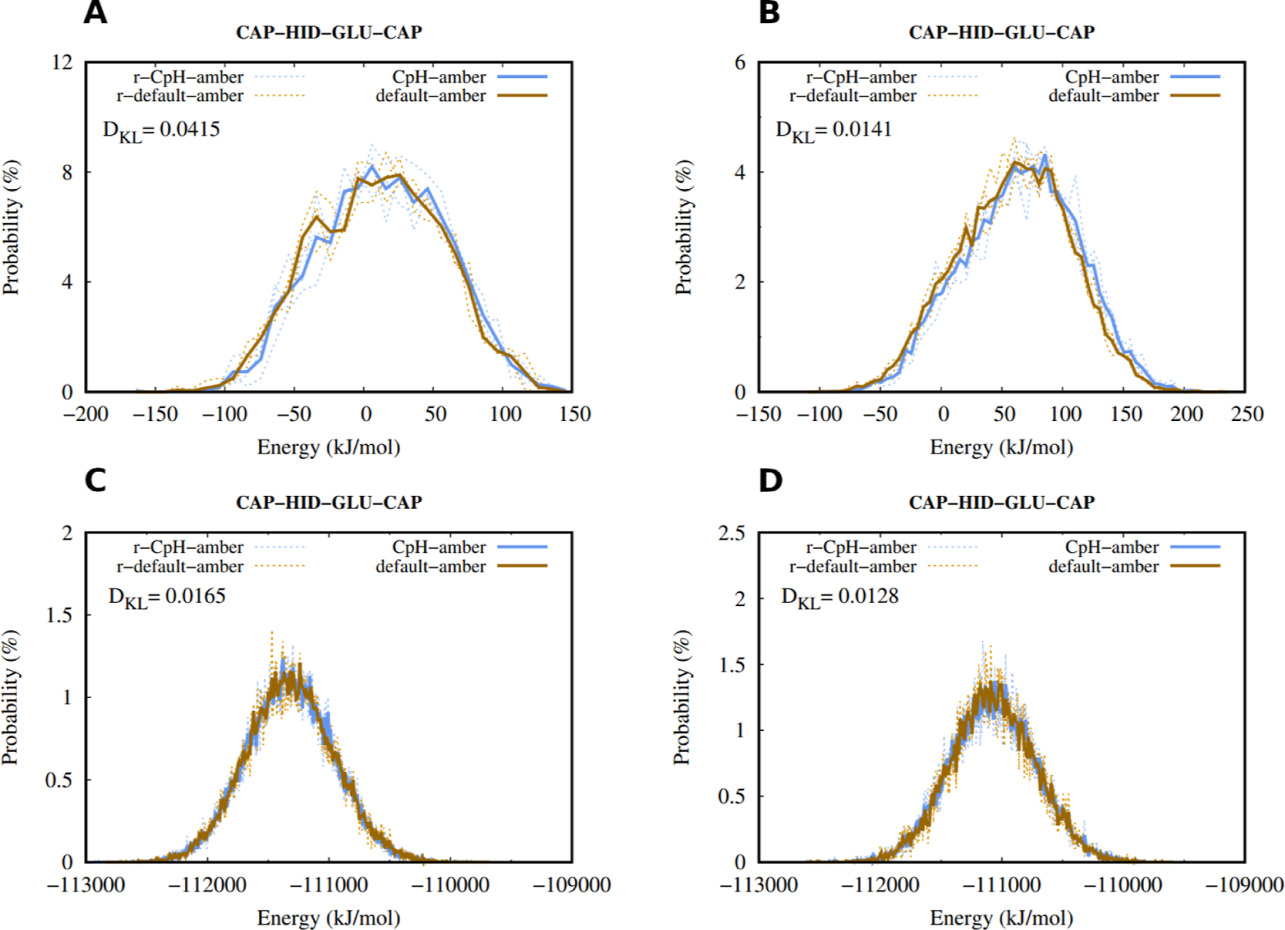
Histogram of the dipeptides potential energies “in vacuum” (A,B) and in solvent (C,D), of both GROMACS (A,C) and Amber suite (B,D) simulations. The blue line depicts the simulations with the changed force field, and the brown line represents the default force field. The solid lines represent the averages between all replicates (dotted lines). Kullback–Leibler divergence values^107^ are shown for each distribution.

To evaluate the impact of the new charge set on the simulation convergence, we used the *D*_KL_ values over time calculated on the distributions of the side chain distance (Figures S13 and S14 of Supporting Information). They were calculated by iteratively computing the *D*_KL_ values from 0–*t*, with *t* ranging from 0.01 to 100 ns, in 0.01 ns steps. These cumulative profiles show that the two distributions converge very fast and that within ∼20 ns the two charge sets become indistinguishable. This fast equilibration, and the robustness of the obtained distributions, can be illustrated by splitting the simulations in halves (instead of the original thirds—triplicates) and reevaluating the conformational landscape of the structural analysis already described. This showed that, similar to triplicates, the variance between the halves is nearly equivalent to the variance observed among simulations using different charge sets (see Figure S15 in the Supporting Information), with the sampled landscape being similar, regardless of the charge set.

#### 3.1.2 Assessing Secondary Structure with Long MD Simulations

The secondary structure propensity of proteins is a very important property not properly captured by our small dipeptide simulations (Section 3.1.1). Since the charge reallocation protocol involved changes in the atomic partial charges of Cα and Cβ (and their bound hydrogen) atoms of the titratable residues, the protein’s overall helicity propensity may have been slightly affected. To check this, we ran long MD simulations (3 × 1 μs) of HEWL using both the default and the st-AMBER 14SB charge sets. The st-AMBER 14SB charge set did not appear to have a significant impact neither in the RMSD equilibration (Figure 2A) nor in the secondary structure propensity (Figure 2B) of the simulations.

**Figure 2:**
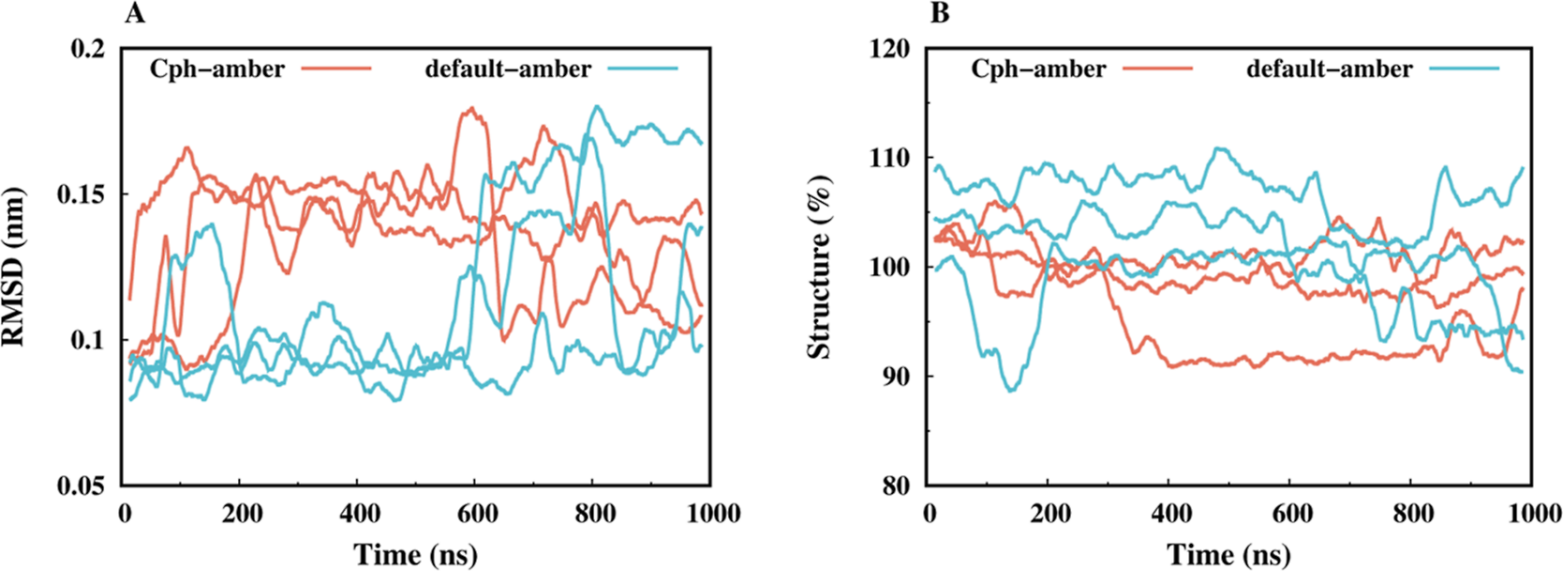
Cα RMSD vs the X-ray structure (A) and Secondary structure (B) values, along the simulation, for both simulated charge sets (pink for the new charge set and blue for the default), using sliding windows (1 ns) averages. Structure represents the sum of all residues in helical or β-sheet conformations, and the percentages were calculated assuming 100% for the experimental structures.

### 3.2 AMBER 14SB Constant-pH MD Benchmark

#### 3.2.1 System Equilibration and Stability

Before extracting equilibrium properties, such as p*K*_a_ values, we have to ensure that our system is properly equilibrated in the simulations across all simulated pH values. We computed the Cα RMSD vs the simulations’ initial structure and monitored secondary structure profiles across all replicates and pH values (Figures S16–S19 of the Supporting Information). The simulations showed, for all systems and pH values (including extreme pH conditions where possible unfolding is avoided due to the short length of the simulation), similar property variations, with most simulations displaying good convergence after 30 ns of simulations.

We examined the equilibrated part of the simulations (30–100 ns) by calculating the aforementioned structural properties across the range of simulated pH values (Figure 3). The HEWL exhibited consistently higher RMSD values compared to the SNase across all pH levels (Figure 3A), but with little to no impact on the secondary structure content (Figure 3B), suggesting good overall protein stability, even at extreme pH values, probably due to the short nature of our MD simulations. The SNase is known to be a highly stable protein,^108^ which is very well captured in our simulations.

**Figure 3:**
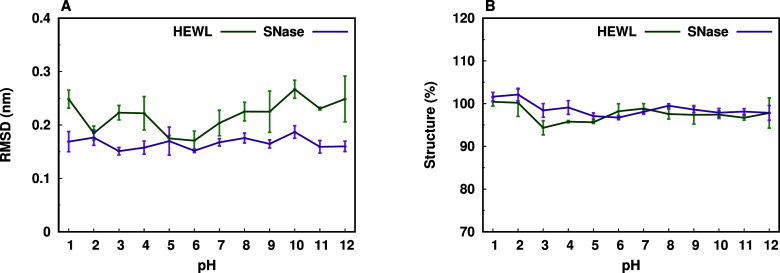
Average Cα RMSD (A) and secondary structure (B) values per pH. Data is shown for HEWL and SNase. The structure represents the sum of all residues in helical or β-sheet conformations, and its percentages were calculated by comparing to the experimental structures.

We also assessed the protonation equilibration/convergence and illustrated it with data from Glu-35 of HEWL, which is a residue known to have its p*K*_a_ significantly shifted and is relatively difficult to predict (Figure S20 in Supporting Information). Our results show a good convergence after ∼30 ns of simulation, even for such a difficult case.

#### 3.2.2 Total Titration Curves

We calculated the total titration curves of both proteins from CpHMD simulations and compared them with the experimental data and with those previously obtained with the GROMOS 54A7 and CHARMM 36m force fields^24^ (Figure 4).

**Figure 4:**
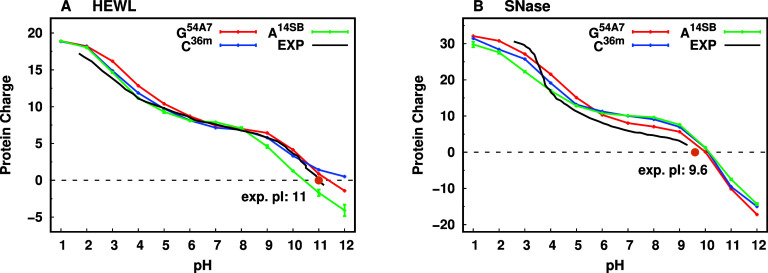
Full titration curves obtained for (A) HEWL and (B) SNase using the three st-CpHMD supported force fields (AMBER 14SB in green, GROMOS 54A7 in red^24^ and CHARMM 36m in blue^24^). The available experimental titration curves are plotted as black lines,^109,110^ while the isoelectric points for each protein are represented with an orange dot.

Overall, there is a very good agreement between the titration curves and isoelectric points (pI) (Table S8 of Supporting Information) obtained from the AMBER 14SB force field simulations, the experimental data, and the simulations with the other force fields. Notably, the AMBER 14SB force field struggled in predicting the HEWL experimental values at higher pH values (>9.0) (Figure 4A and Table S9 in Supporting Information), possibly as a consequence of the AMBER force field struggling to correctly predict the protonation of both lysine and tyrosine residues. Similar to what we observed for the CHARMM 36m force field,^24^ the AMBER 14SB also overestimates the neutral forms of these residues, and since there are more Lys residues (6) than Tyr (3) in HEWL, the final overall effect is a decrease in the total charge in the alkaline pH range. The SNase protein is a very challenging system for all computational methods since it exhibits a significant number of residues that have their p*K*_a_ values either shifted or not shifted due to different electrostatic effects counteracting each other, which renders them very difficult to predict. The AMBER 14SB performs similarly to the other force fields (Table S10 of Supporting Information), with some noticeable deviations to the experimental curve (Figure 4B). Notwithstanding, the overall performance is quite good, possibly also due to the many residues charges, with counteracting effects, influencing the titration curves and pI predictions.

#### 3.2.3 p*K*_a_ Predictions Benchmark

We calculated the p*K*_a_ values of the individual residues for the studied proteins using st-AMBER 14SB CpHMD simulations (Figures S21 and S22 of Supporting Information). Experimental data are available for some of these residues,^111^ allowing for direct comparisons (Tables S9 and S10 of Supporting Information) and to benchmark the method’s predictive ability (Table 1).

**Table 1:** RMSE of the p*K*_a_ Predictions Using the Null Model and the AMBER 14SB CpHMD Simulations, in Comparison to the Experimental Values (Tables S9 and S10 of Supporting Information)^b^

system	null	PypKa	AMBER 14SB			CHARMM^24^	GROMOS^24^
			[0–5]	[5–30]	[30–100]		
HEWL	1.27	0.71	0.83	0.82	0.91	0.99	1.00
SNase	0.76	1.25	1.16^a^	0.85^a^	1.68	1.46	1.22

a Obtained excluding His-46, since this residue does not titrate in these simulation segments.

b The conformational/protonation sampling was split into three time segments: [0–5], [5–30], and [30–100] ns, for the non-equilibrated and the equilibrated parts of CpHMD simulations, respectively. Tyrosine residues were excluded from this calculation as their p*K*_a_ values were often outside our pH range. A total of 18 and 17 experimental p*K*_a_ values were considered for HEWL and SNase, respectively.

The RMSE metric quantifies the deviation between observed and predicted values. The Null model RMSE is obtained using the water p*K*_a_ values of each residue type in the Ala-based pentapeptides, hence, a control independent of protein structure. We also show the RMSE values calculated from the p*K*_a_ predictions using the PypKa software.^95,112^ This tool uses PB/MC calculations on rigid structures, hence depending heavily on the representativeness of the selected X-ray structure. We also split the CpHMD sampling into three segments. The two initial ones (0–5 ns and 5–30 ns) are with the protein still equilibrating and rearranging its structure. These have been shown to produce the best p*K*_a_ predictions when using the GROMOS force field family.^7^ The final segment (30–100 ns) corresponds to where the proteins’ conformational space is equilibrated and converged. To assess the quality of our system equilibration, we also calculated the RMSE values for a 50–100 ns segment, and the results did not change for HEWL (0.91 vs 0.92) and changed only moderately for SNase (1.68 vs 1.57). In addition, we calculated the p*K*_a_ values in 5 ns windows over time for all residues to check their convergence in the 30–100 ns segment (Figures S23 and S24 of Supporting Information). This analysis confirms that, excluding a very few challenging cases, the p*K*_a_ convergence of the titrating residues is very good, which increases the overall accuracy of the method. Our results with the AMBER 14SB force field are similar to the ones previously described for the other supported force fields.^24^ The HEWL protein, despite its high null model value, has a very representative crystal structure, which results in the PypKa estimation outperforming the CpHMD predictions. Furthermore, the best performance of CpHMD happens at the beginning of the simulation, confirming that the protein conformational space being sampled is impairing the p*K*_a_ predictions. The SNase protein has a remarkably low null model RMSE (0.76), pointing toward an easier prediction. However, the PypKa and the CpHMD AMBER 14SB predictions are not as accurate, suggesting poor representativeness of the experimental crystal structure and a clear inability of the CpHMD method to correct it within 100 ns of simulation. Additionally, we did not see protonation changes for a particular challenging residue (His-46, see Section 3.2.4) within the initial 30 ns of simulation, meaning that the predicted RMSE value for this segment cannot be directly compared to that of the equilibrated portion.

We generated p*K*_a_ values scatter plots to evaluate the agreement between AMBER 14SB and the experimental data or the predictions with the other force fields (Figure 5). We observe very good agreement with the experimental data and an excellent agreement with the other two force fields. The AMBER 14SB predictions for both termini were very accurate, outperforming both CHARMM and GROMOS values, which strengthened our protocol to create the termini neutral forms. Overall, we can see small trends in some residues where AMBER 14SB overstabilizes their neutral forms (Asp, His, and Lys), a phenomenon already observed for CHARMM 36m.^24^ This is also confirmed by the fact that the AMBER predictions are in excellent agreement with those obtained with CHARMM.

**Figure 5:**
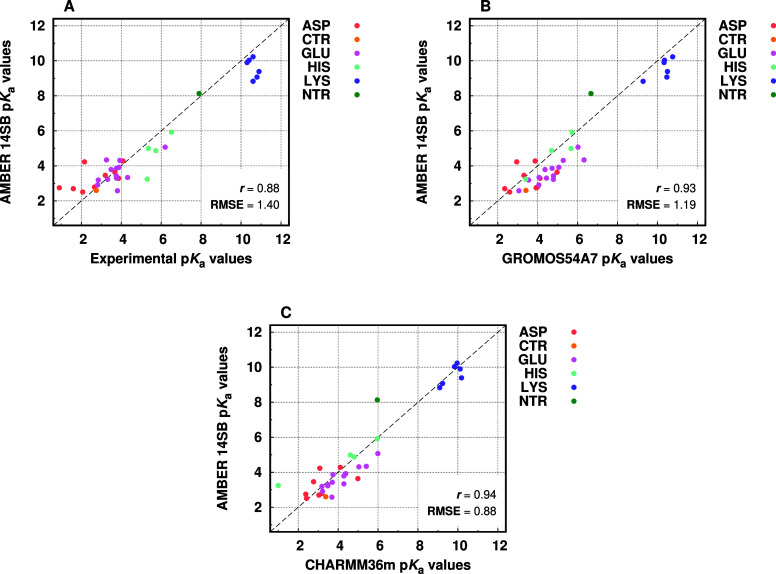
Scatter plots comparing AMBER 14SB with experimental data (A) and the predictions using GROMOS 54A7 (B) and CHARMM 36m (C). The Person’s correlation coefficient and the RMSE values are also shown.

We calculated the RMSE values and the mean error for each type of titrating residue and observed that some have easier-to-predict p*K*_a_ values (Table S11 in Supporting Information). The usually well-solvated residues, which are mostly charged at physiological pH (i.e., CTr, Asp, Glu, NTr, and Lys) and likely have their p*K*_a_ values closer to the reference, displayed lower RMSE values. The His and Tyr residues tend to be in their neutral form, internalized and thus are a greater challenge for the p*K*_a_ calculations. These had the largest RMSE of all residues, with the Tyrosine residues p*K*_a_ values often being extrapolated (>12). The cysteines, for which no experimental values existed in the simulated proteins, will be further studied in future efforts to test the three st-CpHMD-supported force fields.

The mean error of each residue type shows that the anionic residues Glu and CTr have a downshift toward the deprotonated state, most abundant at pH = 7.0, whereas Asp and Tyr have an upshift toward the protonated state. The cationic residue NTr also has a positive shift, whereas His and Lys have negative p*K*_a_ shifts, possibly due to an overstabilization of the neutral form of these residues. These effects have been observed in other force fields^24^ and are most likely related to their parameters (interactions with TIP3P water) or the PB model and the used atomic radii. Our p*K*_a_ predictions were particularly poor for the Tyr and His residues, likely as a consequence of very shifted residues in the simulated systems for which there is very little experimental data. Future studies with a larger protein set will be needed to more accurately benchmark the st-CpHMD method with some of these residue types.

#### 3.2.4 Challenging Cases

Several challenging residues proved to be difficult to predict since the titrating groups are either trapped in nonrepresentative conformations or have experimentally measured shifted p*K*_a_ values: Lys-1, Tyr-23, Lys-33, Glu-35, Asp-66, and Lys-96 in HEWL (Table S9 of Supporting Information) and His-46, Glu-75, Asp-95, and His-121 of SNase (Table S10 of Supporting Information). We analyzed in detail some of these cases, including Glu-35 of HEWL and Glu-75 of SNase, which are considered a difficult cases and have been extensively studied by many methodologies in different studies.^7,24,36,41,113–115^

Rigid body p*K*_a_ calculations often struggle with the HEWL Glu-35 residue,^113^ with an increase in the dielectric constant of the protein in PB-based predictors commonly leading to a better prediction of water-facing residues while worsening the estimations of the less abundant catalytic sites. This residue is located in the catalytic cleft of the protein and has a shift of ∼2 p*K* units in its experimental p*K*_a_ (6.2). The AMBER 14SB had the worst p*K*_a_ predictions of the supported force fields for this residue (Table S9 in Supporting Information) despite being better than the rigid body predictions made with PypKa.^24^ This suggests that the structural rearrangement needed for accurately determining this residue’s p*K*_a_ may happen slower with the AMBER force field. To check the convergence rate for this challenging case, we computed the cumulative average for the protonation at pH 5 and 6 (closest to the estimated p*K*_a_) and saw a good convergence after 30 ns of simulation (Figure S20 of Supporting Information), reinforcing that the problems with predicting experimental p*K*_a_ for this residue may be related to slow structural rearrangements.

Another contributing factor to AMBER’s poor performance with histidines was the His-46 of SNase. This extremely shifted residue (−6.61 p*K*_a_ units) has two very close lysines (Lys 48 and Lys-49, Figure 6A) which interact with the histidine (Figures 6B and S25 of Supporting Information) and could explain the overstabilization of its neutral form.

**Figure 6:**
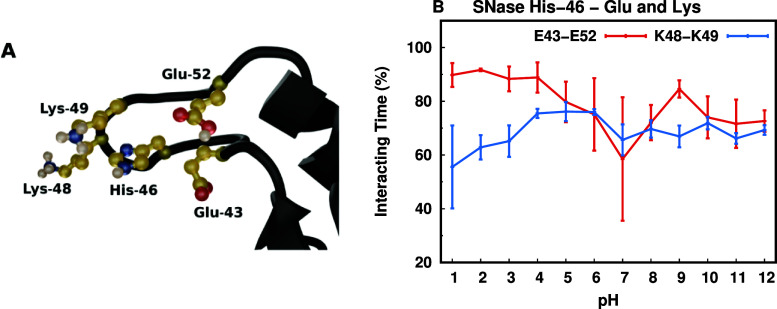
SNase His-46 case study. (A) Structural representation of the SNase’s loop (in black) where His-46 interacts with Glu-43, Lys-48, Lys-49 and Glu-52 (in yellow). (B) Interaction time (%) between His-46 and either Lys-48 or Lys-49, and Glu-43 or Glu-52 across pH values. Interactions were considered at distance <0.6 nm between side chains (see distance distributions in Figure S25 of Supporting Information).

Notwithstanding, at lower pH values, the His-46 can also interact with two nearby glutamic acid residues (Glu-43 and Glu-52, Figure 6A), which should counteract the previous effect and help increase its p*K*_a_ value. However, when acidifying, these two acidic residues protonate before His-46 (Table S10 of Supporting Information), removing the local negative charges from the electrostatic balance that is needed to protonate and positively charge this histidine. This phenomenon may have been triggered by a conformational trap that exists in the crystal structure, from which the AMBER 14SB force field simulations struggle to escape. This is supported by the fact that the rigid body p*K*_a_ predictor PypKa, also fails to predict the p*K*_a_ value of this residue (−4.63 p*K*_a_ units away from experimental^24^). As a consequence, His-46 stands out as the worst predicted residue in the AMBER force field (Table S10, in Supporting Information). To test the conformational bias in the X-ray structure, we ran 3 × 100 ns simulations at pH 2 starting from a structure where His-46 is protonated and away from the two lysines and glutamic acids, following the same described protocol. These new simulations showed a significantly lower interaction with both lysines (Figure S26 of Supporting Information) and resulted in the full protonation of His-46, which is in agreement with the expected experimental p*K*_a_ value. This example highlights the difficulties experienced when trying to predict p*K*_a_ values using some force fields in relatively short time scales.^24^ Although it is expected that most of these discrepancies will be corrected by using longer runs, realistically, we should be aware of these limitations and take mitigating actions when setting up our CpHMD simulations.

Residues Glu-75 and His-121 of SNase are typically buried and interact consistently^24^ across several pH values (Figure 7A). The experimental p*K*_a_ values of Glu-75 (3.26) and His-121 (5.30) indicate that, in solution at pH 3.5–5.0, these residues likely establish either a salt bridge or trigger a local structural rearrangement that promotes their solvation, still holding their charged states. When interacting at 3.5< pH < 5.0, this anionic-cationic residue pair can be stabilized by either a salt bridge (double-charged interaction) or a hydrogen bond. Therefore, to accurately predict the experimental p*K*_a_ values of these residues, we need to sample correctly the balance between these two populations. We computed the average protonation state of each residue across the simulated pH values (Figure 7B), for the equilibrated part of the simulations.

**Figure 7:**
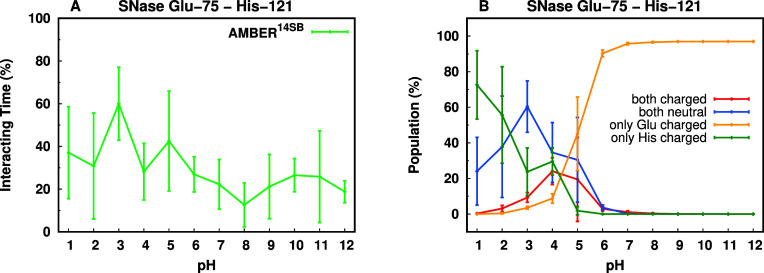
SNase Glu-75 and His-121 case study. (A) Interaction time (%) between Glu-75 and His-121, for all simulated pH values. Interactions considered a 0.4 nm cutoff distance between side chains.^24^ (B) Distribution of protonation states for Glu-75 and His-121 across simulated pH values.

In the pH range near the two p*K*_a_ values, we observed a quite small sampling of the double-charged species, which helps explain the inversion of predicted p*K*_a_ values (4.34 for Glu-75 and 3.23 for His-121; Table S10 of Supporting Information). This similar trend was seen previously in simulations with both GROMOS and CHARMM force fields, which captured even fewer double-charged conformations.^24^ The AMBER force field resulted in a better prediction of Glu-75 p*K*_a_ value over the other force fields, but His-121 p*K*_a_ was still largely underestimated. It seems that the overstabilization of neutral histidine residues, which leads to poor p*K*_a_ prediction performance (Table S11 in Supporting Information), is a limitation in our CpHMD approach that seems to be common to all three force fields studied. Strategies aimed at solving this and other similar issues are now being deployed, focusing on our radii calculation procedures^116^ that most likely need to be calibrated on a larger protein set.

### 3.3 Computational Efficiency

The computational efficiency of the stochastic titration CpHMD method using the three supported force fields was evaluated systematically in a controlled benchmark (Table 2).

**Table 2:** Simulation Speed Benchmark (ns/day) for the GROMOS (54A7), CHARMM (36m), and AMBER (14SB) Force Fields^a^

system	# residues (titrable)	FF	GPU			CPU		
			CpHMD (ns/day)	PB/MC	MD	CpHMD (ns/day)	PB/MC	MD
HEWL	129 (21)	GROMOS	65.6 ± 2.9	64.8%	30.6%	24.8 ± 0.3	24.0%	73.8%
		CHARMM	54.1 ± 0.2	62.9%	31.6%	23.1 ± 0.1	26.7%	71.0%
		AMBER	61.4 ± 0.3	68.3%	26.9%	33.6 ± 0.1	36.9%	60.4%
SNase	141 (50)	GROMOS	45.8 ± 0.2	77.2%	19.1%	21.8 ± 0.2	36.3%	61.8%
		CHARMM	36.6 ± 0.2	73.6%	21.6%	19.6 ± 0.1	39.3%	58.3%
		AMBER	39.9 ± 0.3	78.7%	17.5%	26.6 ± 0.0	52.4%	45.3%

a Time allocation for PB/MC and MD step is detailed, with relaxation taking ∼8% (GPU) and ∼3% (CPU) of simulation time. File handling (I/O) accounted for ∼5% (GPU) and ∼3% (CPU). Benchmarks were conducted on an 8-core setup within a 24-core machine with three Intel­(R) Xeon­(R) Silver 4310 CPU @ 2.10 GHz processors. GPU runs used an NVIDIA GeForce RTX 3060 Ti GPU on top of the previous setup.

We used both HEWL and SNase, which have different numbers of residues (titrable and nontitrable). The simulations were faster for the smaller protein (HEWL) and, unsurprisingly, the force field that allowed for the faster simulations was the united-atom GROMOS 54A7, likely as a consequence of the smaller number of atoms in the system. The AMBER 14SB simulations were faster than the CHARMM 36m. This can be explained by the long-range electrostatics cutoff difference between the force fields (1.0 nm for AMBER and 1.2 nm for CHARMM), as there are fewer direct interactions considered in the AMBER simulation. Despite this, the smaller long-range electrostatics cutoff of AMBER in comparison to GROMOS (1.4 nm) was not enough to overcome the number of atoms difference. Since the PB/MC step is constant and only runs on CPUs, it becomes the rate-limiting step in the GPU-accelerated simulations, especially for systems with many titrating sites—the computational cost of this step increases linearly with the number of titratable residues. Therefore, we have been putting a large effort into speeding up the protonation state predictions, including taking advantage of ML-based methods, like pKAI.^117^

## 4 Conclusions

Until now, our st-CpHMD method implementation was compatible with the GROMOS and CHARMM force field families in the GROMACS software package. Here, we have extended the method to enable CpHMD simulations using AMBER 14SB. Since the charge parametrization procedure of this force field does not enforce charge neutrality to the residues’ main chains, we proposed a small modification to the official ff14SB atomic partial charges to make them st-CpHMD-compatible. Using simulations of dipeptide combinations with all titrating residues (and termini), we showed that this modification did not alter significantly the conformational and energetic landscape of the official force field. Long MD simulations of HEWL with the modified charge set also showed no measurable impact on the secondary structure.

The new CpHMD implementation was used to simulate two of the most commonly used proteins in p*K*_a_ prediction benchmarks: HEWL and SNase. Both were conformationally stable in the simulations, even at extreme pH values, with the p*K*_a_ prediction accuracies decreasing with simulation time, albeit for distinct reasons. HEWL’s initial structure is very representative, yet the protein structure loses stability over time at extreme pH values,^17^ making it difficult to improve the p*K*_a_ predictions. SNase has several challenging residues to predict and a less representative crystal structure, despite the Null model’s excellent performance. Altogether, these are quite challenging systems, and the RMSE values obtained with our AMBER 14SB implementation align well with those reported in the literature using different methods and force fields.^7,9,17,41^

In terms of computational efficiency, AMBER 14SB simulations were faster than the ones using CHARMM 36m, likely due to the smaller long-range electrostatics cutoff employed in AMBER. However, GROMOS 54A7 simulations remained the fastest overall, owing to the united-atom nature of this force field. Across all GPU-accelerated simulations, the PB/MC step becomes the most computationally expensive. Hence, to increase the method’s performance, particularly for larger systems (with many titrable residues), efforts should prioritize accelerating the protonation state predictions.

## Supplementary Material





## Data Availability

The GROMACS package is freely available software used to perform MD simulations and can be downloaded at https://manual.gromacs.org/2021.5/download.html. PyMOL v3.1 is also free software for molecular visualization and generating high-quality images. It can be downloaded from https://pymol.org.

## Notes

Code to run the CpHMD simulations, the modified AMBER 14SB files, the system’s final configurations, and the topologies (ZIP)
Main chain charge of the different titratable amino acids residue blocks in the AMBER14SB force field (including those in the N– and C– termini). These represent how much charge needs to be reallocated to allow for charge neutralization of the main chain, simulated dipeptides and number of water molecules used in the simulated system (PDF)
