# Supplementary material for: Adding the AMBER 14SB Force Field to the Stochastic Titration CpHMD Method
Source: J Chem Theory Comput. 2025 Jun 16;21(13):6292–304. doi: 10.1021/acs.jctc.5c00415 (PMC12818765; doi:10.1021/acs.jctc.5c00415)
Supplement: Supplementary file 2 [file ct5c00415_si_002.pdf]

Supporting Information: Adding the AMBER 14SB force field to the stochastic titration CpHMD method

João G. N. Sequeira,[†] Adrian E. Roitberg,[*,‡] and Miguel Machuqueiro[*,†]

†*BioISI – Instituto de Biossistemas e Ciências Integrativas, Departamento de Química e Bioquímica, Faculdade de Ciências, Universidade de Lisboa, 1749-016, Lisboa, Portugal*

‡*Department of Chemistry, University of Florida, Gainesville, Florida 32611, United States*

E-mail: roitberg@ufl.edu; machuque@ciencias.ulisboa.pt

Phone: +351-21-7500112

Table S1: Main chain charge of the different titratable amino acids residue blocks in the AMBER14SB force field (including those in the N– and C– termini). These represent how much charge needs to be reallocated to allow for charge neutralization of the main chain.

Aminoacid	Main Chain Charge	C-ter	Main Chain Charge	N-ter	Main Chain Charge
HID	-0.01	CHID	-0.02	NHID	-0.02
HIE	-0.04	CHIE	-0.04	NHIE	-0.05
HIP	0.06	CHIP	0.06	NHIP	0.05
TYR	-0.03	CTYR	-0.04	NTYR	-0.05
GLU	-0.12	CGLU	-0.12	NGLU	-0.13
ASP	-0.14	CASP	-0.14	NASP	-0.15
LYS	-0.03	CLYS	-0.02	NLYS	-0.02
CYS	0.02	CCYS	0.01	NCYS	0.01
GLH	-0.02				
ASH	0.01				
LYN	-0.09				

Table S2: Simulated dipeptides and Number of Water Molecules used in the simulated system.

Dipeptide	# waters
ACE – Lys – Asp – NHE	2431
ACE – Hid – Glu – NHE	2797
ACE – Hie – Glu – NHE	2752
ACE – Cys – Tyr – NHE	2104
NAsp – CLys	2308
NCys – CTyr	2033
NGlu – CHid	2071
NGlu – CHie	2076
NHid – CGlu	2707
NHie – CGlu	2436
NLys – CAsp	2338
NTyr – CCys	2611
ACE – Ala – CAla	2395
NAla – Ala – NHE	1910

Table S3: Proteins tested with their corresponding PDB codes and the number of residues/water molecules in the simulated system. The number of ions added per pH value is shown in Table S1 of the Supporting Information. The water molecule number variation in these systems is due to the addition of counter-ions.

System Name	Protein name	PDB ID	#AAs	#waters
HEWL	*G. gallus* Lysozyme	4LZT	129	6.0k
SNase	*S. aureus* Nuclease	1STN	136	5.6k

Table S4: Number of ions used in the CpHMD simulations, with pH ranging from 1-12, for both simulated systems.

Protein	pH											
	1	2	3	4	5	6	7	8	9	10	11	12
HEWL	18 Cl^-	16 Cl^-	13 Cl^-	10 Cl^-	9 Cl^-	8 Cl^-	7 Cl^-	7 Cl^-	6 Cl^-	4 Cl^-	1 Cl^-	1 Na^+
SNase	28 Cl^-	24 Cl^-	19 Cl^-	15 Cl^-	13 Cl^-	12 Cl^-	10 Cl^-	9 Cl^-	7 Cl^-	3 CL^-	6 Na^+	14 Na^+

Table S5: Model compounds pK_a values for each titrable residue. The experimental pK_a values obtained for the Alanine pentapeptides[S1,S2] and the calibration shifts[S3] obtained are also shown.

Residue	Exp. pK_a	pK shift	pK^{mod}
CTr	3.67	-0.49	4.16
Asp	3.94	0.44	3.50
Glu	4.25	0.06	4.19
His	6.54	0.37	6.17
NTr	8.00	0.22	7.78
Cys	8.55	-1.00	9.55
Tyr	9.84	-0.15	9.99
Lys	10.40	-0.29	10.69

Table S6: Prevalence of the $C_\beta - C_\alpha - C_\alpha - C_\beta$ pseudo dihedral regions (defined in Figure S7) for all simulated dipeptides and charge sets in GROMACS. For each dipeptide, all average values are shown, including the differences between charge sets.

**Dipeptide**	**Dihedral**	**FF**	**Prevalence (%)**
ACE-Lys-Asp-NHE	angle1	Cph-amber	48 ± 12
		default-amber	46 ± 9
	angle2	Cph-amber	52 ± 12
		default-amber	54 ± 9
		Δ	2
ACE-Hid-Glu-NHE	angle1	Cph-amber	65 ± 9
		default-amber	60 ± 7
	angle2	Cph-amber	35 ± 9
		default-amber	40 ± 7
		Δ	5
ACE-Hie-Glu-NHE	angle1	Cph-amber	70 ± 7
		default-amber	69 ± 9
	angle2	Cph-amber	30 ± 7
		default-amber	31 ± 9
		Δ	1
ACE-Cys-Tyr-NHE	angle1	Cph-amber	63 ± 4
		default-amber	65 ± 4
	angle2	Cph-amber	37 ± 4
		default-amber	35 ± 4
		Δ	2
NAsp-CLys	angle1	Cph-amber	85 ± 4
		default-amber	88 ± 3
	angle2	Cph-amber	15 ± 4
		default-amber	12 ± 3
		Δ	3
NCys-CTyr	angle1	Cph-amber	87 ± 1
		default-amber	87 ± 2
	angle2	Cph-amber	13 ± 1
		default-amber	13 ± 2
		Δ	0

NGlu-CHid	angle1	Cph-amber	83 ± 2
		default-amber	78 ± 7
	angle2	Cph-amber	17 ± 2
		default-amber	22 ± 7
		Δ	5
NGlu-CHie	angle1	Cph-amber	81 ± 2
		default-amber	81 ± 5
	angle2	Cph-amber	19 ± 2
		default-amber	19 ± 5
		Δ	0
NHid-CGlu	angle1	Cph-amber	98 ± 2
		default-amber	95 ± 2
	angle2	Cph-amber	2 ± 2
		default-amber	5 ± 2
		Δ	3
NHie-CGlu	angle1	Cph-amber	90 ± 5
		default-amber	90 ± 3
	angle2	Cph-amber	10 ± 5
		default-amber	10 ± 3
		Δ	0
NLys-CAsp	angle1	Cph-amber	93 ± 1
		default-amber	93 ± 2
	angle2	Cph-amber	7 ± 1
		default-amber	7 ± 2
		Δ	0
NTyr-CCys	angle1	Cph-amber	80 ± 6
		default-amber	83 ± 4
	angle2	Cph-amber	20 ± 6
		default-amber	17 ± 4
		Δ	3
ACE-ALA-CALA	angle1	Cph-amber	71 ± 1
		default-amber	71 ± 3
	angle2	Cph-amber	29 ± 1
		default-amber	29 ± 3
		Δ	0

NALA-ALA-NHE	angle1	Cph-amber	96 ± 2
		default-amber	95 ± 3
	angle2	Cph-amber	4 ± 2
		default-amber	5 ± 3
		Δ	1
		Average Δ	2

Table S7: Prevalence of the $C_\beta - C_\alpha - C_\alpha - C_\beta$ pseudo dihedral regions (defined in Figure S7) for all simulated dipeptides and charge sets in AMBER. For each dipeptide, all average values are shown, including the differences between charge sets.

**Dipeptide**	**Dihedral**	**FF**	**Prevalence (%)**
ACE-Lys-Asp-NHE	angle1	Cph-amber	49 ± 9
		default-amber	52 ± 6
	angle2	Cph-amber	51 ± 9
		default-amber	48 ± 6
		Δ	3
ACE-Hid-Glu-NHE	angle1	Cph-amber	71 ± 12
		default-amber	70 ± 8
	angle2	Cph-amber	29 ± 12
		default-amber	30 ± 8
		Δ	1
ACE-Hie-Glu-NHE	angle1	Cph-amber	70 ± 10
		default-amber	66 ± 9
	angle2	Cph-amber	30 ± 10
		default-amber	34 ± 9
		Δ	4
ACE-Cys-Tyr-NHE	angle1	Cph-amber	65 ± 3
		default-amber	64 ± 5
	angle2	Cph-amber	35 ± 3
		default-amber	36 ± 5
		Δ	1

NAsp-CLys	angle1	Cph-amber	82 ± 7
		default-amber	80 ± 6
	angle2	Cph-amber	18 ± 7
		default-amber	20 ± 6
		Δ	2
NCys-CTyr	angle1	Cph-amber	87 ± 3
		default-amber	84 ± 4
	angle2	Cph-amber	13 ± 3
		default-amber	16 ± 4
		Δ	3
NGlu-CHid	angle1	Cph-amber	80 ± 12
		default-amber	89 ± 11
	angle2	Cph-amber	20 ± 12
		default-amber	11 ± 11
		Δ	9
NGlu-CHie	angle1	Cph-amber	76 ± 8
		default-amber	84 ± 7
	angle2	Cph-amber	24 ± 8
		default-amber	16 ± 7
		Δ	8
NHid-CGlu	angle1	Cph-amber	92 ± 9
		default-amber	93 ± 7
	angle2	Cph-amber	8 ± 9
		default-amber	7 ± 7
		Δ	1
NHie-CGlu	angle1	Cph-amber	90 ± 2
		default-amber	85 ± 4
	angle2	Cph-amber	10 ± 2
		default-amber	15 ± 4
		Δ	5
NLys-CAsp	angle1	Cph-amber	93 ± 4
		default-amber	87 ± 6
	angle2	Cph-amber	7 ± 4
		default-amber	13 ± 6
		Δ	6

NTyr-CCys	angle1	Cph-amber	82 ± 9
		default-amber	86 ± 7
	angle2	Cph-amber	18 ± 9
		default-amber	14 ± 7
		Δ	4
ACE-ALA-CALA	angle1	Cph-amber	74 ± 2
		default-amber	67 ± 5
	angle2	Cph-amber	26 ± 2
		default-amber	33 ± 5
		Δ	7
NALA-ALA-NHE	angle1	Cph-amber	95 ± 3
		default-amber	94 ± 3
	angle2	Cph-amber	4 ± 3
		default-amber	5 ± 3
		Δ	1
		Average Δ	4

Table S8: Isoelectric points calculated with the AMBER14SB st-CpHMD simulations. Experimental values,[S4,S5] and those calculated using CHARMM and GROMOS[S6] are also shown for comparison.

System	exp. pI	AMBER	CHARMM	GROMOS
HEWL	∼11.0[S4]	10.4	>12.0[S6]	11.4[S6]
SNase	9.6[S5]	10.1	10.1[S6]	10.0[S6]

Table S9: HEWL experimental pK_a values (taken from PKAD[S7]) and pK_a predictions from CpHMD simulations (equilibrated segment) with AMBER14SB, GROMOS54A7 and CHARMM36m. The differences (Diff) between the estimated and experimental pK_a's are also shown.

Residue	pK_a^{Exp}	AMBER		GROMOS		CHARMM	
		pK_a	Diff	pK_a	Diff	pK_a	Diff
NTr-1	7.90	8.13 ± 0.03	0.23	6.67 ± 0.08	-1.23	5.96 ± 0.09	-1.94
Lys-1	10.90	9.39 ± 0.02	-1.51	10.50 ± 0.04	-0.40	10.18 ± 0.01	-0.72
Glu-7	2.85	3.19 ± 0.01	0.34	3.55 ± 0.04	0.70	3.19 ± 0.04	0.34
Lys-13	10.60	10.23 ± 0.01	-0.37	10.76 ± 0.06	0.16	9.96 ± 0.06	-0.64
His-15	5.36	4.99 ± 0.05	-0.37	5.67 ± 0.07	0.31	4.61 ± 0.07	-0.75
Asp-18	2.66	2.79 ± 0.04	0.13	3.99 ± 0.07	1.33	3.18 ± 0.07	0.52
Tyr-20	10.30	11.67 ± 0.03	1.37	10.04 ± 0.25	-0.26	>12.0	—
Tyr-23	9.80	11.63 ± 0.16	1.83	11.16 ± 0.28	1.36	>12.0	—
Lys-33	10.60	8.83 ± 0.09	-1.77	9.28 ± 0.04	-1.32	9.09 ± 0.11	-1.51
Glu-35	6.20	5.07 ± 0.07	-1.13	6.02 ± 0.17	-0.18	5.99 ± 0.32	-0.21
Asp-48	1.60	2.70 ± 0.02	1.10	2.35 ± 0.20	0.75	3.04 ± 0.27	1.44
Asp-52	3.68	3.64 ± 0.03	-0.04	4.97 ± 0.23	1.29	4.99 ± 0.09	1.31
Tyr-53	12.10	>12.0	—	11.44 ± 0.06	-0.66	>12.0	—
Asp-66	0.90	2.75 ± 0.06	1.85	3.92 ± 0.15	3.02	2.38 ± 0.21	1.48
Asp-87	2.07	2.51 ± 0.01	0.44	2.60 ± 0.05	0.53	2.42 ± 0.04	0.35
Lys-96	10.80	9.07 ± 0.02	-1.73	10.47 ± 0.14	-0.33	9.23 ± 0.03	-1.57
Lys-97	10.30	9.90 ± 0.01	-0.40	10.33 ± 0.04	0.03	10.11 ± 0.02	-0.19
Asp-101	4.09	4.28 ± 0.02	0.19	3.87 ± 0.20	-0.22	4.11 ± 0.09	0.02
Lys-116	10.40	10.03 ± 0.01	-0.37	10.35 ± 0.07	-0.05	9.83 ± 0.02	-0.57
Asp-119	3.20	3.46 ± 0.06	0.26	3.31 ± 0.17	0.11	2.77 ± 0.05	-0.43
CTr-129	2.75	2.60 ± 0.02	-0.15	3.41 ± 0.05	0.66	3.38 ± 0.03	0.63

Table S10: SNase experimental pK_a values (taken from PKAD[S7]) and pK_a predictions from CpHMD simulations (equilibrated segment) with AMBER14SB, GROMOS54A7 and CHARMM36m. The differences (Diff) between the estimated and experimental pK_a's are also shown.

Residue	pK_a^{Exp}	AMBER		GROMOS		CHARMM	
		pK_a	Diff	pK_a	Diff	pK_a	Diff
His-8	6.52	5.92 ± 0.04	-0.60	5.72 ± 0.05	-0.80	5.97 ± 0.03	-0.55
Glu-10	2.82	2.91 ± 0.02	0.09	4.06 ± 0.22	1.24	3.22 ± 0.05	0.40
Asp-40	3.87	3.29 ± 0.02	-0.58	4.12 ± 0.02	0.25	3.49 ± 0.10	-0.38
Glu-43	4.32	3.34 ± 0.04	-0.98	4.07 ± 0.09	-0.25	4.29 ± 0.11	-0.03
His-46	5.86	-0.37 ± 3.03	-6.23	3.97 ± 1.00	-1.89	2.09 ± 0.64	-3.77
Glu-52	3.93	4.31 ± 0.04	0.38	5.28 ± 0.11	1.35	5.04 ± 0.25	1.11
Glu-57	3.49	3.79 ± 0.02	0.30	4.37 ± 0.08	0.88	4.29 ± 0.10	0.80
Glu-67	3.76	3.86 ± 0.02	0.10	4.72 ± 0.02	0.96	3.74 ± 0.05	-0.02
Glu-73	3.31	3.23 ± 0.01	-0.08	4.79 ± 0.02	1.49	3.48 ± 0.08	0.17
Glu-75	3.26	4.34 ± 0.13	1.08	6.32 ± 0.15	3.06	5.41 ± 0.20	2.15
Asp-95	2.16	4.23 ± 0.01	2.07	2.95 ± 0.23	0.79	3.08 ± 0.03	0.92
Glu-101	3.81	2.58 ± 0.11	-1.23	3.06 ± 0.17	-0.74	3.69 ± 0.13	-0.12
His-121	5.30	3.24 ± 0.12	-2.06	3.40 ± 0.17	-1.90	1.00 ± 1.35	-4.30
Glu-122	3.89	3.92 ± 0.09	0.03	5.06 ± 0.20	1.17	4.38 ± 0.14	0.49
His-124	5.73	4.87 ± 0.04	-0.86	4.70 ± 0.03	-1.03	4.80 ± 0.21	-0.93
Glu-129	3.75	3.43 ± 0.03	-0.32	4.79 ± 0.08	1.04	3.71 ± 0.06	-0.04
Glu-135	3.76	3.30 ± 0.06	-0.46	4.44 ± 0.00	0.68	3.47 ± 0.06	-0.29

Table S11: RMSE values for each titrating residue type. The mean error (ME) values can be seen as a measure of the bias and are shown inside parentheses.

Residue	Exp residues	RMSE (ME)
CTr	1	0.15 (-0.15)
Asp	9	0.93 (+0.49)
Glu	13	0.64 (-0.17)
His	5	2.98 (-2.02)
NTr	1	0.23 (+0.23)
Cys	0	No data
Tyr	3	1.72 (+1.70)
Lys	6	1.21 (-1.03)

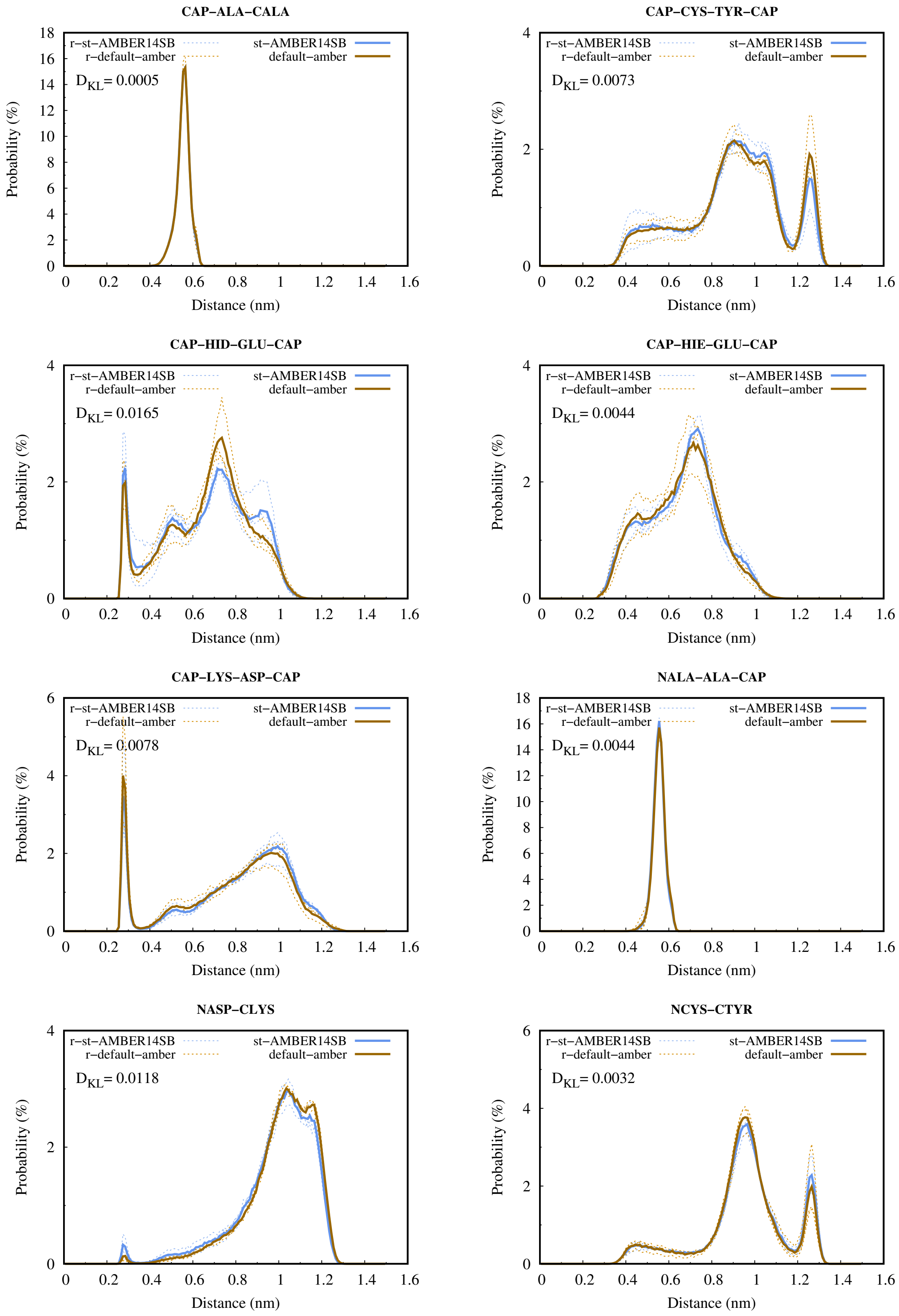
CAP–ALA–CALA
r–st–AMBER14SB
r–default–amber
st–AMBER14SB
default–amber
D_KL= 0.0005
CAP–CYS–TYR–CAP
D_KL= 0.0073
CAP–HID–GLU–CAP
D_KL= 0.0165
CAP–HIE–GLU–CAP
D_KL= 0.0044
CAP–LYS–ASP–CAP
D_KL= 0.0078
NALA–ALA–CAP
D_KL= 0.0044
NASP–CLYS
D_KL= 0.0118
NCYS–CTYR
D_KL= 0.0032
Probability (%)
Distance (nm)

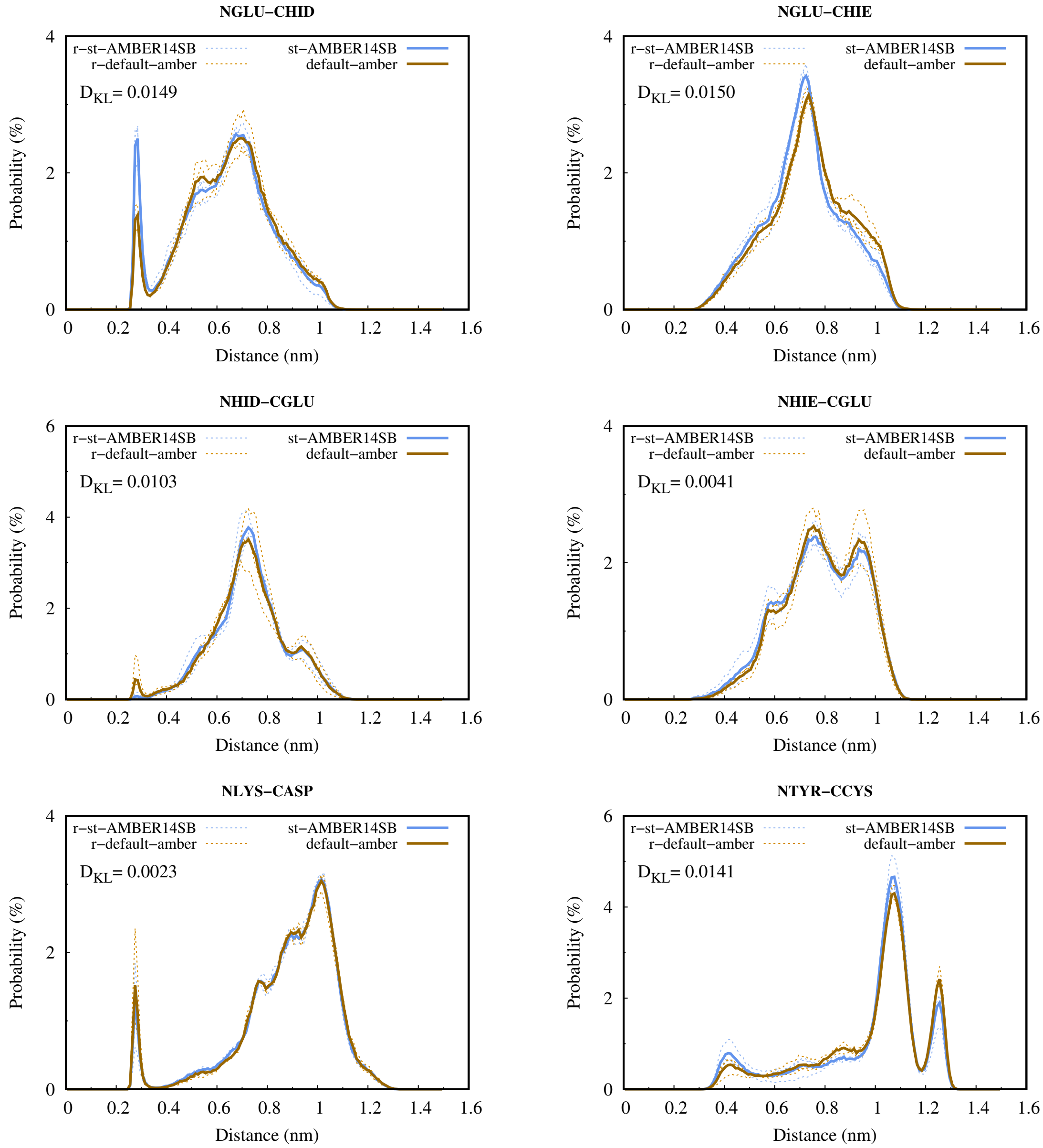

Figure S1: Side chain distance histograms in GROMACS simulated dipeptides. The simulations with the changed (st-AMBER14SB, in blue) and with the default charge sets (default-amber, in brown) are shown. The solid lines represent the averages between all replicates (dotted lines). The Kullback-Leibler divergence values[S8] are shown for each distribution.

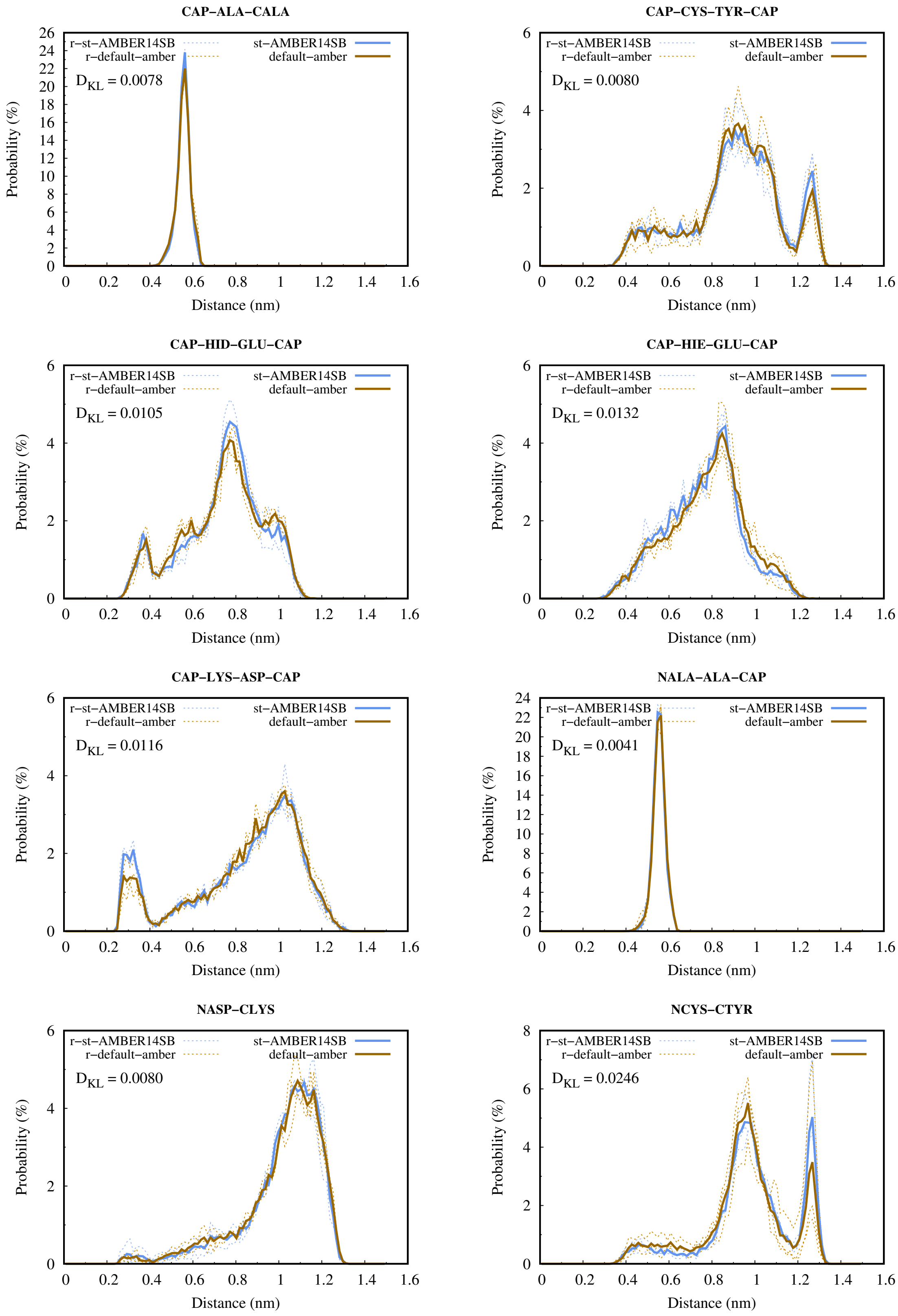

CAP–ALA–CALA
r–st–AMBER14SB
r–default–amber
st–AMBER14SB
default–amber
D_KL = 0.0078
Probability (%)
Distance (nm)
CAP–CYS–TYR–CAP
D_KL = 0.0080
CAP–HID–GLU–CAP
D_KL = 0.0105
CAP–HIE–GLU–CAP
D_KL = 0.0132
CAP–LYS–ASP–CAP
D_KL = 0.0116
NALA–ALA–CAP
D_KL = 0.0041
NASP–CLYS
D_KL = 0.0080
NCYS–CTYR
D_KL = 0.0246

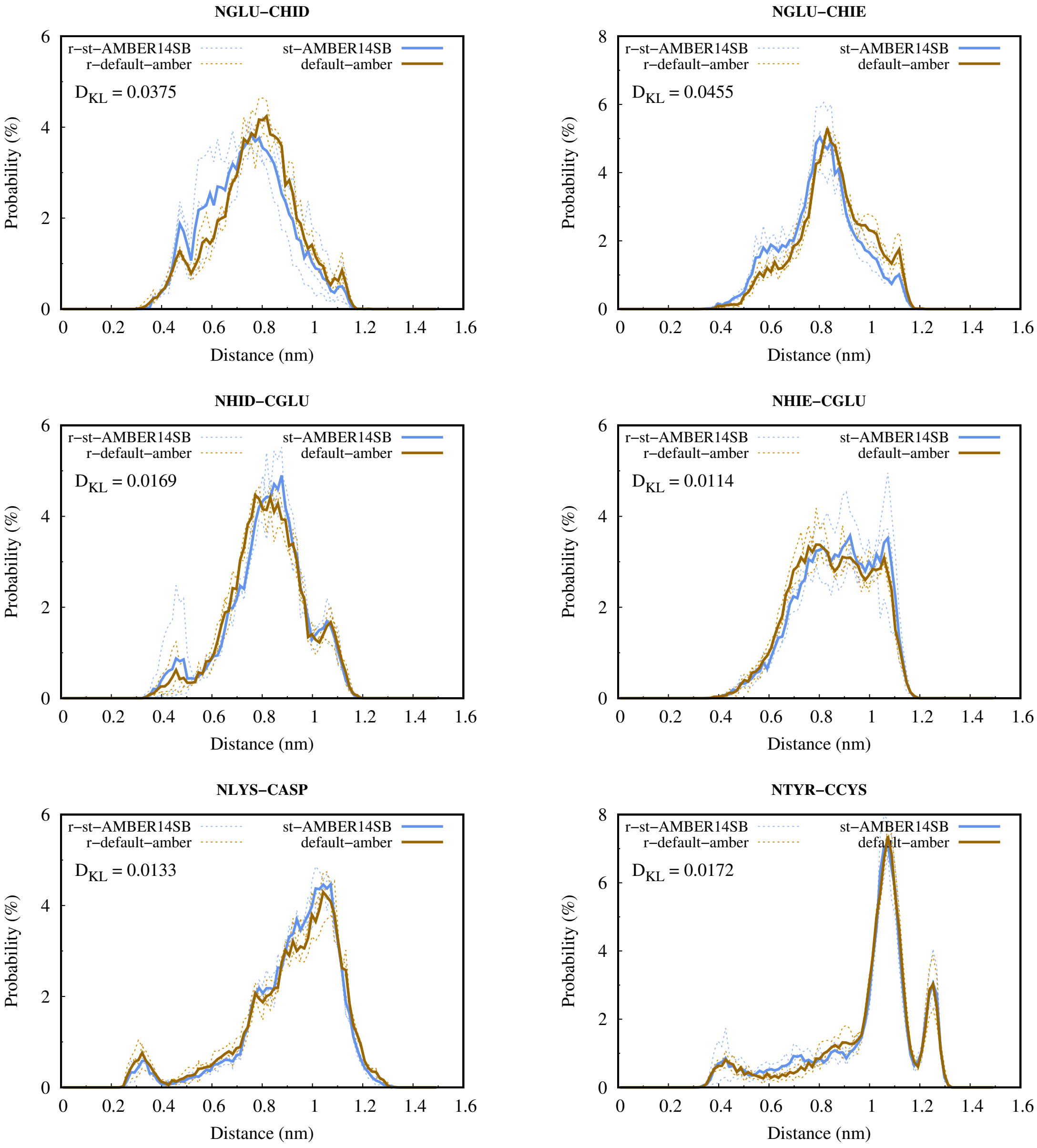

Figure S2: Side chain distance histograms of the AMBER simulated dipeptides. The simulations with the changed (st-AMBER14SB, in blue) and with the default charge sets (default-amber, in brown) are shown. The solid lines represent the averages between all replicates (dotted lines). The Kullback-Leibler divergence values[S8] are shown for each distribution.

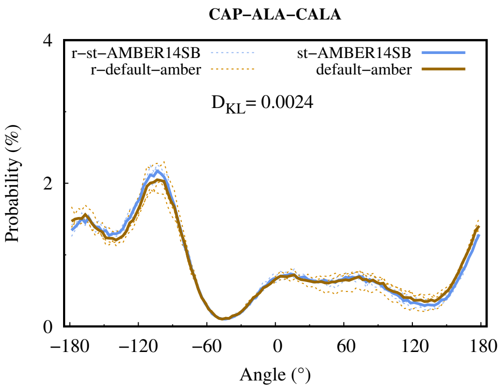
CAP–ALA–CALA
r–st–AMBER14SB
r–default–amber
st–AMBER14SB
default–amber
D_{KL}= 0.0024
Probability (%)
Angle (°)

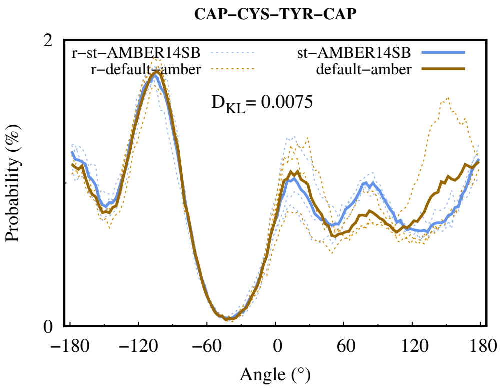
CAP–CYS–TYR–CAP
r–st–AMBER14SB
r–default–amber
st–AMBER14SB
default–amber
D_{KL}= 0.0075
Probability (%)
Angle (°)

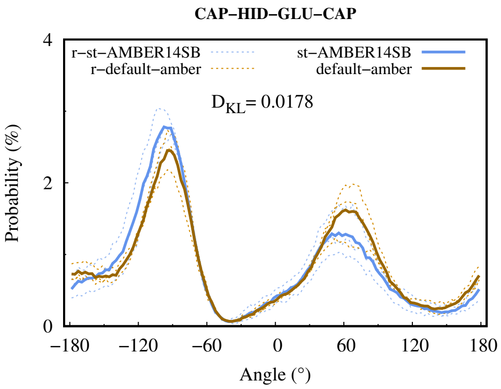
CAP–HID–GLU–CAP
r–st–AMBER14SB
r–default–amber
st–AMBER14SB
default–amber
D_{KL}= 0.0178
Probability (%)
Angle (°)

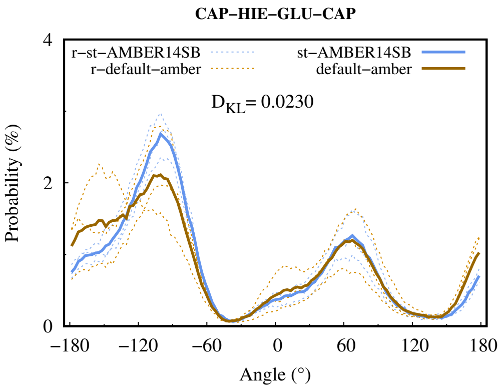
CAP–HIE–GLU–CAP
r–st–AMBER14SB
r–default–amber
st–AMBER14SB
default–amber
D_{KL}= 0.0230
Probability (%)
Angle (°)

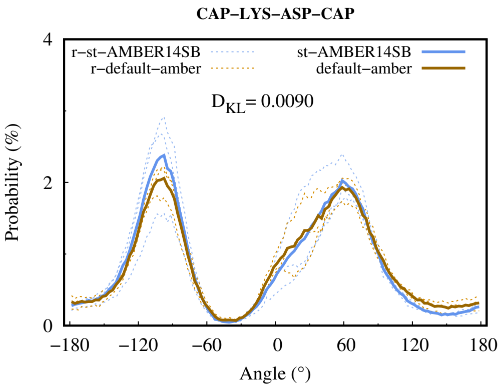
CAP–LYS–ASP–CAP
r–st–AMBER14SB
r–default–amber
st–AMBER14SB
default–amber
D_{KL}= 0.0090
Probability (%)
Angle (°)

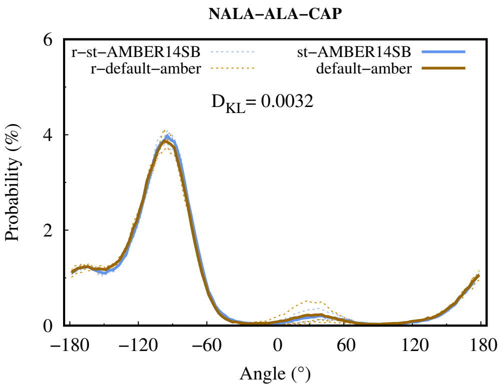
NALA–ALA–CAP
r–st–AMBER14SB
r–default–amber
st–AMBER14SB
default–amber
D_{KL}= 0.0032
Probability (%)
Angle (°)

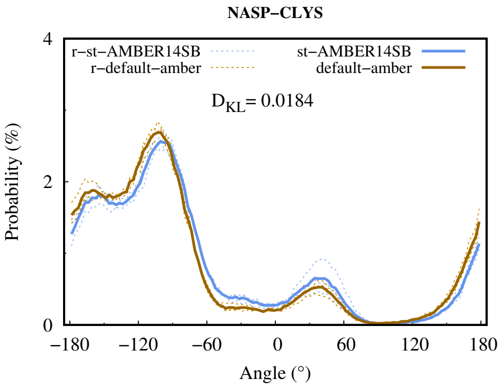
NASP–CLYS
r–st–AMBER14SB
r–default–amber
st–AMBER14SB
default–amber
D_{KL}= 0.0184
Probability (%)
Angle (°)

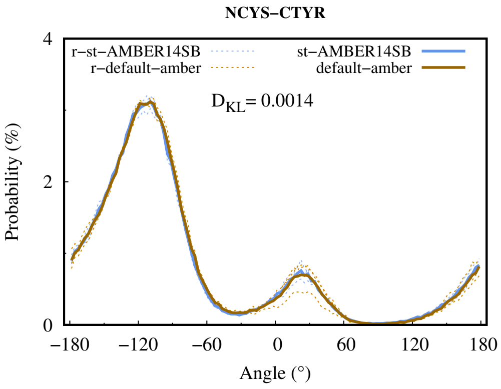
NCYS–CTYR
r–st–AMBER14SB
r–default–amber
st–AMBER14SB
default–amber
D_{KL}= 0.0014
Probability (%)
Angle (°)

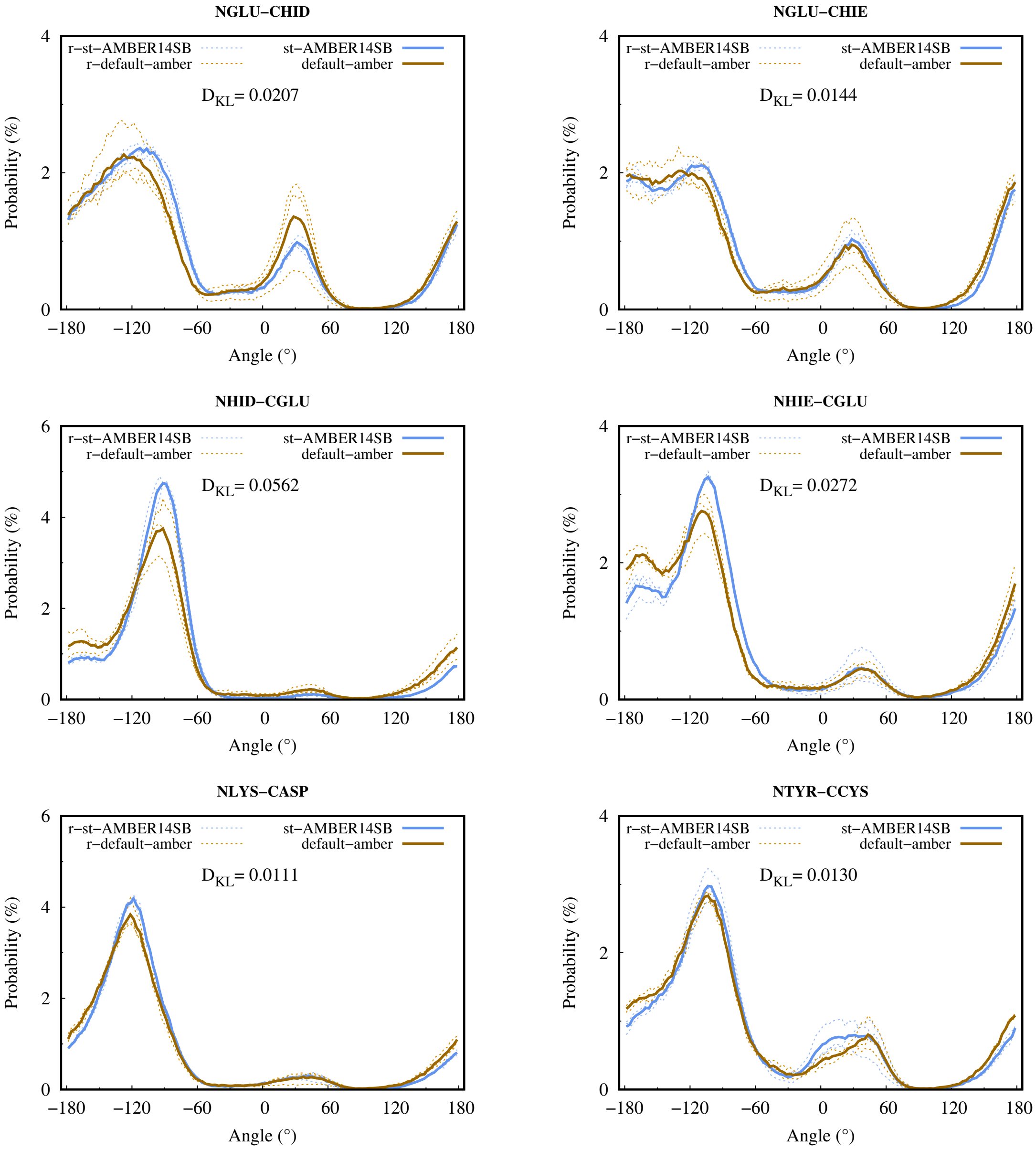

Figure S3: Histogram of the Cβ – Cα pseudo dihedral in GROMACS simulated dipeptides. The simulations with the changed (st-AMBER14SB, in blue) and with the default charge sets (default-amber, in brown) are shown. The solid lines represent the averages between all replicates (dotted lines). The Kullback-Leibler divergence values[S8] are shown for each distribution.

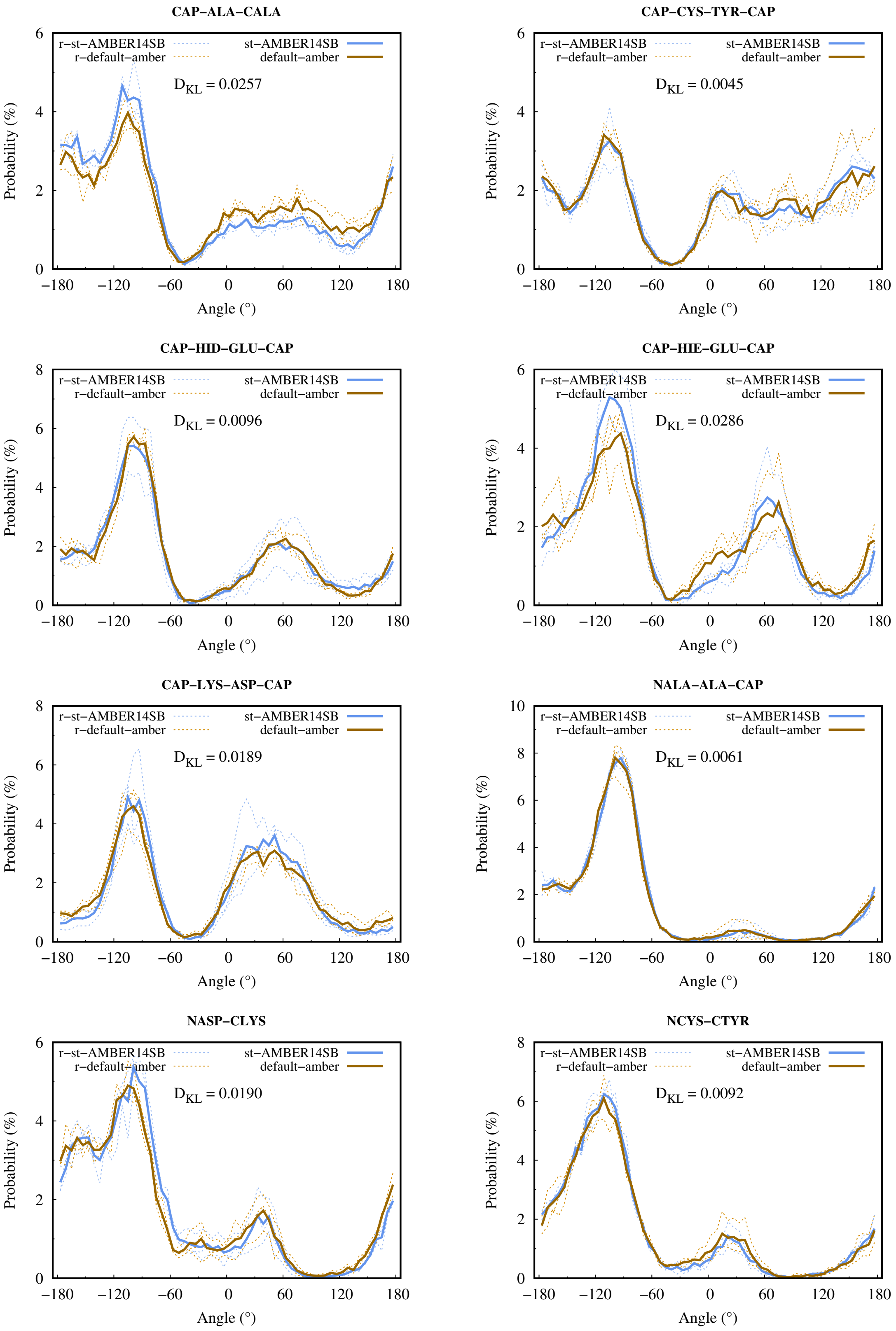
CAP−ALA−CALA
D_KL = 0.0257
CAP−CYS−TYR−CAP
D_KL = 0.0045
CAP−HID−GLU−CAP
D_KL = 0.0096
CAP−HIE−GLU−CAP
D_KL = 0.0286
CAP−LYS−ASP−CAP
D_KL = 0.0189
NALA−ALA−CAP
D_KL = 0.0061
NASP−CLYS
D_KL = 0.0190
NCYS−CTYR
D_KL = 0.0092
r−st−AMBER14SB
r−default−amber
st−AMBER14SB
default−amber
Probability (%)
Angle (°)

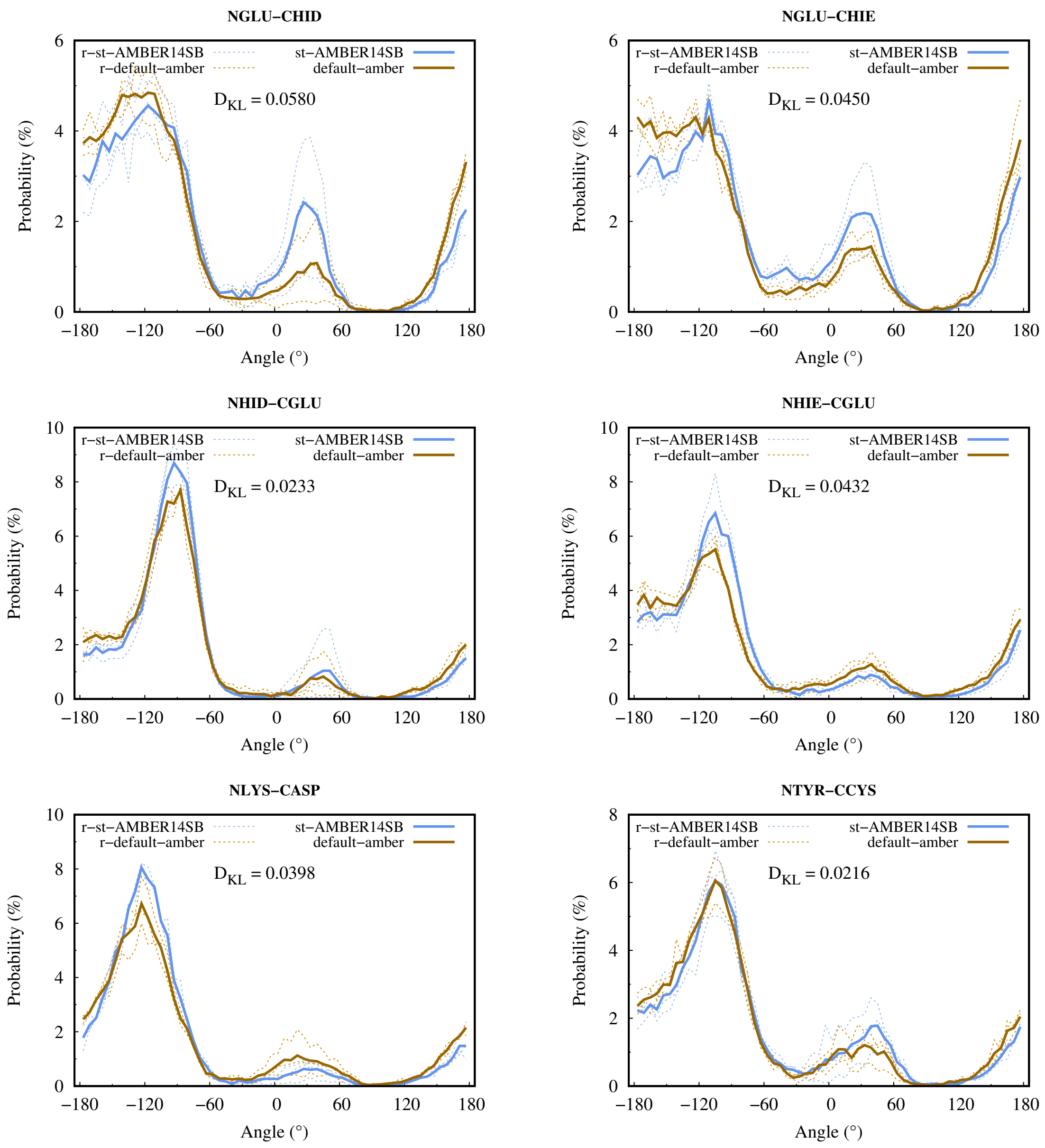

Figure S4: Histogram of the $C\beta - C\alpha$ pseudo dihedral in AMBER simulated dipeptides. The simulations with the changed (st-AMBER14SB, in blue) and with the default charge sets (default-amber, in brown) are shown. The solid lines represent the averages between all replicates (dotted lines). The Kullback-Leibler divergence values[S8] are shown for each distribution.

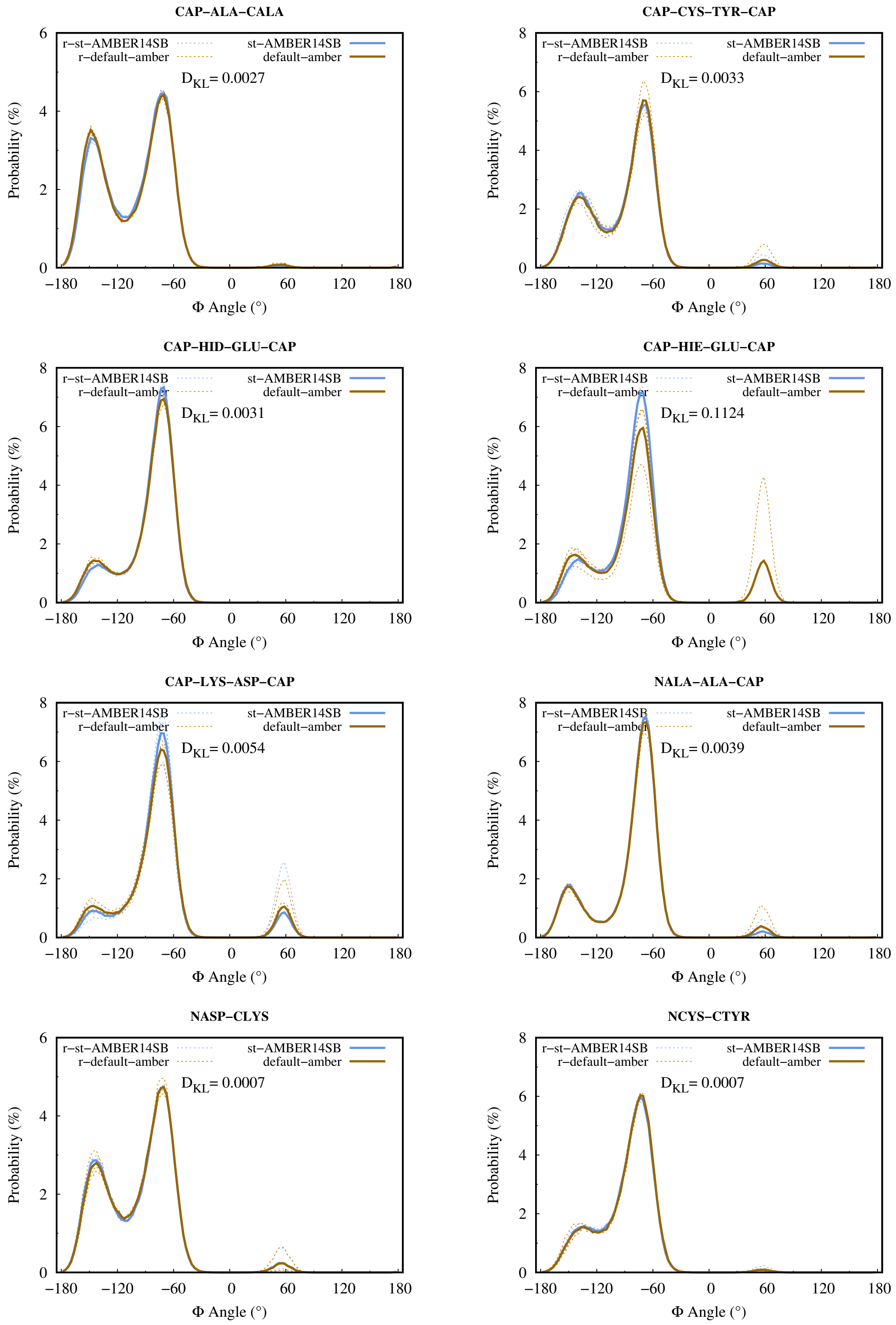
CAP–ALA–CALA
r–st–AMBER14SB
r–default–amber
st–AMBER14SB
default–amber
D_KL= 0.0027
CAP–CYS–TYR–CAP
D_KL= 0.0033
CAP–HID–GLU–CAP
D_KL= 0.0031
CAP–HIE–GLU–CAP
D_KL= 0.1124
CAP–LYS–ASP–CAP
D_KL= 0.0054
NALA–ALA–CAP
D_KL= 0.0039
NASP–CLYS
D_KL= 0.0007
NCYS–CTYR
D_KL= 0.0007
Probability (%)
Φ Angle (°)

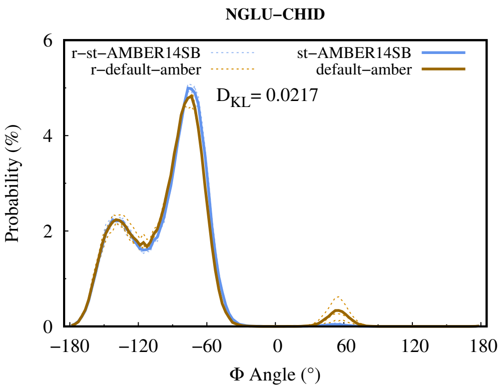
NGLU–CHID
r–st–AMBER14SB
r–default–amber
st–AMBER14SB
default–amber
D_{KL}= 0.0217
Probability (%)
Φ Angle (°)

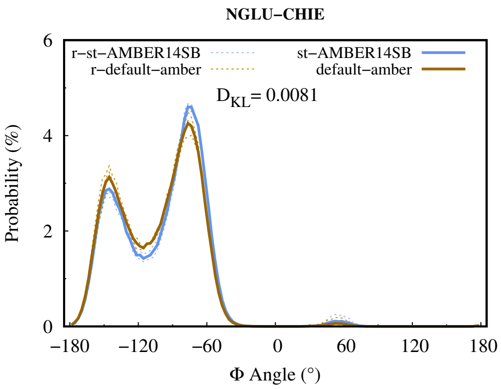
NGLU–CHIE
r–st–AMBER14SB
r–default–amber
st–AMBER14SB
default–amber
D_{KL}= 0.0081
Probability (%)
Φ Angle (°)

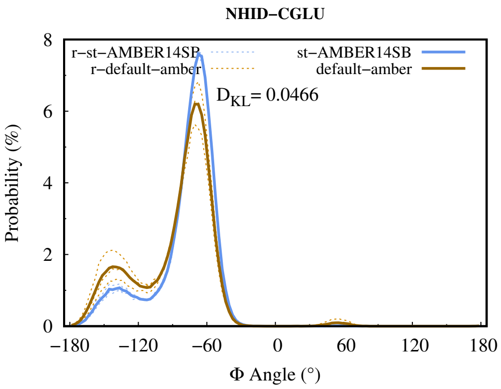
NHID–CGLU
r–st–AMBER14SB
r–default–amber
st–AMBER14SB
default–amber
D_{KL}= 0.0466
Probability (%)
Φ Angle (°)

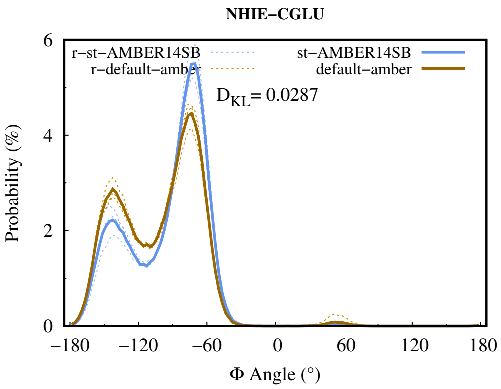
NHIE–CGLU
r–st–AMBER14SB
r–default–amber
st–AMBER14SB
default–amber
D_{KL}= 0.0287
Probability (%)
Φ Angle (°)

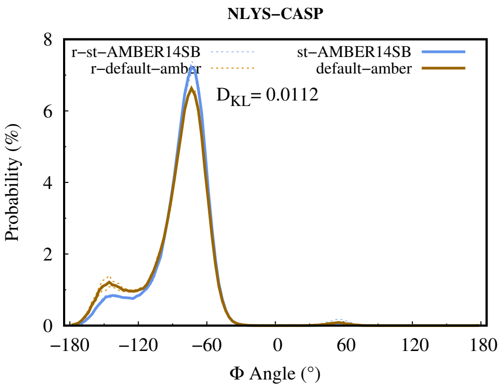
NLYS–CASP
r–st–AMBER14SB
r–default–amber
st–AMBER14SB
default–amber
D_{KL}= 0.0112
Probability (%)
Φ Angle (°)

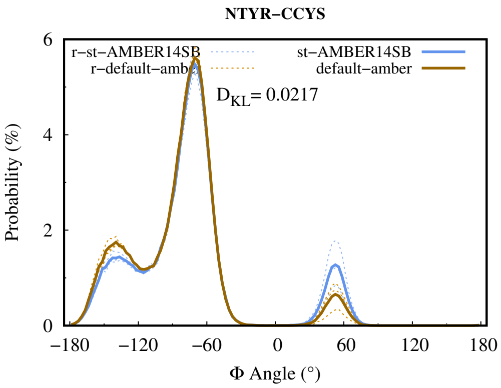
NTYR–CCYS
r–st–AMBER14SB
r–default–amber
st–AMBER14SB
default–amber
D_{KL}= 0.0217
Probability (%)
Φ Angle (°)

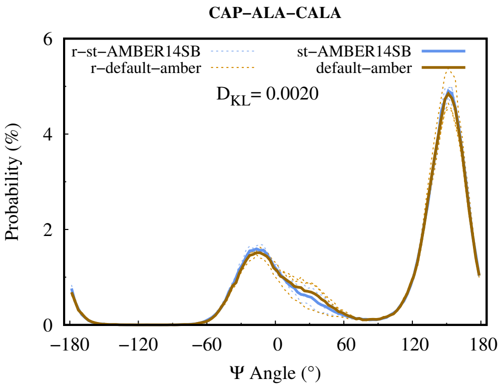
CAP–ALA–CALA
r–st–AMBER14SB
r–default–amber
st–AMBER14SB
default–amber
D_{KL}= 0.0020
Probability (%)
Ψ Angle (°)

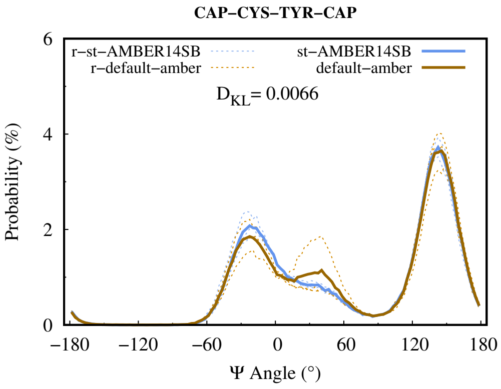
CAP–CYS–TYR–CAP
r–st–AMBER14SB
r–default–amber
st–AMBER14SB
default–amber
D_{KL}= 0.0066
Probability (%)
Ψ Angle (°)

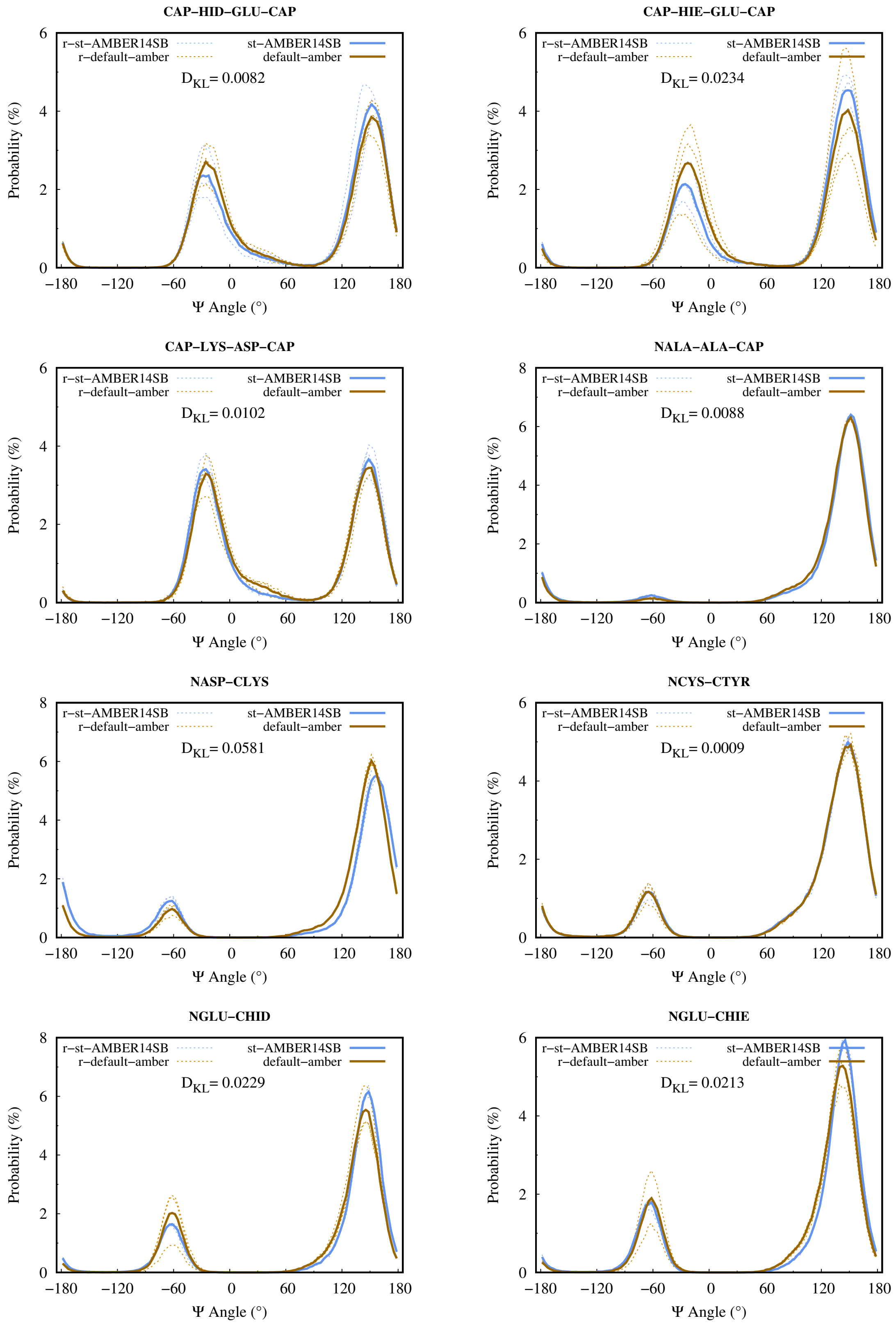
CAP–HID–GLU–CAP
r–st–AMBER14SB
r–default–amber
st–AMBER14SB
default–amber
D_{KL}= 0.0082
CAP–HIE–GLU–CAP
D_{KL}= 0.0234
CAP–LYS–ASP–CAP
D_{KL}= 0.0102
NALA–ALA–CAP
D_{KL}= 0.0088
NASP–CLYS
D_{KL}= 0.0581
NCYS–CTYR
D_{KL}= 0.0009
NGLU–CHID
D_{KL}= 0.0229
NGLU–CHIE
D_{KL}= 0.0213
Probability (%)
Ψ Angle (°)

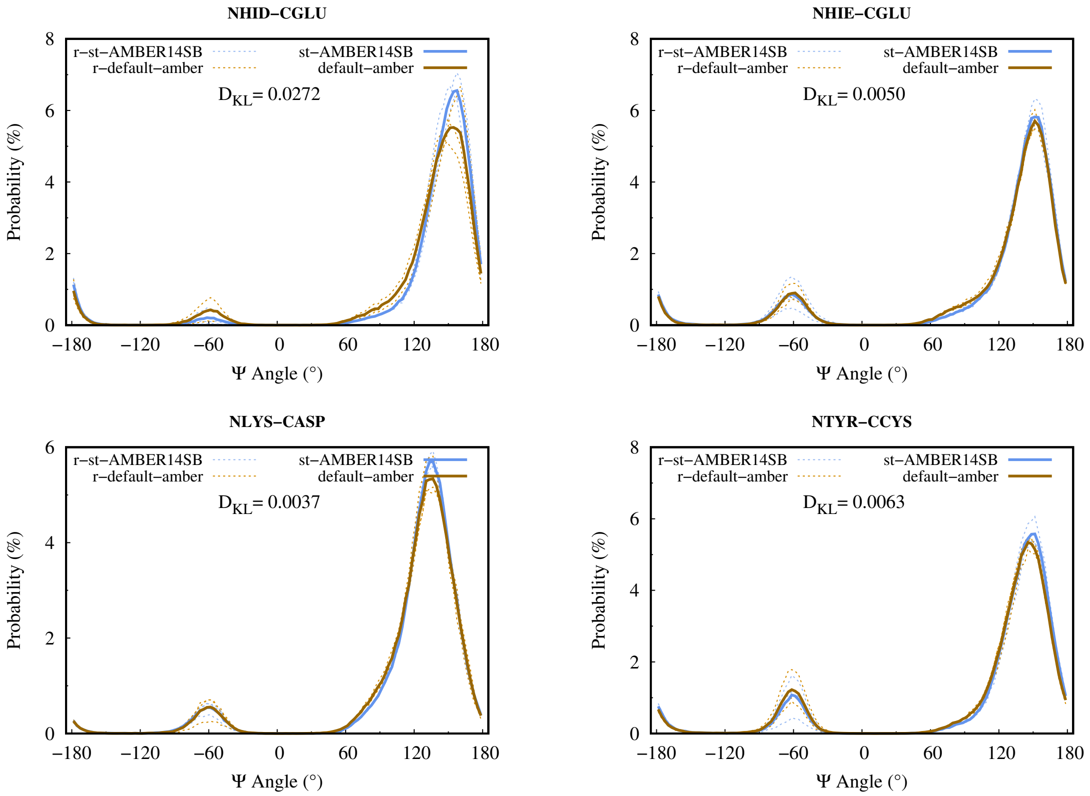

Figure S5: Histogram of the Φ and Ψ dihedrals in GROMACS simulated dipeptides. The simulations with the changed (st-AMBER14SB, in blue) and with the default charge sets (default-amber, in brown) are shown. The solid lines represent the averages between all replicates (dotted lines). The Kullback-Leibler divergence values[S8] are shown for each distribution.

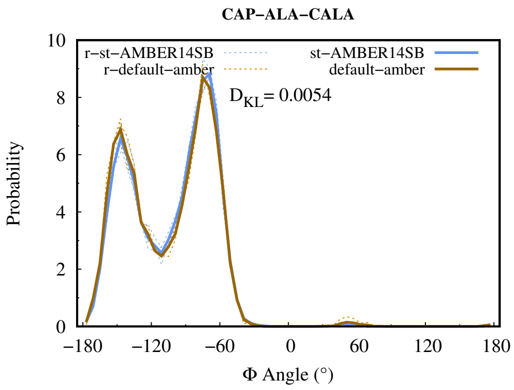
CAP−ALA−CALA
r−st−AMBER14SB
r−default−amber
st−AMBER14SB
default−amber
D_KL= 0.0054
Probability
Φ Angle (°)

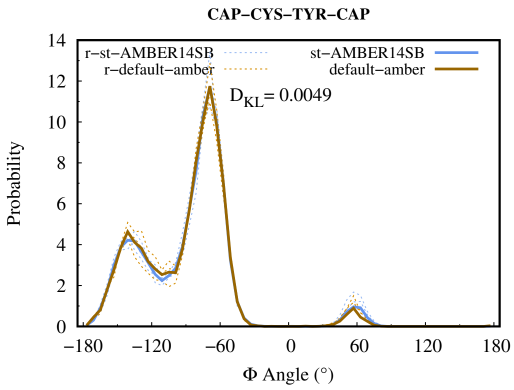
CAP−CYS−TYR−CAP
r−st−AMBER14SB
r−default−amber
st−AMBER14SB
default−amber
D_KL= 0.0049
Probability
Φ Angle (°)

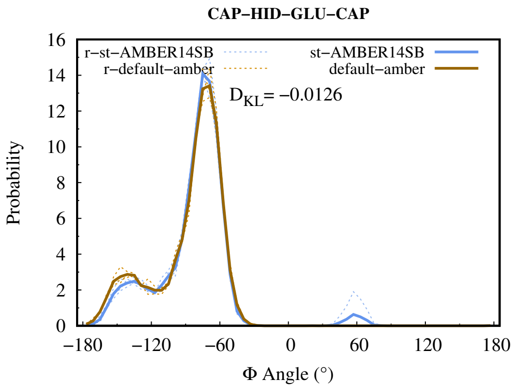
CAP−HID−GLU−CAP
r−st−AMBER14SB
r−default−amber
st−AMBER14SB
default−amber
D_KL= −0.0126
Probability
Φ Angle (°)

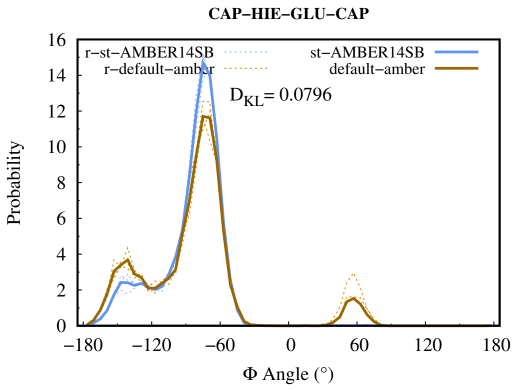
CAP−HIE−GLU−CAP
r−st−AMBER14SB
r−default−amber
st−AMBER14SB
default−amber
D_KL= 0.0796
Probability
Φ Angle (°)

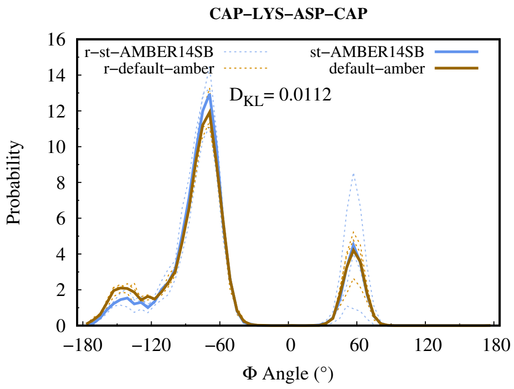
CAP−LYS−ASP−CAP
r−st−AMBER14SB
r−default−amber
st−AMBER14SB
default−amber
D_KL= 0.0112
Probability
Φ Angle (°)

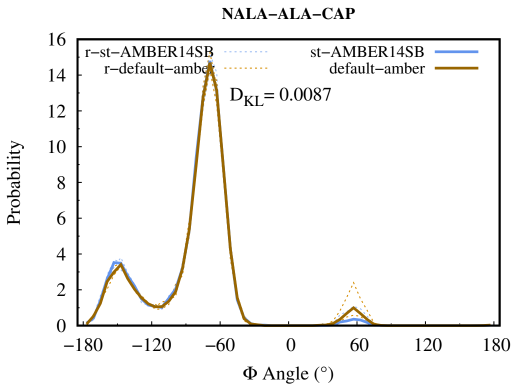
NALA−ALA−CAP
r−st−AMBER14SB
r−default−amber
st−AMBER14SB
default−amber
D_KL= 0.0087
Probability
Φ Angle (°)

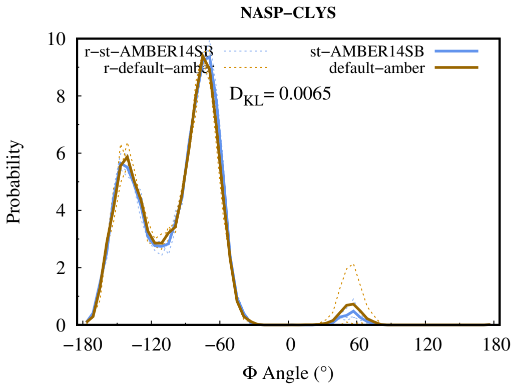
NASP−CLYS
r−st−AMBER14SB
r−default−amber
st−AMBER14SB
default−amber
D_KL= 0.0065
Probability
Φ Angle (°)

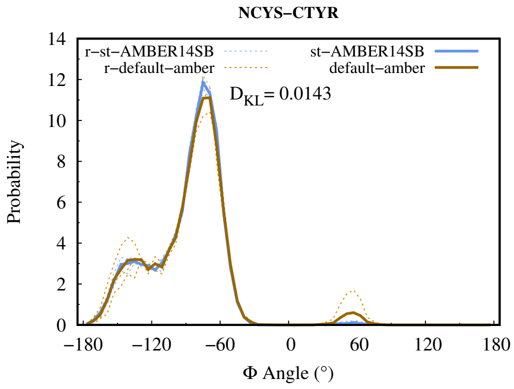
NCYS−CTYR
r−st−AMBER14SB
r−default−amber
st−AMBER14SB
default−amber
D_KL= 0.0143
Probability
Φ Angle (°)

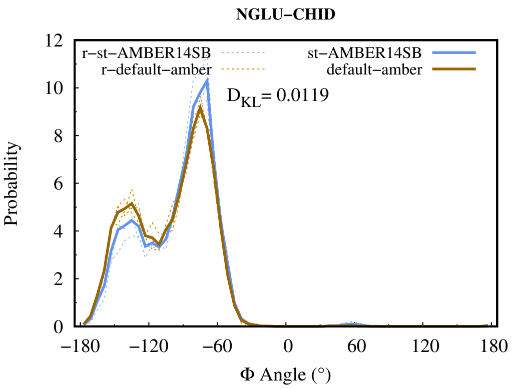
NGLU–CHID
r–st–AMBER14SB
r–default–amber
st–AMBER14SB
default–amber
D_{KL}= 0.0119
Probability
Φ Angle (°)

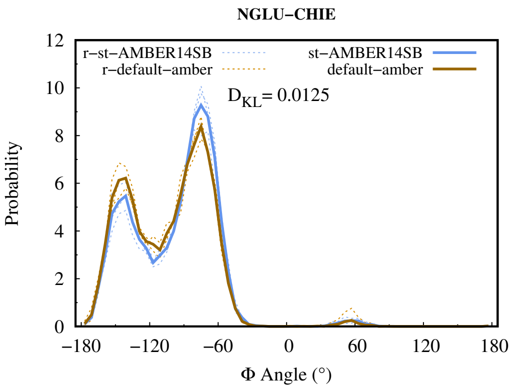
NGLU–CHIE
r–st–AMBER14SB
r–default–amber
st–AMBER14SB
default–amber
D_{KL}= 0.0125
Probability
Φ Angle (°)

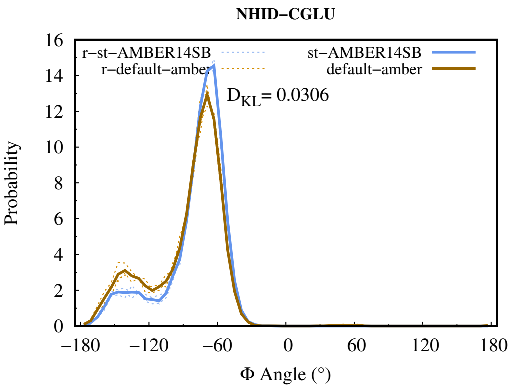
NHID–CGLU
r–st–AMBER14SB
r–default–amber
st–AMBER14SB
default–amber
D_{KL}= 0.0306
Probability
Φ Angle (°)

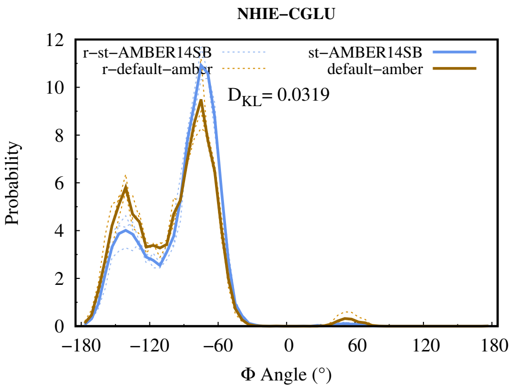
NHIE–CGLU
r–st–AMBER14SB
r–default–amber
st–AMBER14SB
default–amber
D_{KL}= 0.0319
Probability
Φ Angle (°)

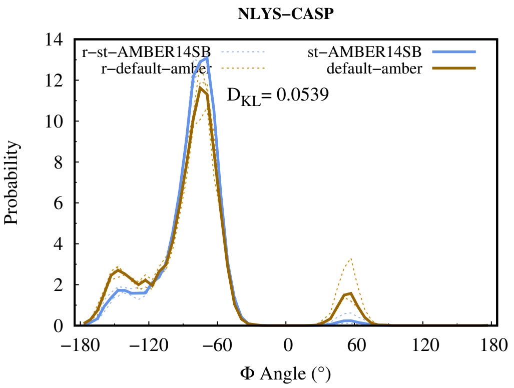
NLYS–CASP
r–st–AMBER14SB
r–default–amber
st–AMBER14SB
default–amber
D_{KL}= 0.0539
Probability
Φ Angle (°)

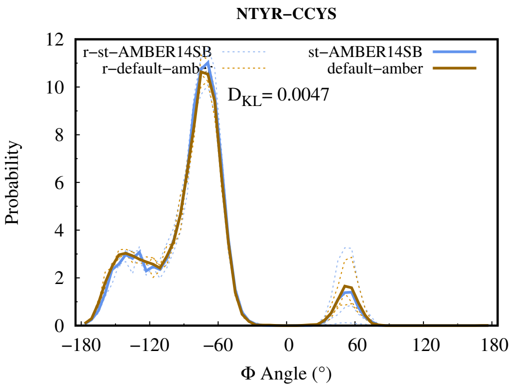
NTYR–CCYS
r–st–AMBER14SB
r–default–amber
st–AMBER14SB
default–amber
D_{KL}= 0.0047
Probability
Φ Angle (°)

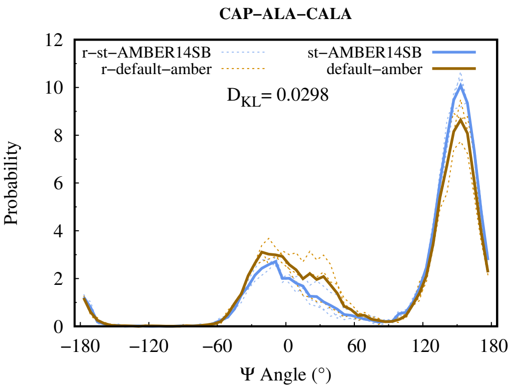
CAP–ALA–CALA
r–st–AMBER14SB
r–default–amber
st–AMBER14SB
default–amber
D_{KL}= 0.0298
Probability
Ψ Angle (°)

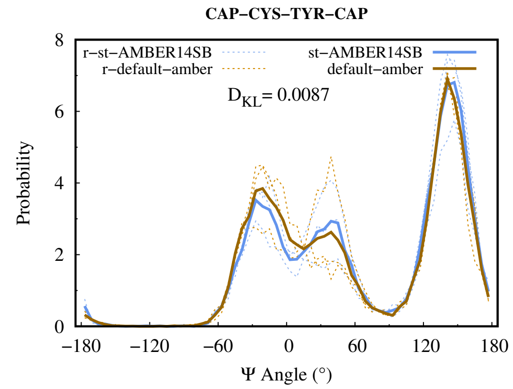
CAP–CYS–TYR–CAP
r–st–AMBER14SB
r–default–amber
st–AMBER14SB
default–amber
D_{KL}= 0.0087
Probability
Ψ Angle (°)

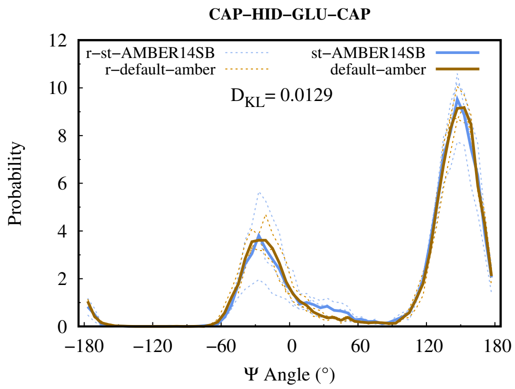
CAP–HID–GLU–CAP
r–st–AMBER14SB
r–default–amber
st–AMBER14SB
default–amber
D_{KL}= 0.0129
Probability
Ψ Angle (°)

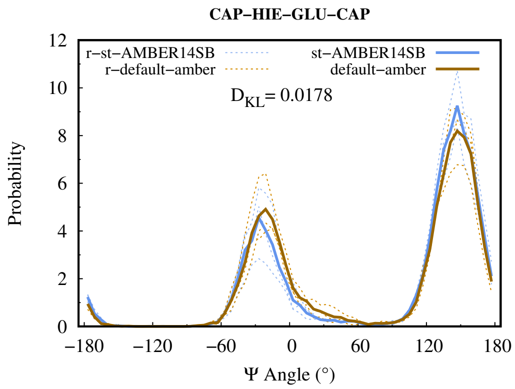
CAP–HIE–GLU–CAP
r–st–AMBER14SB
r–default–amber
st–AMBER14SB
default–amber
D_{KL}= 0.0178
Probability
Ψ Angle (°)

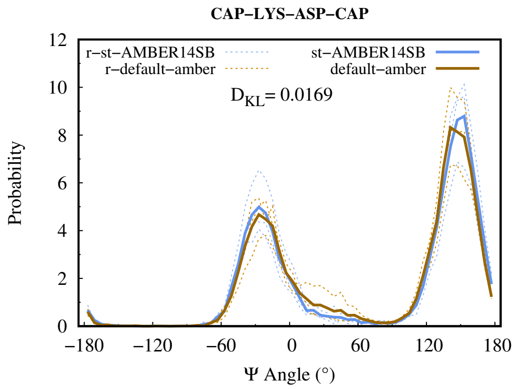
CAP–LYS–ASP–CAP
r–st–AMBER14SB
r–default–amber
st–AMBER14SB
default–amber
D_{KL}= 0.0169
Probability
Ψ Angle (°)

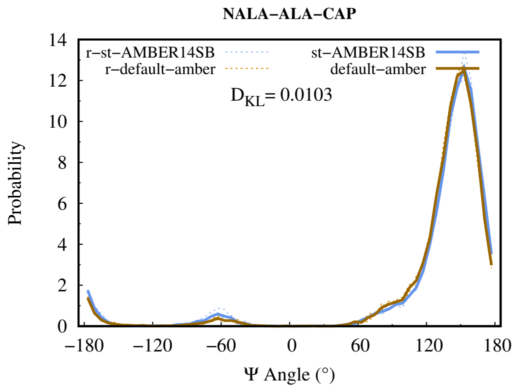
NALA–ALA–CAP
r–st–AMBER14SB
r–default–amber
st–AMBER14SB
default–amber
D_{KL}= 0.0103
Probability
Ψ Angle (°)

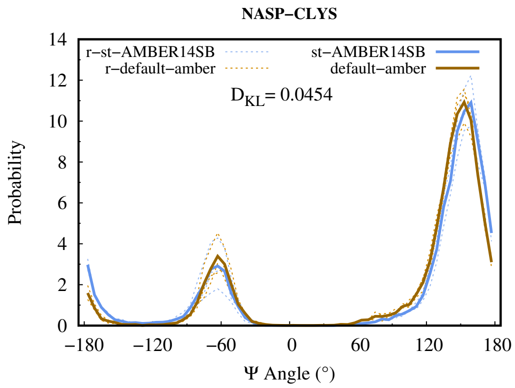
NASP–CLYS
r–st–AMBER14SB
r–default–amber
st–AMBER14SB
default–amber
D_{KL}= 0.0454
Probability
Ψ Angle (°)

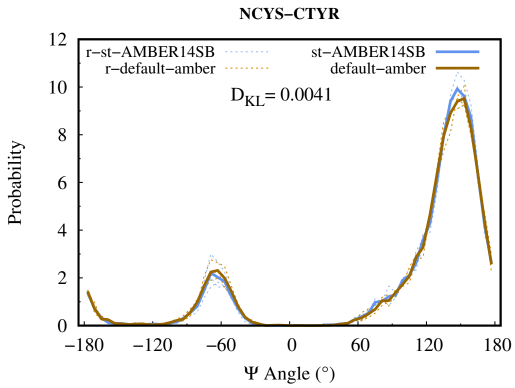
NCYS–CTYR
r–st–AMBER14SB
r–default–amber
st–AMBER14SB
default–amber
D_{KL}= 0.0041
Probability
Ψ Angle (°)

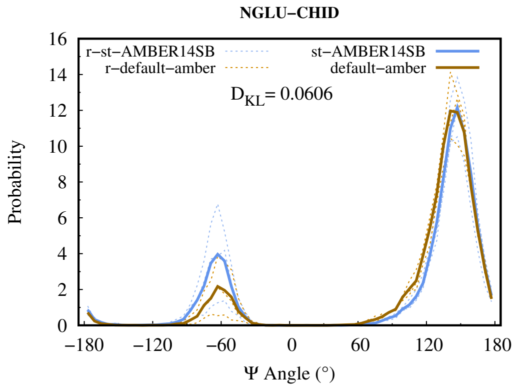
NGLU–CHID
r–st–AMBER14SB
r–default–amber
st–AMBER14SB
default–amber
D_{KL}= 0.0606
Probability
Ψ Angle (°)

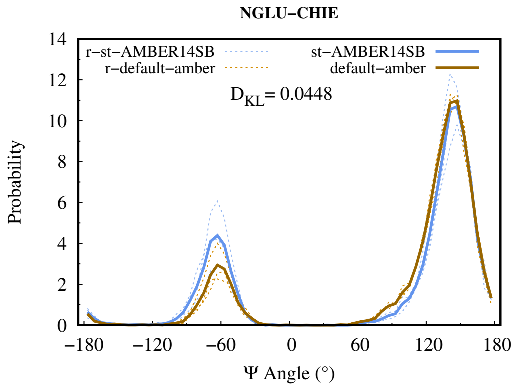
NGLU–CHIE
r–st–AMBER14SB
r–default–amber
st–AMBER14SB
default–amber
D_{KL}= 0.0448
Probability
Ψ Angle (°)

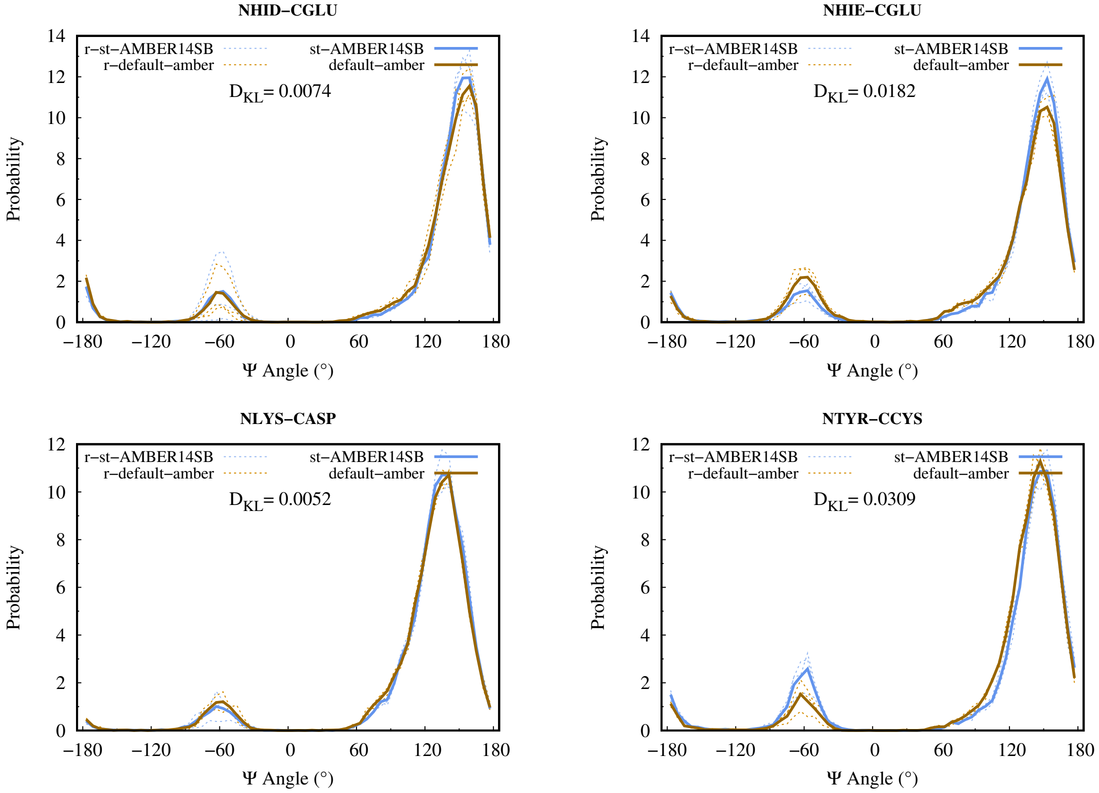

Figure S6: Histogram of the Φ and Ψ dihedrals in AMBER simulated dipeptides. The simulations with the changed (st-AMBER14SB, in blue) and with the default charge sets (default-amber, in brown) are shown. The solid lines represent the averages between all replicates (dotted lines). The Kullback-Leibler divergence values[S8] are shown for each distribution.

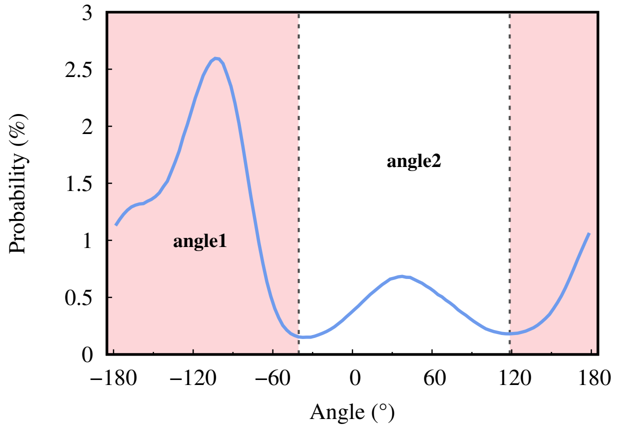

Figure S7: Histogram of the $C\beta - C\alpha - C\alpha - C\beta$ pseudo dihedral considering all GROMACS dipeptide simulations (including both charge sets). The colors depict the most populated regions of the distribution. Pink represents the "angle1" and white the "angle2".

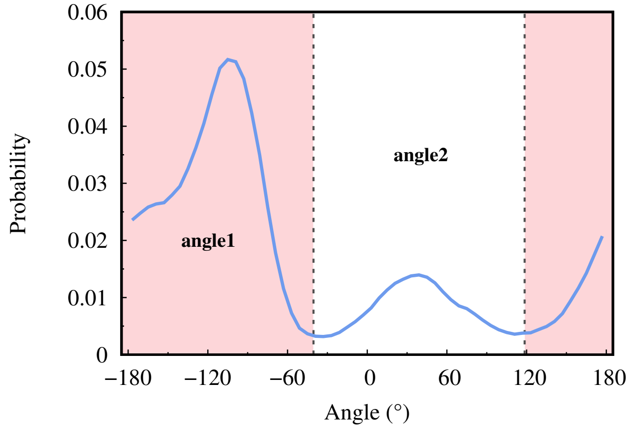

Figure S8: Histogram of the $C\beta - C\alpha - C\alpha - C\beta$ pseudo dihedral considering all AMBER dipeptide simulations (including both charge sets). The colors depict the most populated regions of the distribution. Pink represents the "angle1" and white the "angle2".

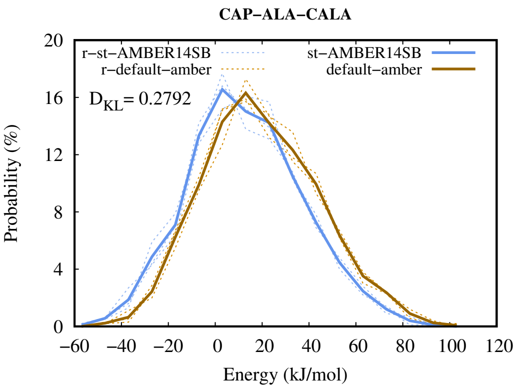
CAP–ALA–CALA
r–st–AMBER14SB
r–default–amber
st–AMBER14SB
default–amber
D$_{KL}$= 0.2792
Probability (%)
Energy (kJ/mol)

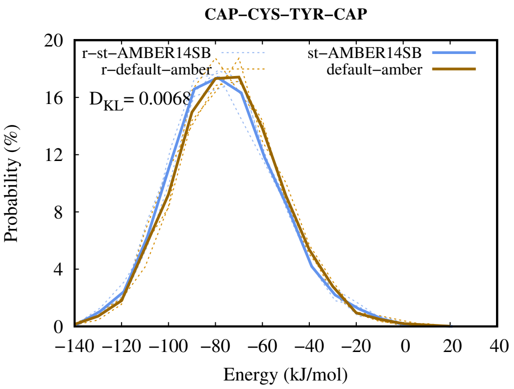
CAP–CYS–TYR–CAP
r–st–AMBER14SB
r–default–amber
st–AMBER14SB
default–amber
D$_{KL}$= 0.0068
Probability (%)
Energy (kJ/mol)

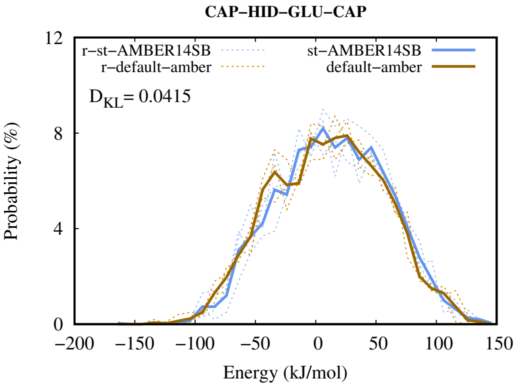
CAP–HID–GLU–CAP
r–st–AMBER14SB
r–default–amber
st–AMBER14SB
default–amber
D$_{KL}$= 0.0415
Probability (%)
Energy (kJ/mol)

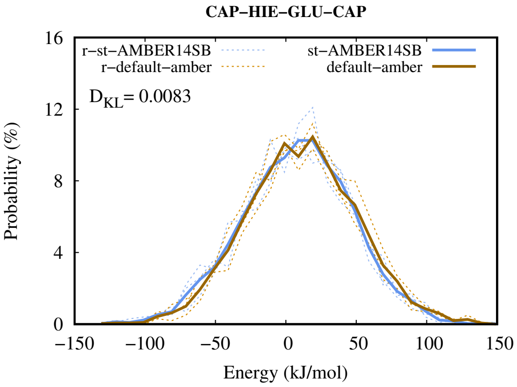
CAP–HIE–GLU–CAP
r–st–AMBER14SB
r–default–amber
st–AMBER14SB
default–amber
D$_{KL}$= 0.0083
Probability (%)
Energy (kJ/mol)

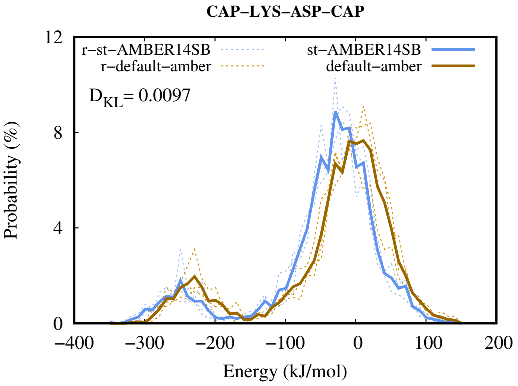
CAP–LYS–ASP–CAP
r–st–AMBER14SB
r–default–amber
st–AMBER14SB
default–amber
D$_{KL}$= 0.0097
Probability (%)
Energy (kJ/mol)

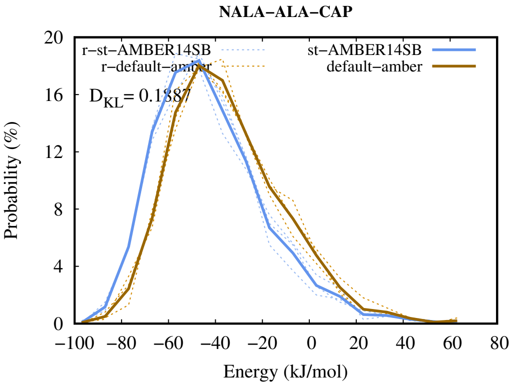
NALA–ALA–CAP
r–st–AMBER14SB
r–default–amber
st–AMBER14SB
default–amber
D$_{KL}$= 0.1887
Probability (%)
Energy (kJ/mol)

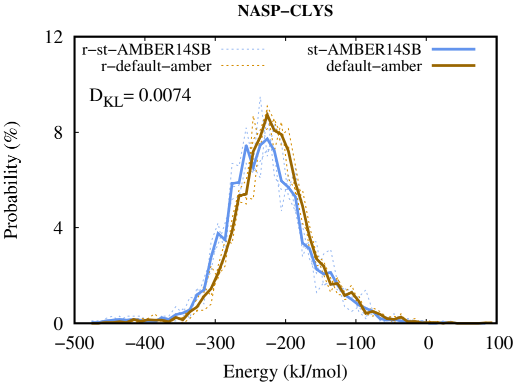
NASP–CLYS
r–st–AMBER14SB
r–default–amber
st–AMBER14SB
default–amber
D$_{KL}$= 0.0074
Probability (%)
Energy (kJ/mol)

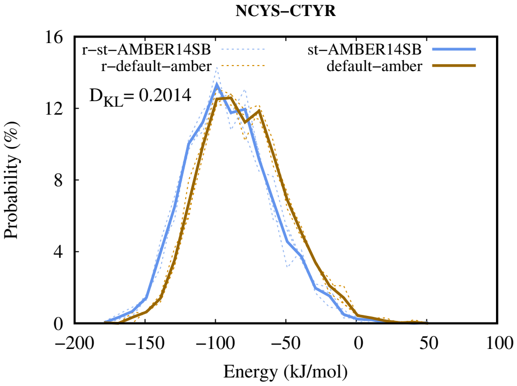
NCYS–CTYR
r–st–AMBER14SB
r–default–amber
st–AMBER14SB
default–amber
D$_{KL}$= 0.2014
Probability (%)
Energy (kJ/mol)

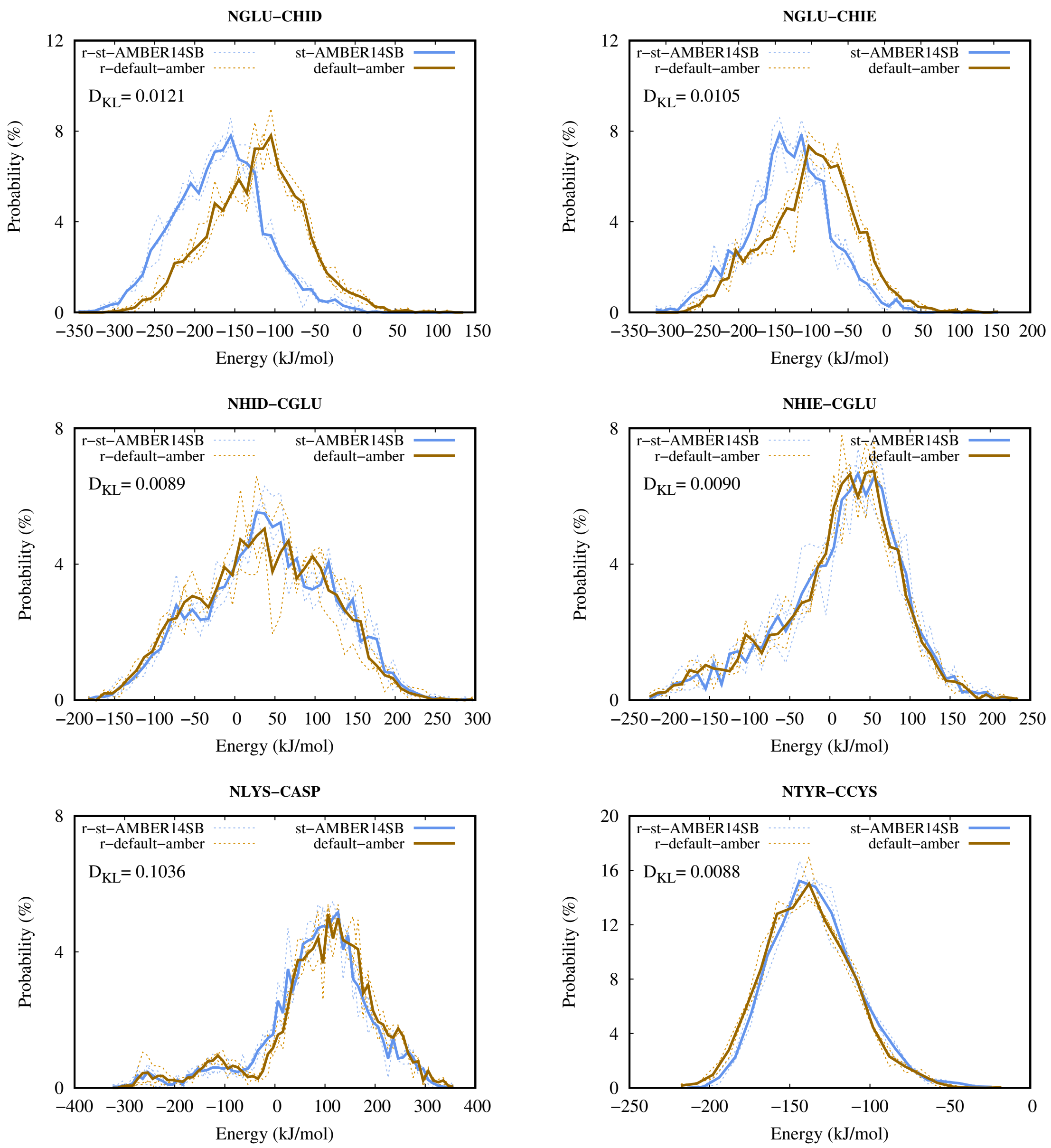

Figure S9: Histogram of the dipeptides Potential Energies ”in vacuum” of GROMACS simulations. The simulations with the changed (st-AMBER14SB, in blue) and with the default charge sets (default-amber, in brown) are shown. The solid lines represent the averages between all replicates (dotted lines). The Kullback-Leibler divergence values[S8] are shown for each distribution.

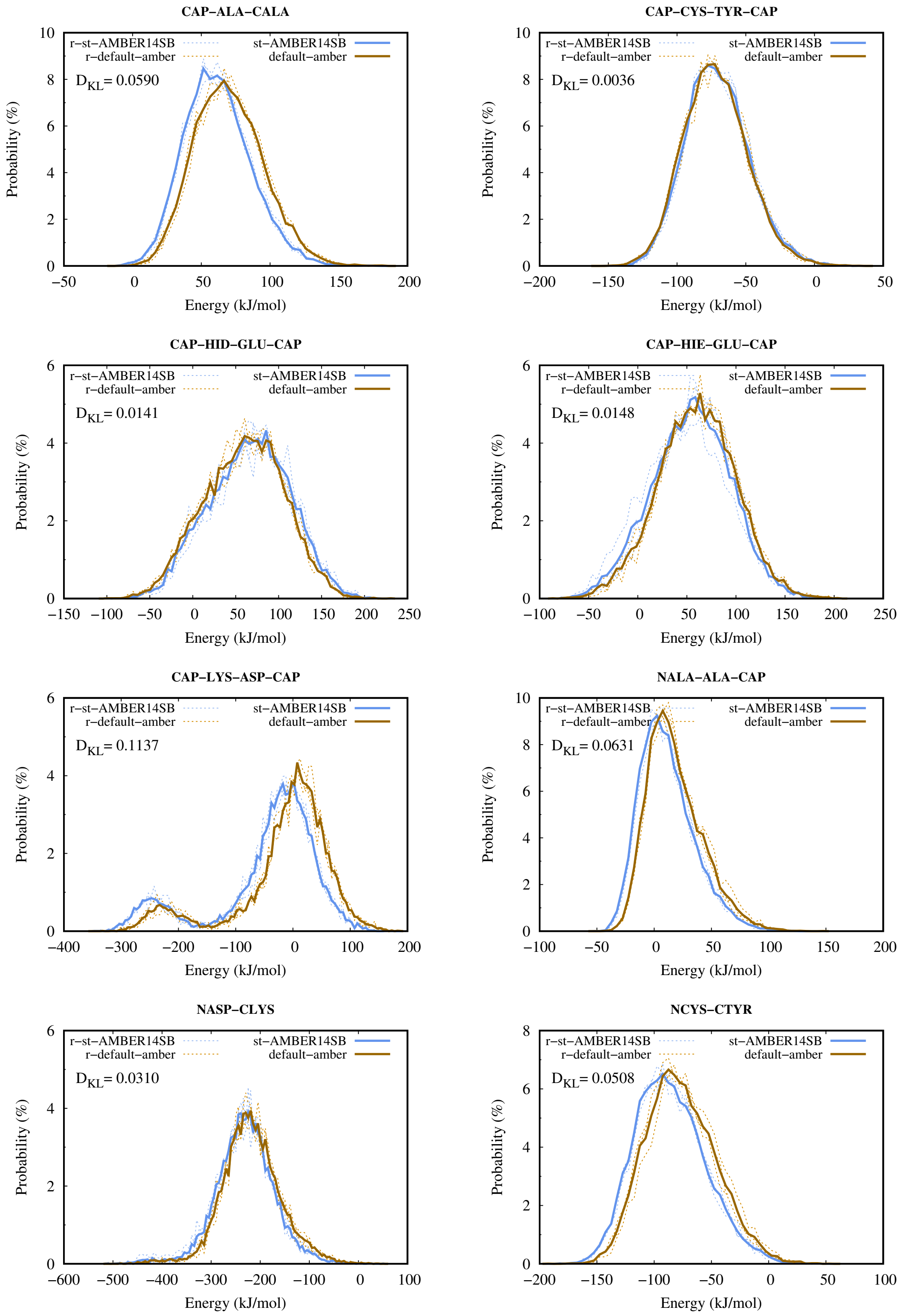
CAP−ALA−CALA
D_KL= 0.0590
CAP−CYS−TYR−CAP
D_KL= 0.0036
CAP−HID−GLU−CAP
D_KL= 0.0141
CAP−HIE−GLU−CAP
D_KL= 0.0148
CAP−LYS−ASP−CAP
D_KL= 0.1137
NALA−ALA−CAP
D_KL= 0.0631
NASP−CLYS
D_KL= 0.0310
NCYS−CTYR
D_KL= 0.0508
r−st−AMBER14SB
r−default−amber
st−AMBER14SB
default−amber
Probability (%)
Energy (kJ/mol)

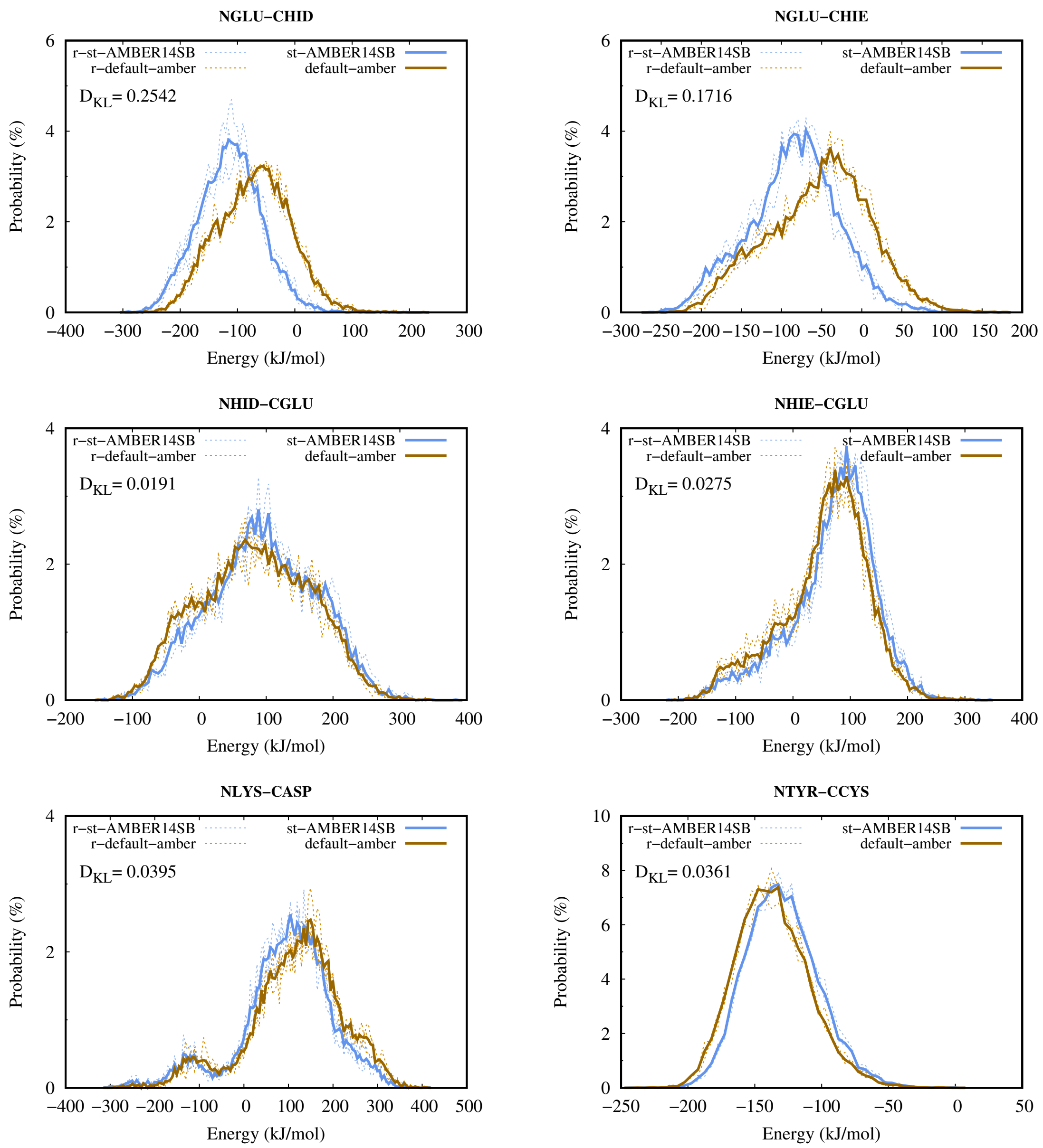

Figure S10: Histogram of the dipeptides Potential Energies "in vacuum" of AMBER simulations. The simulations with the changed (st-AMBER14SB, in blue) and with the default charge sets (default-amber, in brown) are shown. The solid lines represent the averages between all replicates (dotted lines). The Kullback-Leibler divergence values[S8] are shown for each distribution.

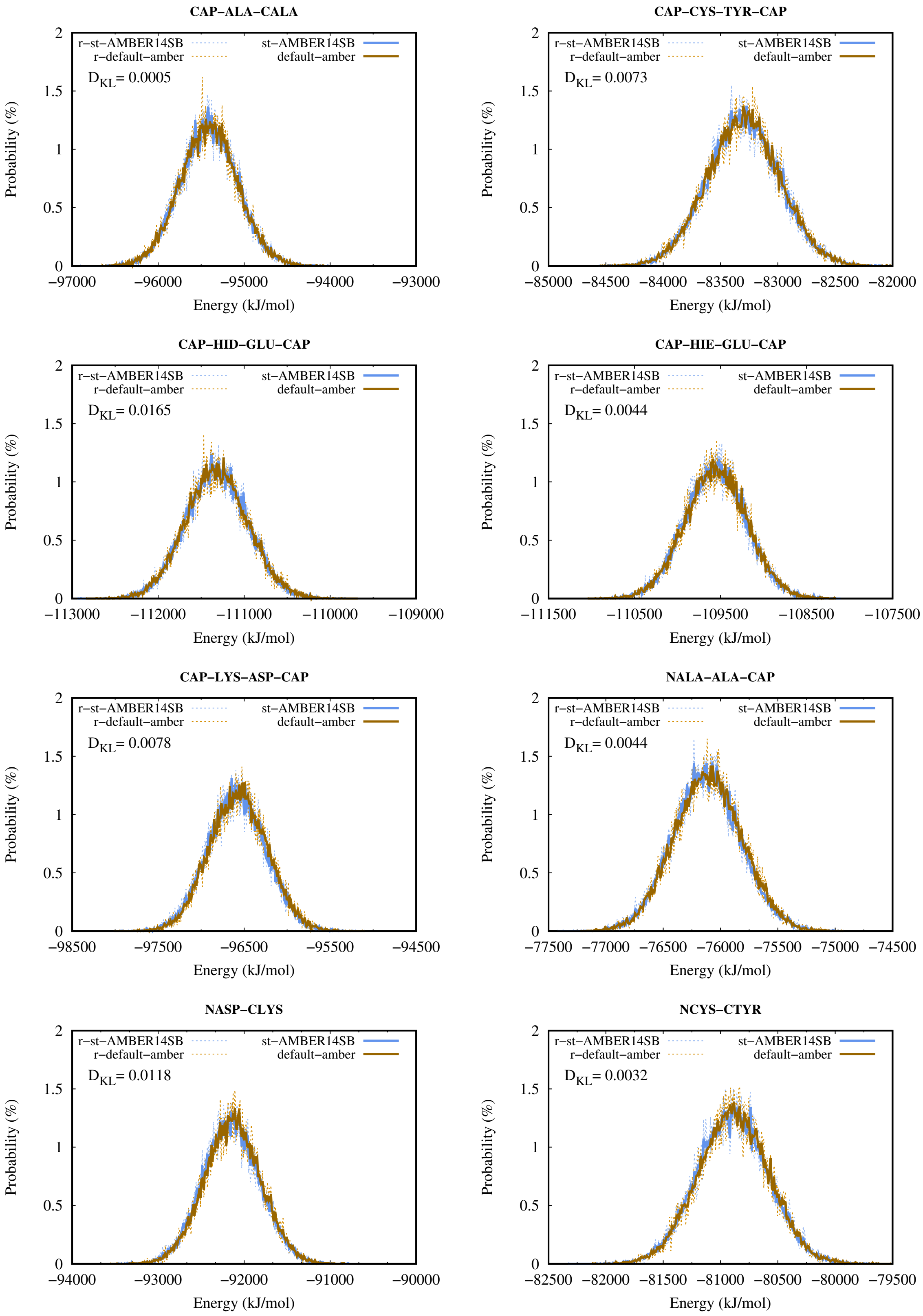
CAP–ALA–CALA
r–st–AMBER14SB
r–default–amber
st–AMBER14SB
default–amber
D_KL= 0.0005
Probability (%)
Energy (kJ/mol)
CAP–CYS–TYR–CAP
D_KL= 0.0073
CAP–HID–GLU–CAP
D_KL= 0.0165
CAP–HIE–GLU–CAP
D_KL= 0.0044
CAP–LYS–ASP–CAP
D_KL= 0.0078
NALA–ALA–CAP
D_KL= 0.0044
NASP–CLYS
D_KL= 0.0118
NCYS–CTYR
D_KL= 0.0032

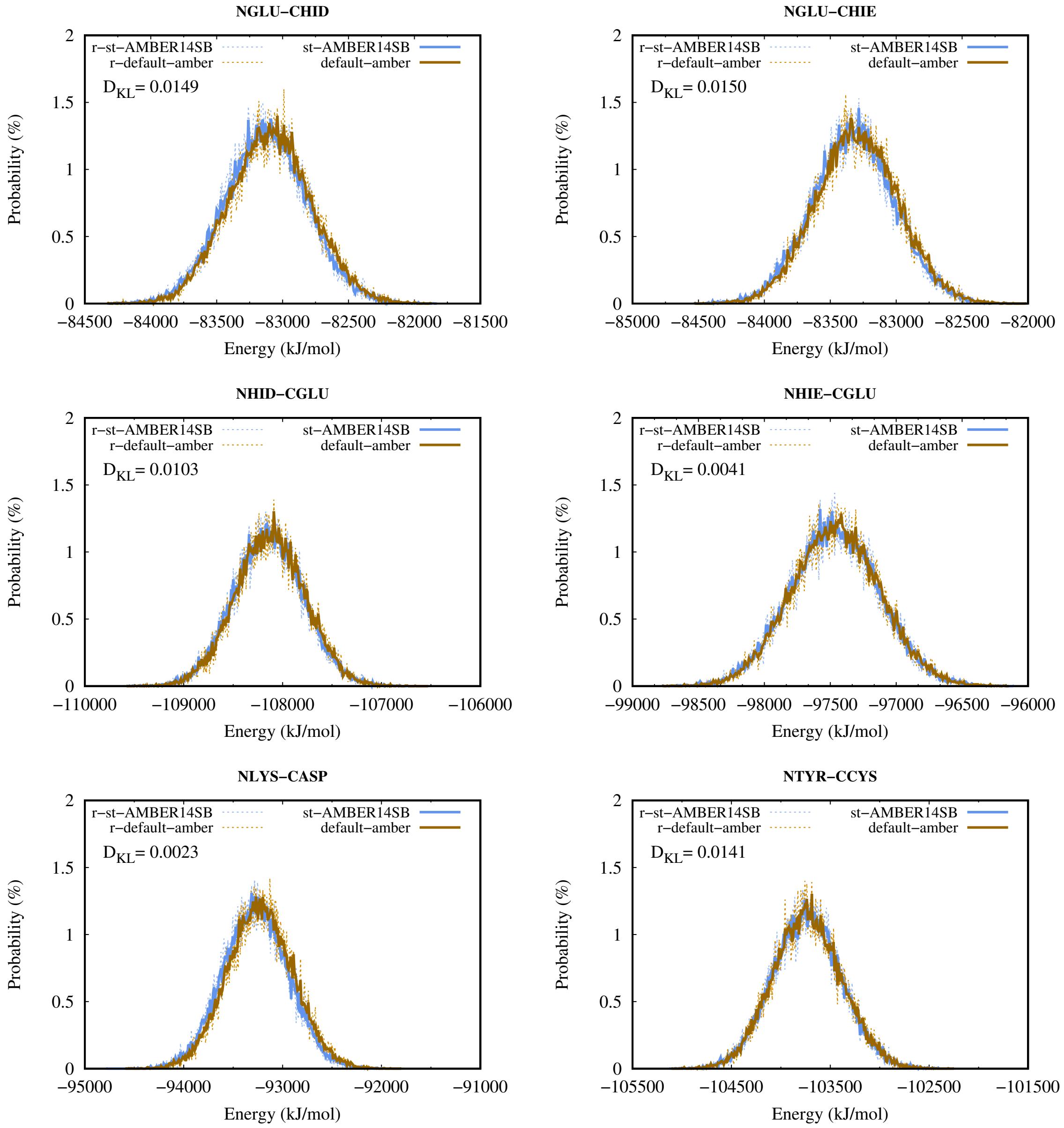

Figure S11: Histogram of the systems Potential Energies in GROMACS simulations. The simulations with the changed (st-AMBER14SB, in blue) and with the default charge sets (default-amber, in brown) are shown. The solid lines represent the averages between all replicates (dotted lines). The Kullback-Leibler divergence values[S8] are shown for each distribution.

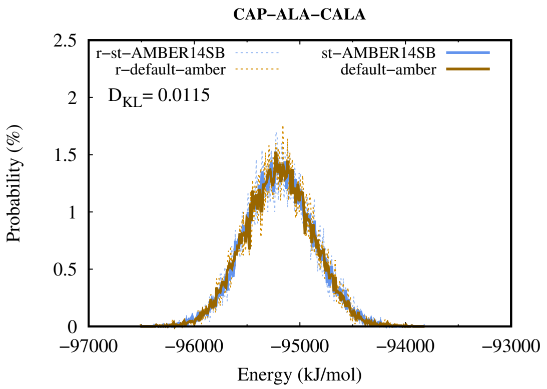
CAP–ALA–CALA
r–st–AMBER14SB
r–default–amber
st–AMBER14SB
default–amber
D_KL= 0.0115
Probability (%)
0 0.5 1 1.5 2 2.5
−97000 −96000 −95000 −94000 −93000
Energy (kJ/mol)

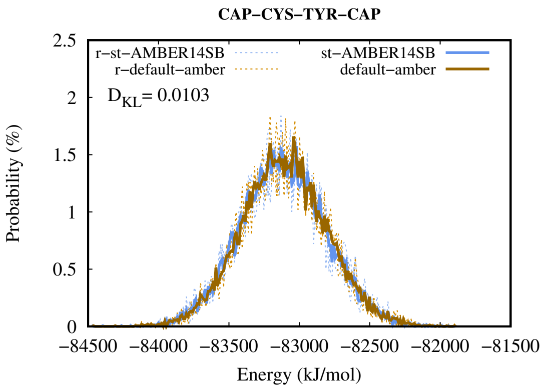
CAP–CYS–TYR–CAP
r–st–AMBER14SB
r–default–amber
st–AMBER14SB
default–amber
D_KL= 0.0103
Probability (%)
0 0.5 1 1.5 2 2.5
−84500 −84000 −83500 −83000 −82500 −82000 −81500
Energy (kJ/mol)

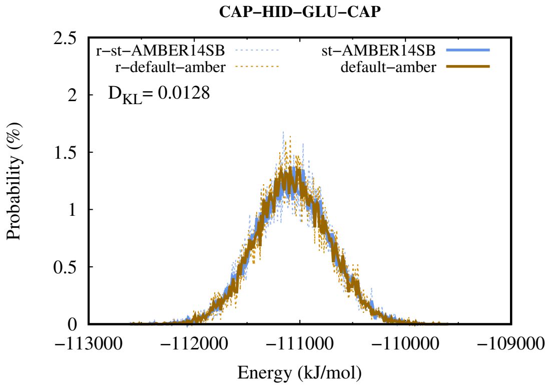
CAP–HID–GLU–CAP
r–st–AMBER14SB
r–default–amber
st–AMBER14SB
default–amber
D_KL= 0.0128
Probability (%)
0 0.5 1 1.5 2 2.5
−113000 −112000 −111000 −110000 −109000
Energy (kJ/mol)

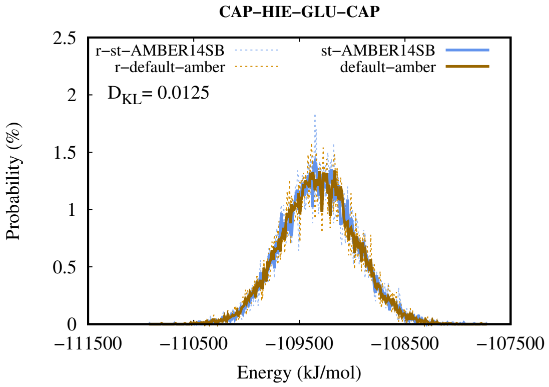
CAP–HIE–GLU–CAP
r–st–AMBER14SB
r–default–amber
st–AMBER14SB
default–amber
D_KL= 0.0125
Probability (%)
0 0.5 1 1.5 2 2.5
−111500 −110500 −109500 −108500 −107500
Energy (kJ/mol)

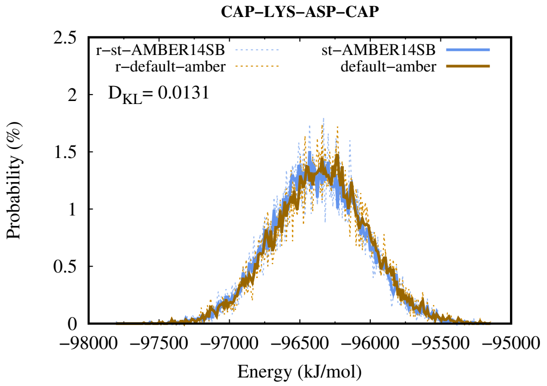
CAP–LYS–ASP–CAP
r–st–AMBER14SB
r–default–amber
st–AMBER14SB
default–amber
D_KL= 0.0131
Probability (%)
0 0.5 1 1.5 2 2.5
−98000 −97500 −97000 −96500 −96000 −95500 −95000
Energy (kJ/mol)

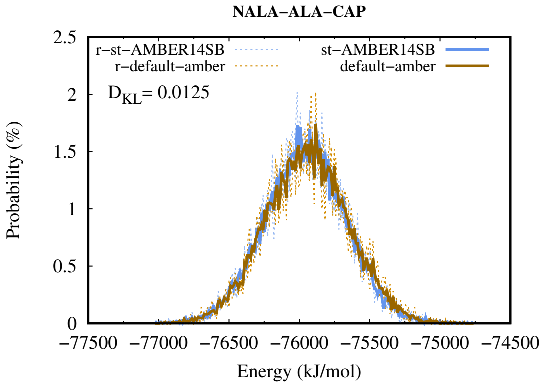
NALA–ALA–CAP
r–st–AMBER14SB
r–default–amber
st–AMBER14SB
default–amber
D_KL= 0.0125
Probability (%)
0 0.5 1 1.5 2 2.5
−77500 −77000 −76500 −76000 −75500 −75000 −74500
Energy (kJ/mol)

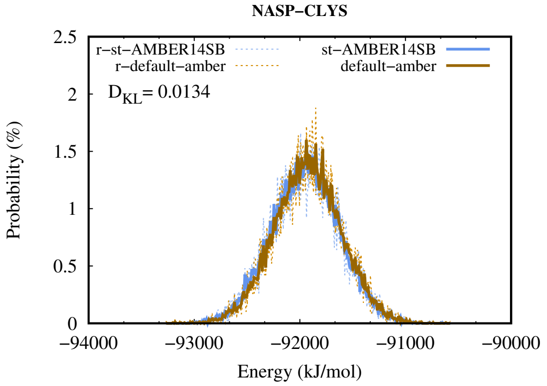
NASP–CLYS
r–st–AMBER14SB
r–default–amber
st–AMBER14SB
default–amber
D_KL= 0.0134
Probability (%)
0 0.5 1 1.5 2 2.5
−94000 −93000 −92000 −91000 −90000
Energy (kJ/mol)

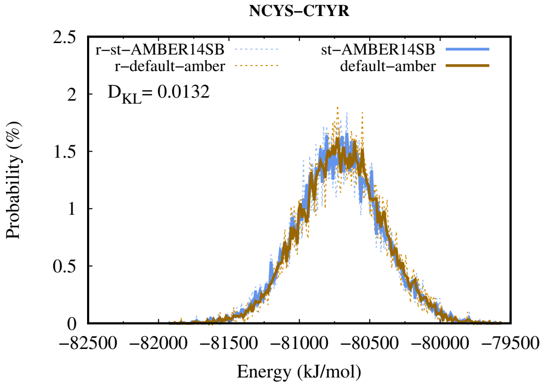
NCYS–CTYR
r–st–AMBER14SB
r–default–amber
st–AMBER14SB
default–amber
D_KL= 0.0132
Probability (%)
0 0.5 1 1.5 2 2.5
−82500 −82000 −81500 −81000 −80500 −80000 −79500
Energy (kJ/mol)

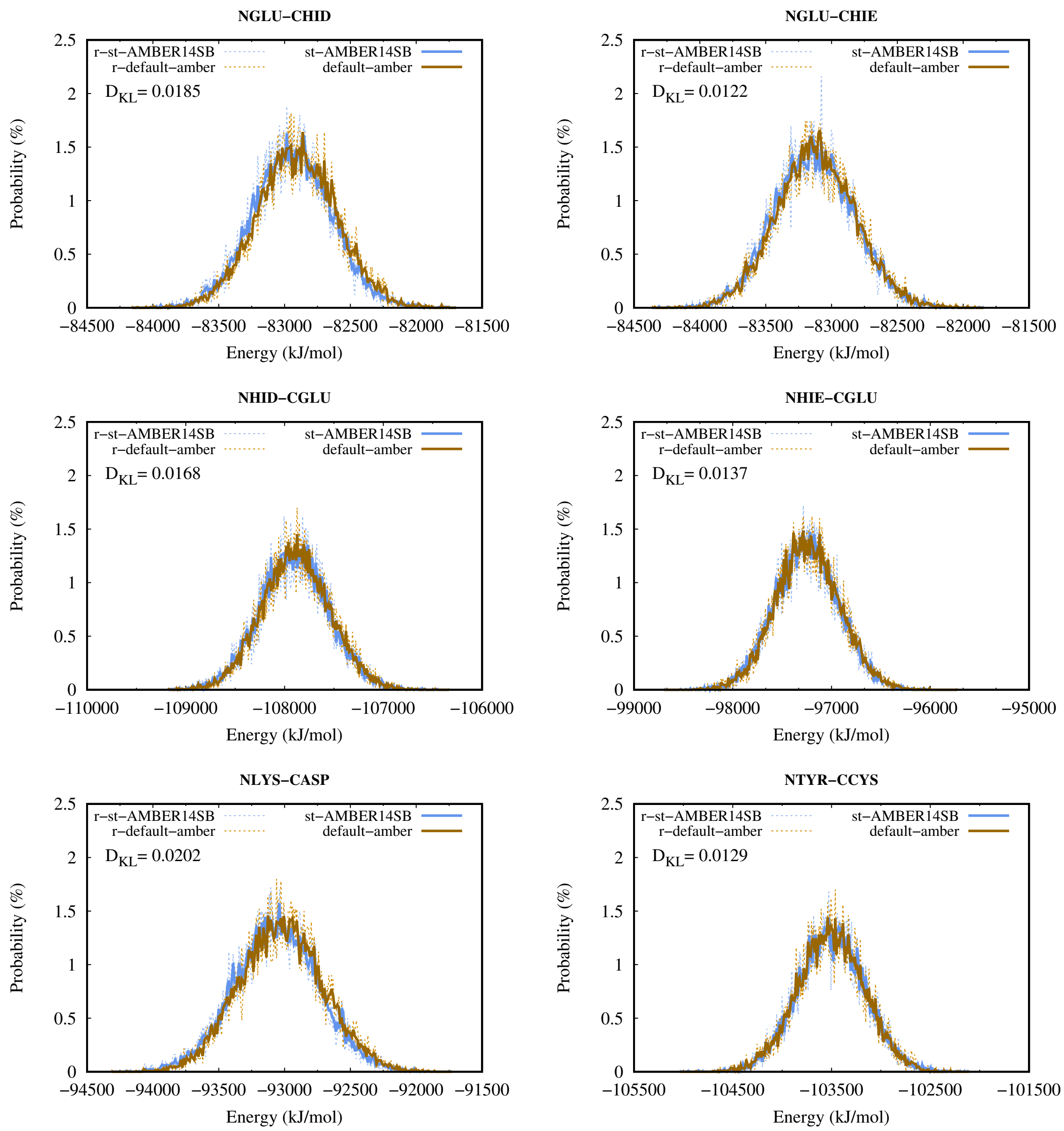

Figure S12: Histogram of the systems Potential Energies in AMBER simulations. The simulations with the changed (st-AMBER14SB, in blue) and with the default charge sets (default-amber, in brown) are shown. The solid lines represent the averages between all replicates (dotted lines). The Kullback-Leibler divergence values[S8] are shown for each distribution.

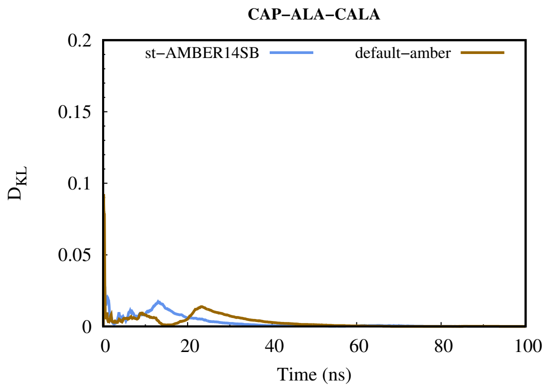
CAP−ALA−CALA
st−AMBER14SB
default−amber
D_KL
Time (ns)

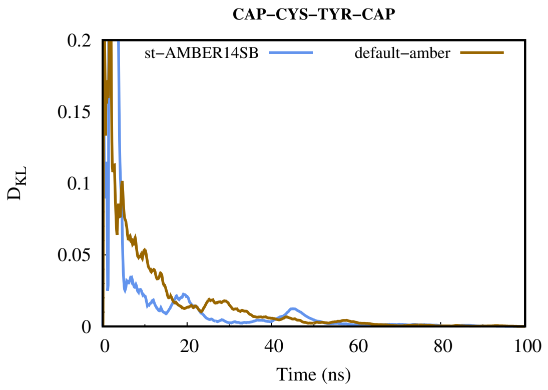
CAP−CYS−TYR−CAP
st−AMBER14SB
default−amber
D_KL
Time (ns)

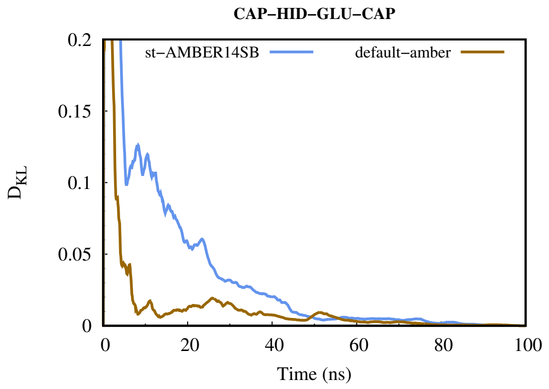
CAP−HID−GLU−CAP
st−AMBER14SB
default−amber
D_KL
Time (ns)

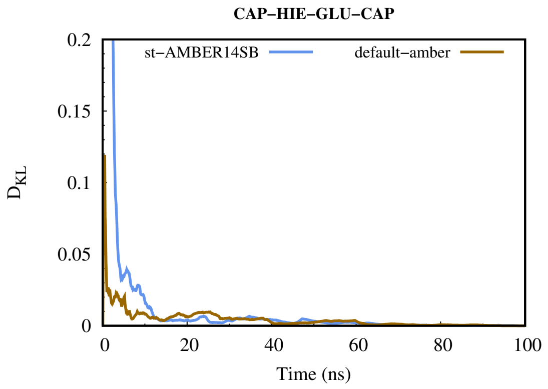
CAP−HIE−GLU−CAP
st−AMBER14SB
default−amber
D_KL
Time (ns)

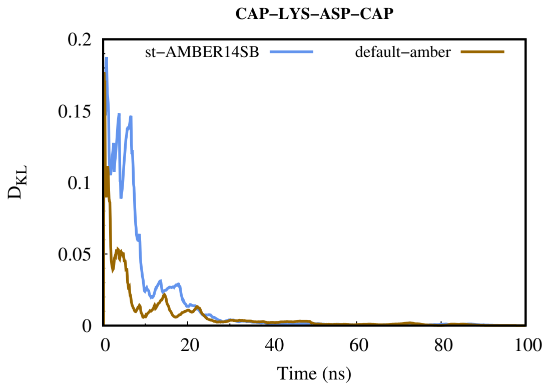
CAP−LYS−ASP−CAP
st−AMBER14SB
default−amber
D_KL
Time (ns)

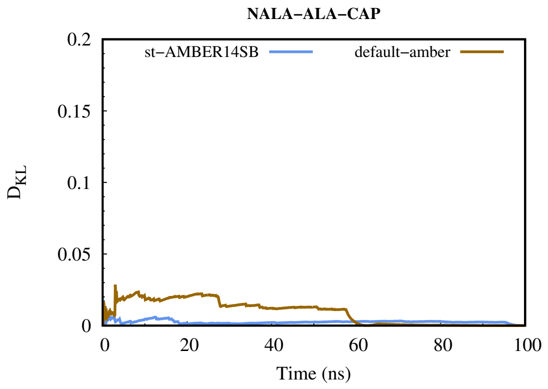
NALA−ALA−CAP
st−AMBER14SB
default−amber
D_KL
Time (ns)

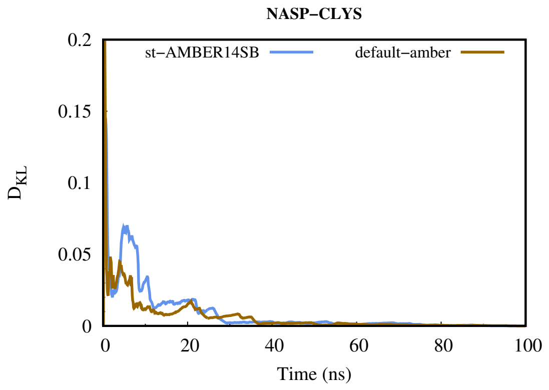
NASP−CLYS
st−AMBER14SB
default−amber
D_KL
Time (ns)

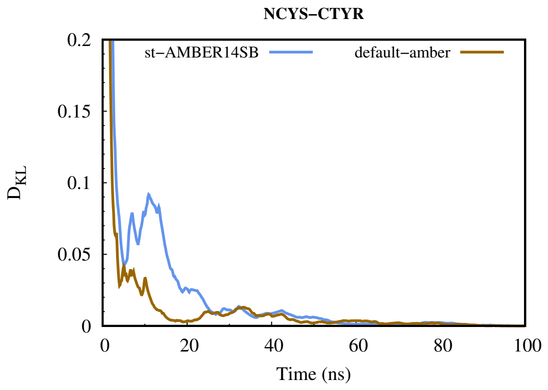
NCYS−CTYR
st−AMBER14SB
default−amber
D_KL
Time (ns)

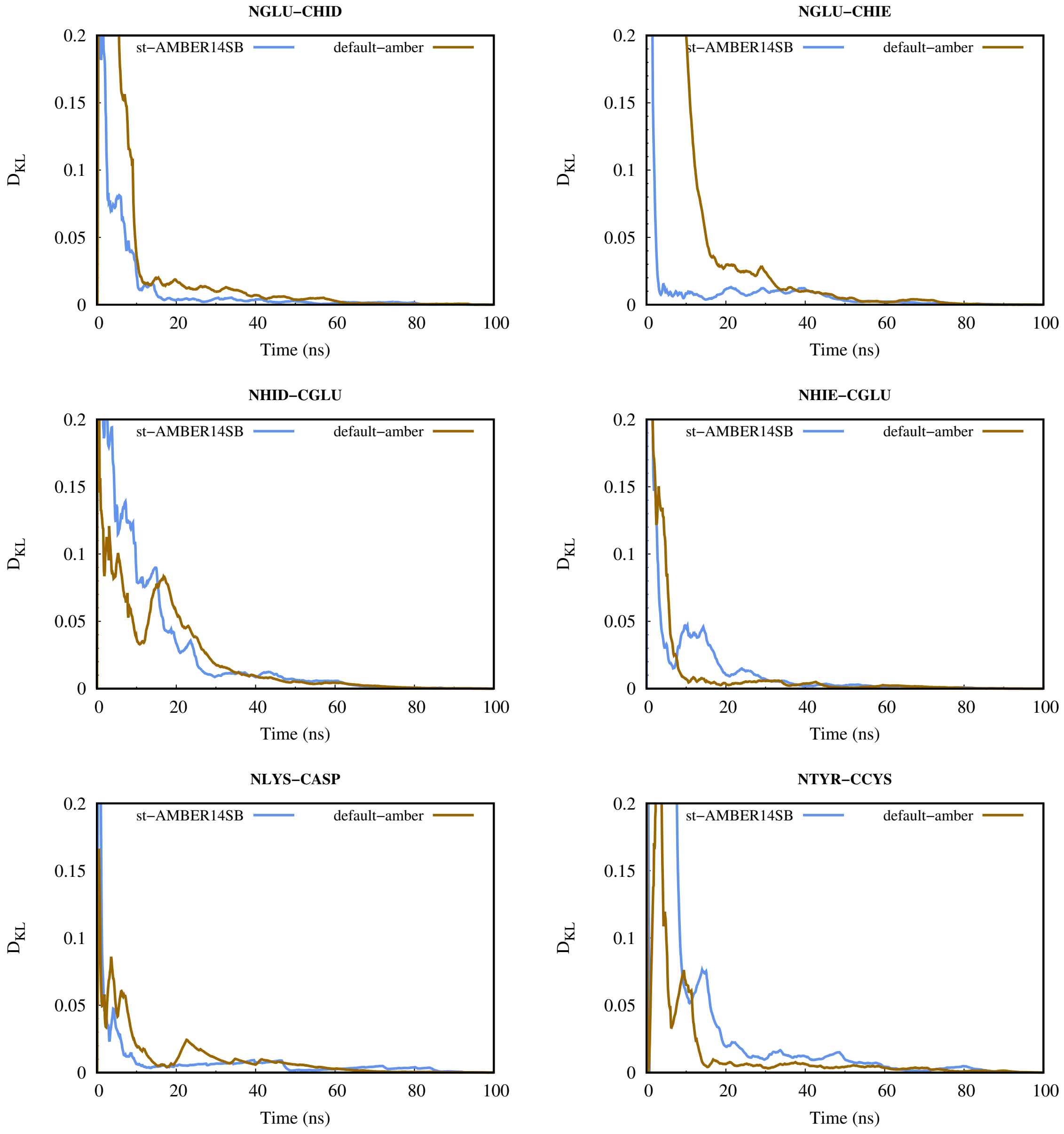

Figure S13: Kullback-Leibler divergence[S8] as a function of time for the side chain distance histograms. The blue and brown lines depict the simulations with the changed (st-AMBER14SB) and default force fields, respectively.

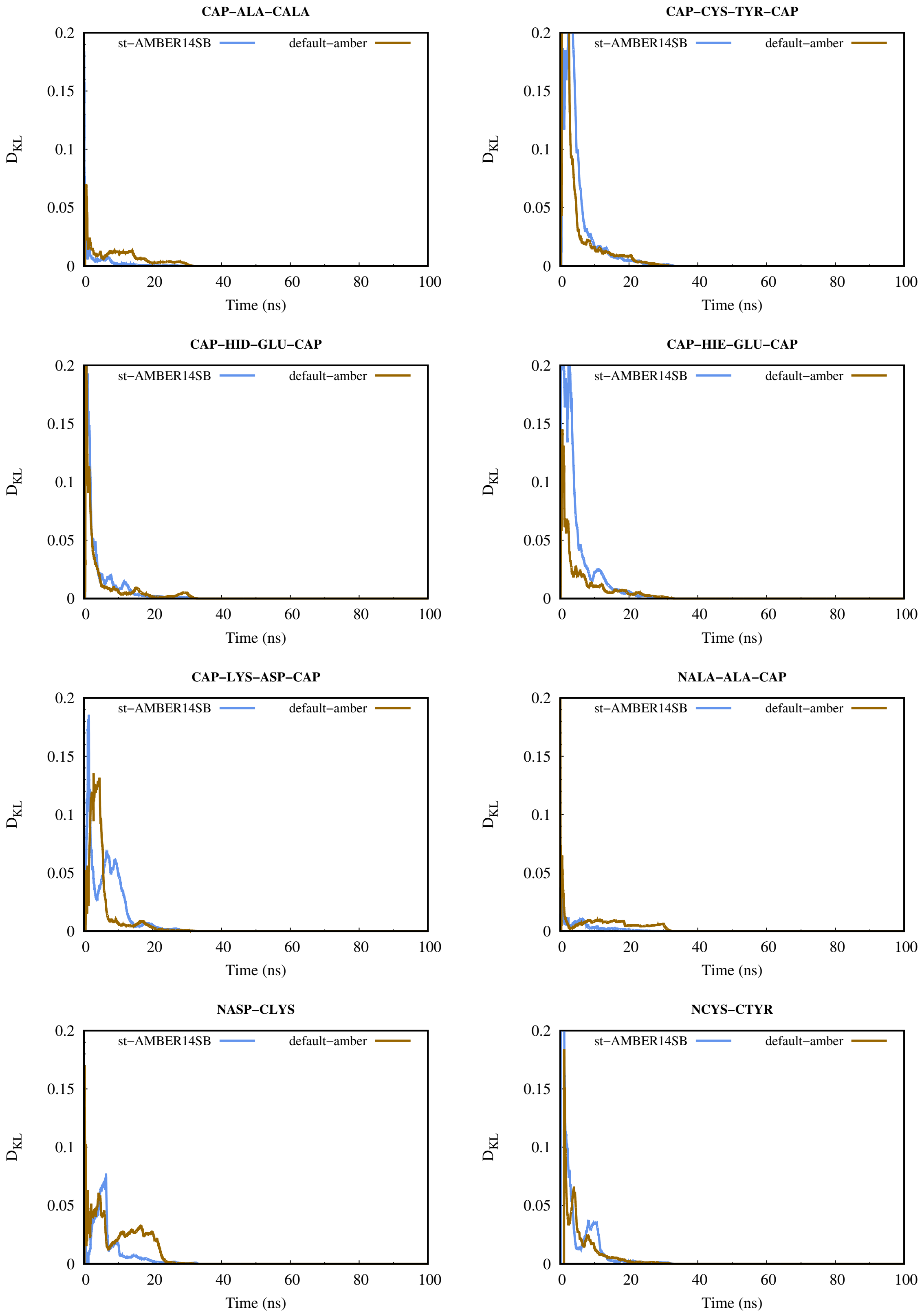
CAP−ALA−CALA
CAP−CYS−TYR−CAP
CAP−HID−GLU−CAP
CAP−HIE−GLU−CAP
CAP−LYS−ASP−CAP
NALA−ALA−CAP
NASP−CLYS
NCYS−CTYR
st−AMBER14SB
default−amber
D_{KL}
Time (ns)
0
0.05
0.1
0.15
0.2
20
40
60
80
100

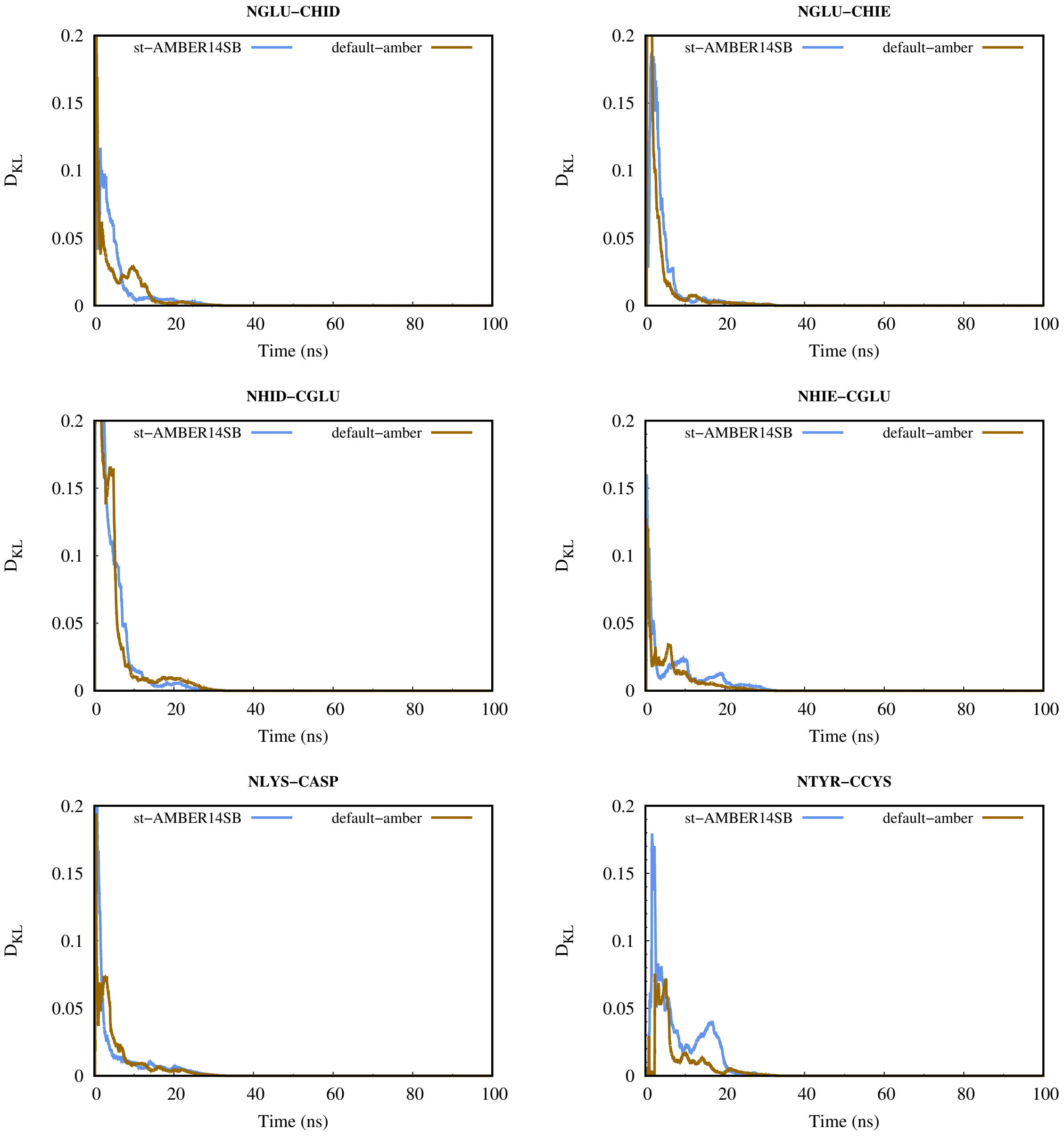

Figure S14: Kullback-Leibler divergence[S8] as a function of time for the side chain distance histograms of the simulations using Amber. The blue and brown lines depict the simulations with the changed and default force fields, respectively.

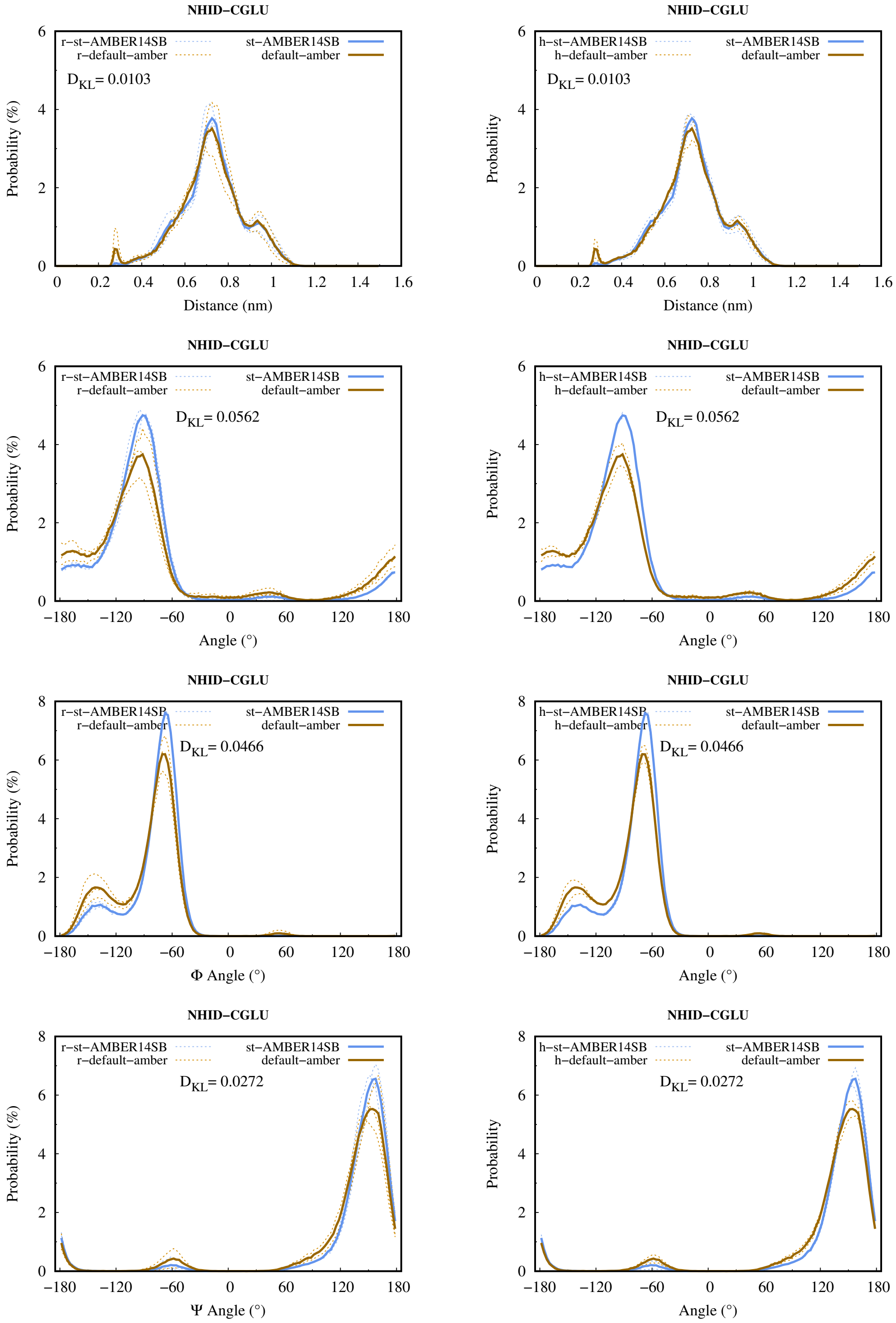

Figure S15: Comparison between the sampling obtained when the simulations were considered in thirds (left) and in halves (right). The analyses depicted are, from top to bottom, the side chain distance, the $C\beta - C\alpha - C\alpha - C\beta$ pseudo dihedral and the Φ and Ψ dihedrals. Only the NHID-CGLU dipeptide Gromacs simulations are shown.

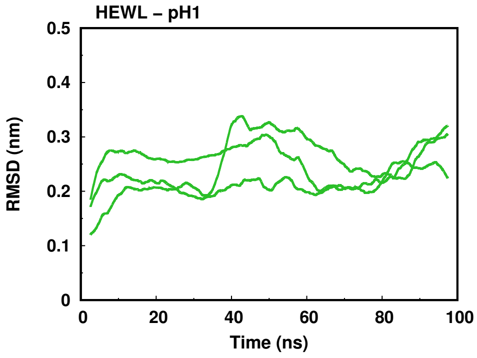
HEWL – pH1
RMSD (nm)
Time (ns)
0
0.1
0.2
0.3
0.4
0.5
0
20
40
60
80
100

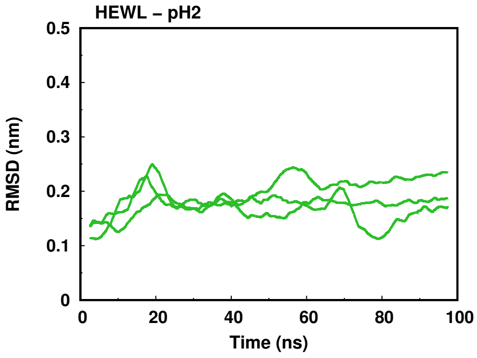
HEWL – pH2
RMSD (nm)
Time (ns)
0
0.1
0.2
0.3
0.4
0.5
0
20
40
60
80
100

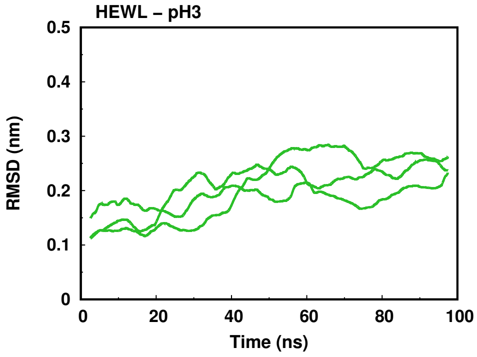
HEWL – pH3
RMSD (nm)
Time (ns)
0
0.1
0.2
0.3
0.4
0.5
0
20
40
60
80
100

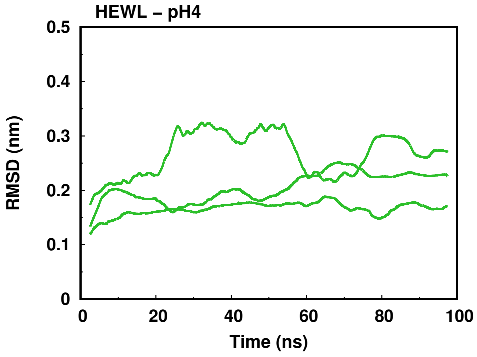
HEWL – pH4
RMSD (nm)
Time (ns)
0
0.1
0.2
0.3
0.4
0.5
0
20
40
60
80
100

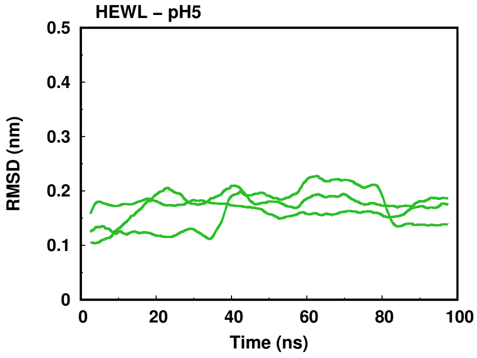
HEWL – pH5
RMSD (nm)
Time (ns)
0
0.1
0.2
0.3
0.4
0.5
0
20
40
60
80
100

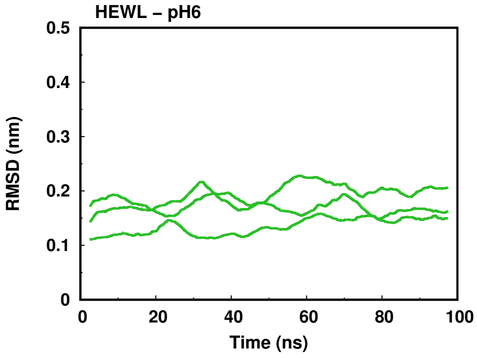
HEWL – pH6
RMSD (nm)
Time (ns)
0
0.1
0.2
0.3
0.4
0.5
0
20
40
60
80
100

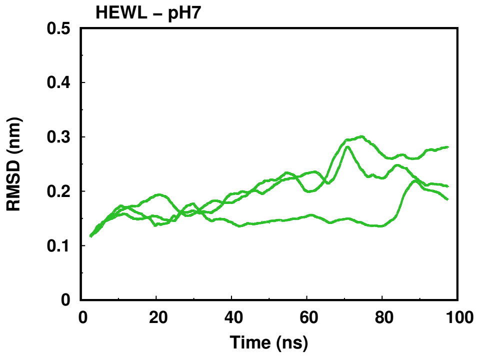
HEWL – pH7
RMSD (nm)
Time (ns)
0
0.1
0.2
0.3
0.4
0.5
0
20
40
60
80
100

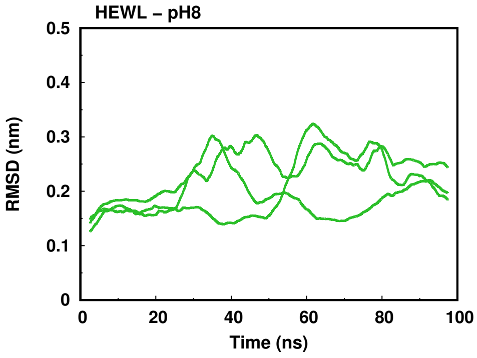
HEWL – pH8
RMSD (nm)
Time (ns)
0
0.1
0.2
0.3
0.4
0.5
0
20
40
60
80
100

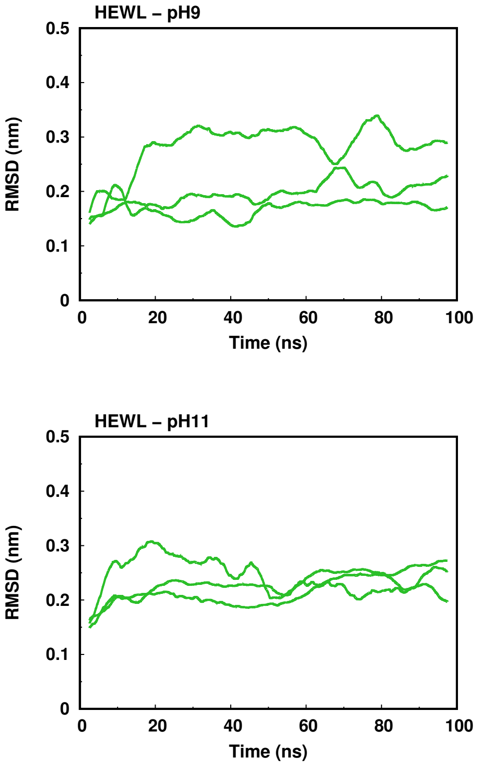

Figure S16: Cα RMSD evolution with time for HEWL, across all simulated pH values, using the sampling of 0.5 ns sliding windows.

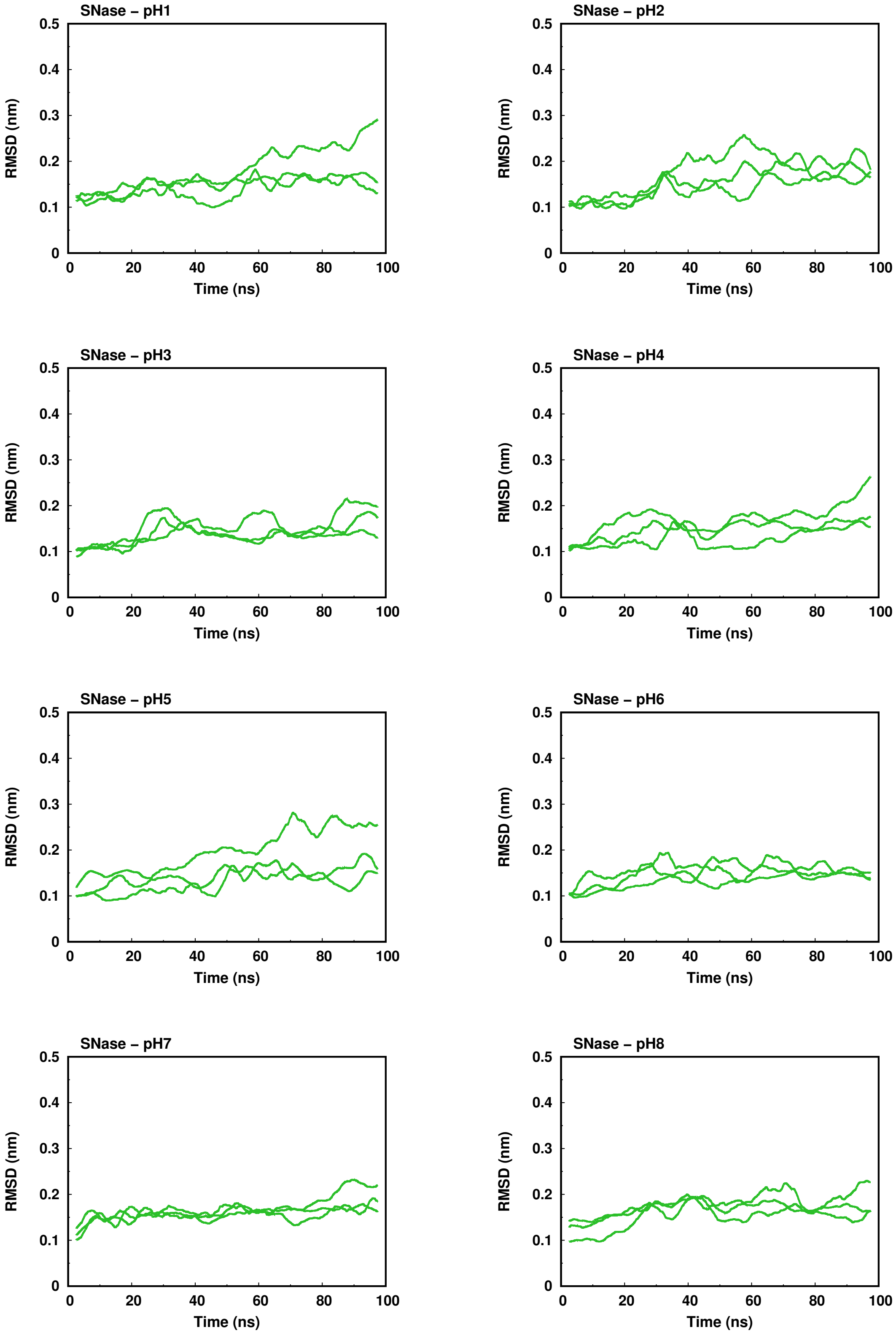
SNase – pH1
SNase – pH2
SNase – pH3
SNase – pH4
SNase – pH5
SNase – pH6
SNase – pH7
SNase – pH8
RMSD (nm)
Time (ns)
0
0.1
0.2
0.3
0.4
0.5
0
20
40
60
80
100

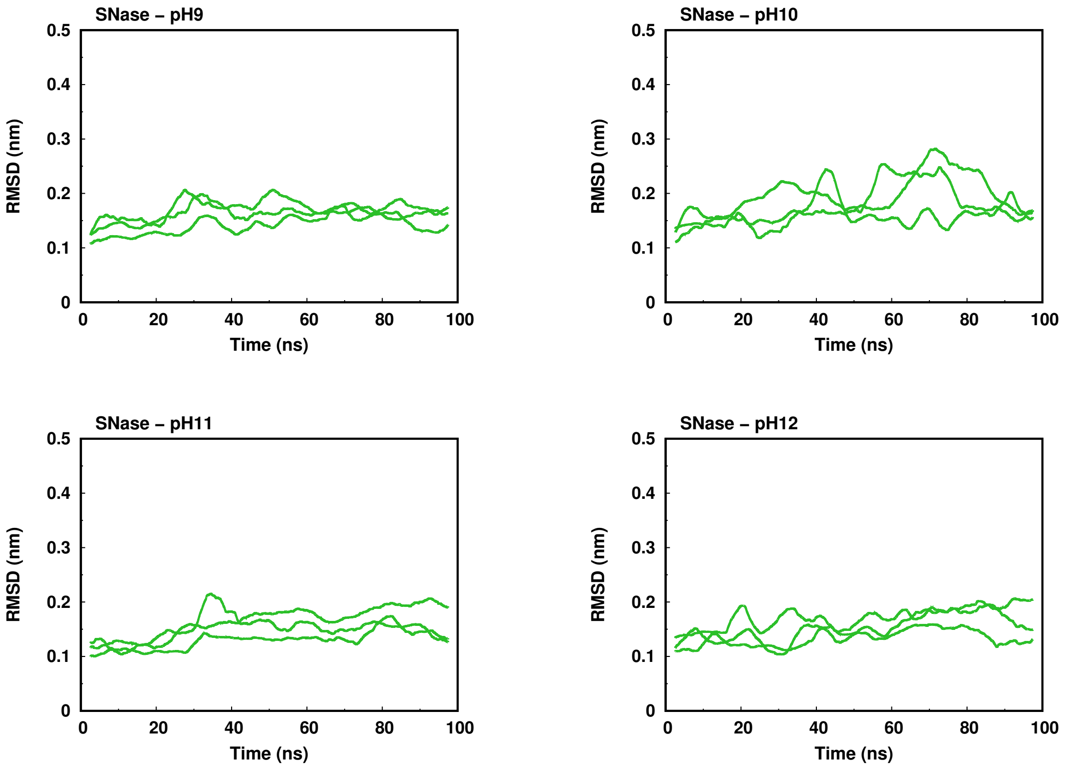

Figure S17: Cα RMSD evolution with time for SNase, across all simulated pH values, using the sampling of 0.5 ns sliding windows.

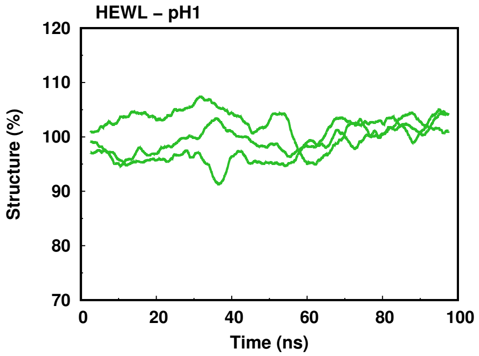
HEWL – pH1
Structure (%)
Time (ns)
120
110
100
90
80
70
0
20
40
60
80
100

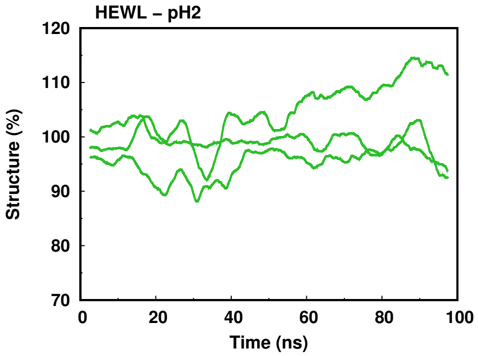
HEWL – pH2
Structure (%)
Time (ns)
120
110
100
90
80
70
0
20
40
60
80
100

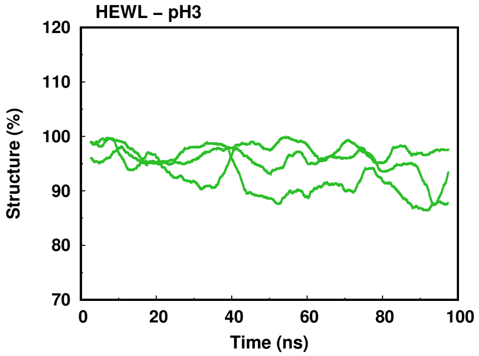
HEWL – pH3
Structure (%)
Time (ns)
120
110
100
90
80
70
0
20
40
60
80
100

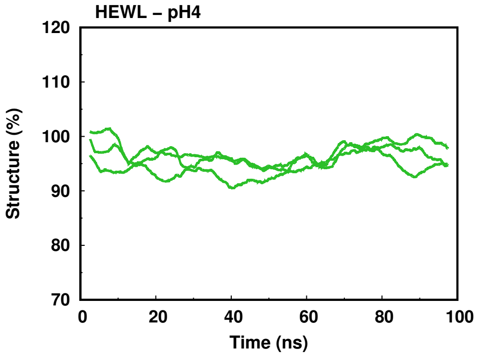
HEWL – pH4
Structure (%)
Time (ns)
120
110
100
90
80
70
0
20
40
60
80
100

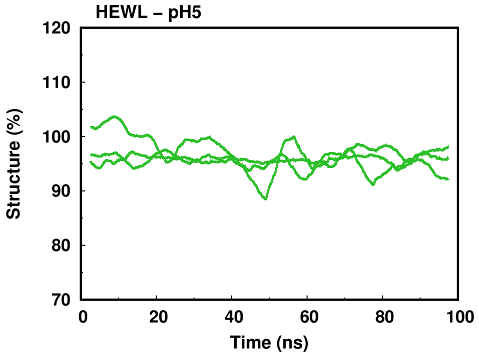
HEWL – pH5
Structure (%)
Time (ns)
120
110
100
90
80
70
0
20
40
60
80
100

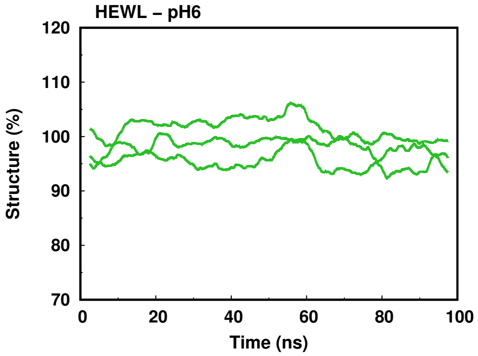
HEWL – pH6
Structure (%)
Time (ns)
120
110
100
90
80
70
0
20
40
60
80
100

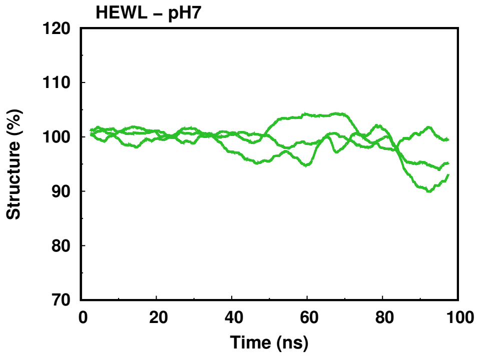
HEWL – pH7
Structure (%)
Time (ns)
120
110
100
90
80
70
0
20
40
60
80
100

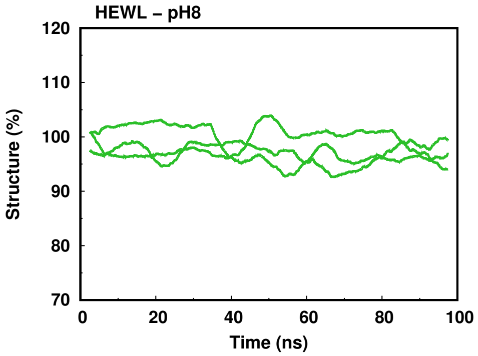
HEWL – pH8
Structure (%)
Time (ns)
120
110
100
90
80
70
0
20
40
60
80
100

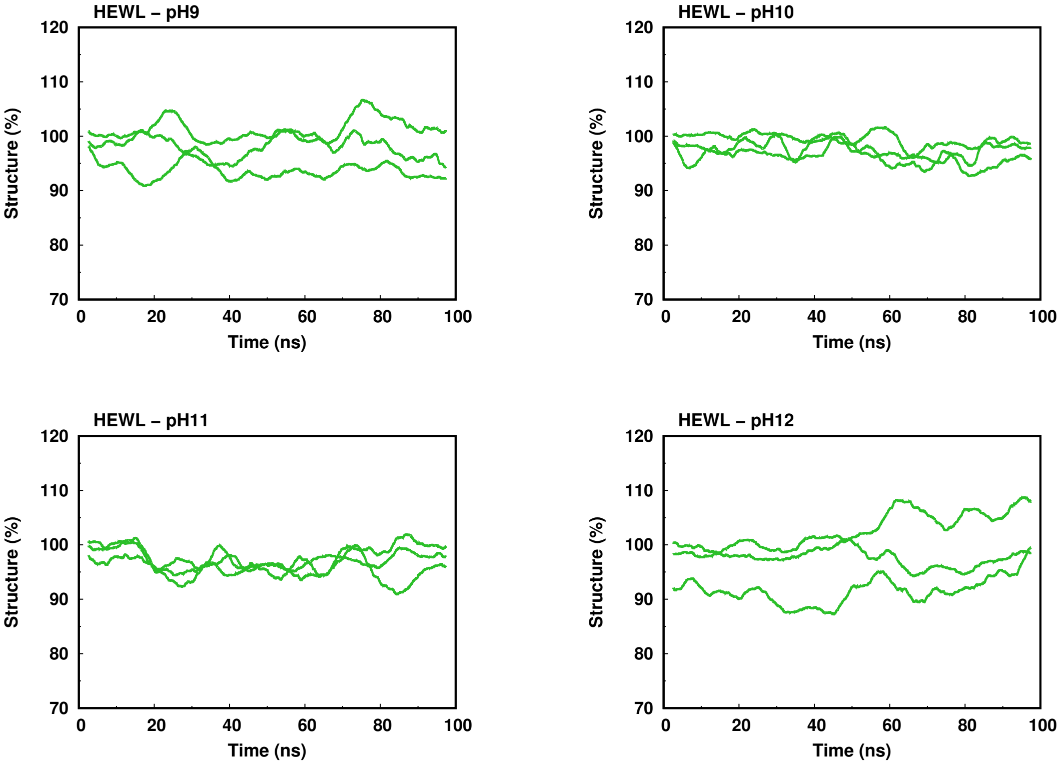

Figure S18: Secondary structure evolution for HEWL, across simulated pH values, using the sampling of 0.5 ns sliding windows. The structure represents the sum of all residues in helical or β-sheet conformations and its percentages were calculated by comparing to the experimental structures (assumed to be 100%). The secondary structure of all structures was calculated using the GROMACS *dssp* tool.

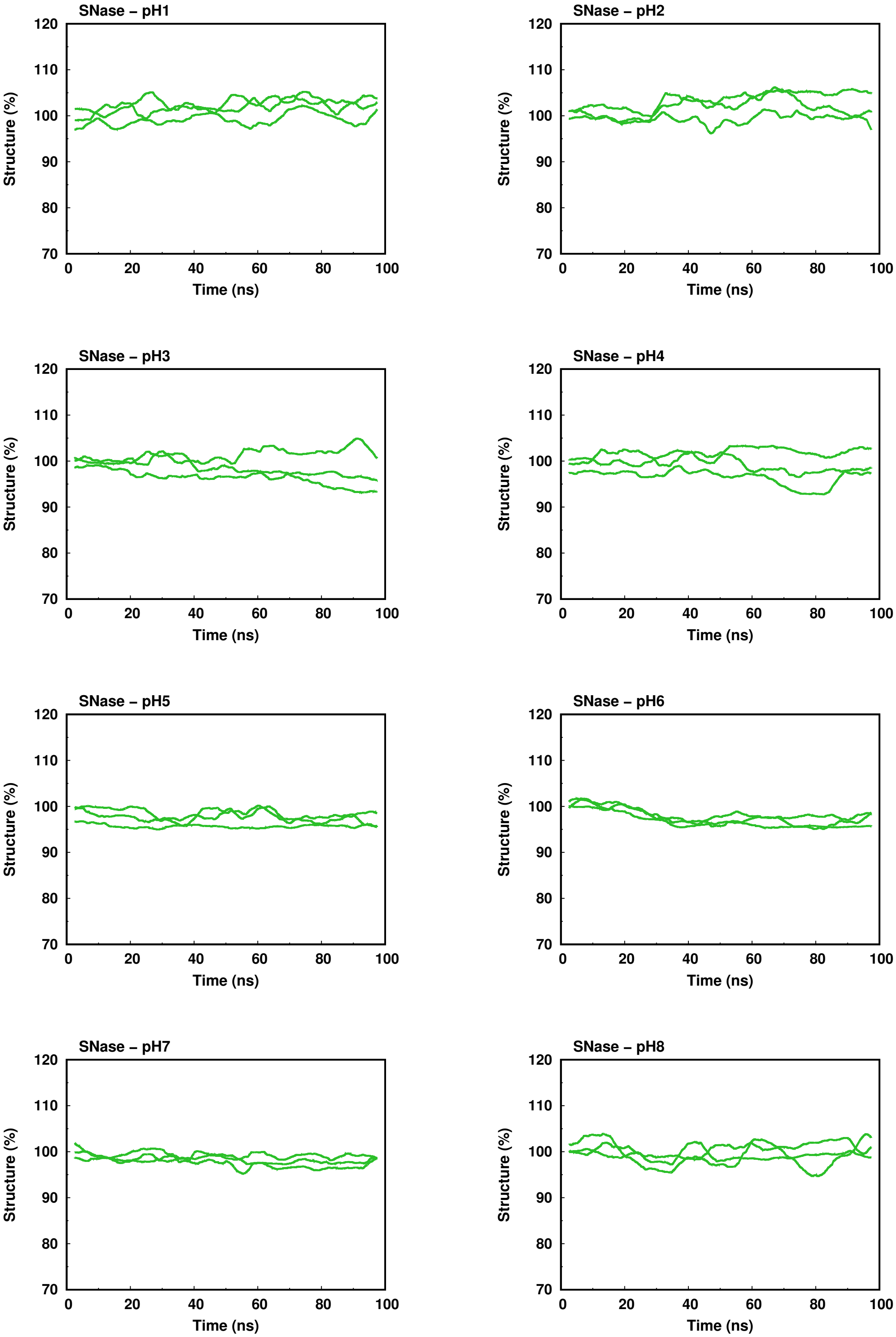
SNase – pH1
SNase – pH2
SNase – pH3
SNase – pH4
SNase – pH5
SNase – pH6
SNase – pH7
SNase – pH8
Structure (%)
Time (ns)
120
110
100
90
80
70
0
20
40
60
80
100

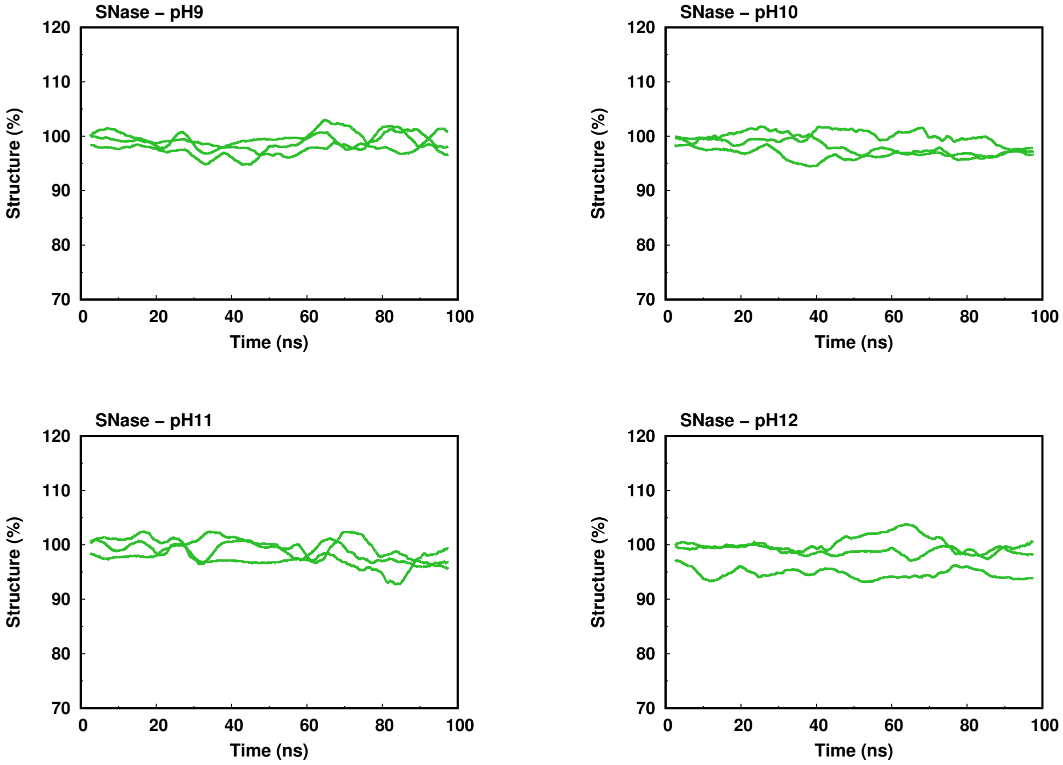

Figure S19: Secondary structure evolution for SNase, across simulated pH values, using the sampling of 0.5 ns sliding windows. The structure represents the sum of all residues in helical or β-sheet conformations and its percentages were calculated by comparing to the experimental structure (assumed to be 100%). The secondary structure of all structures was calculated using the GROMACS *dssp* tool.

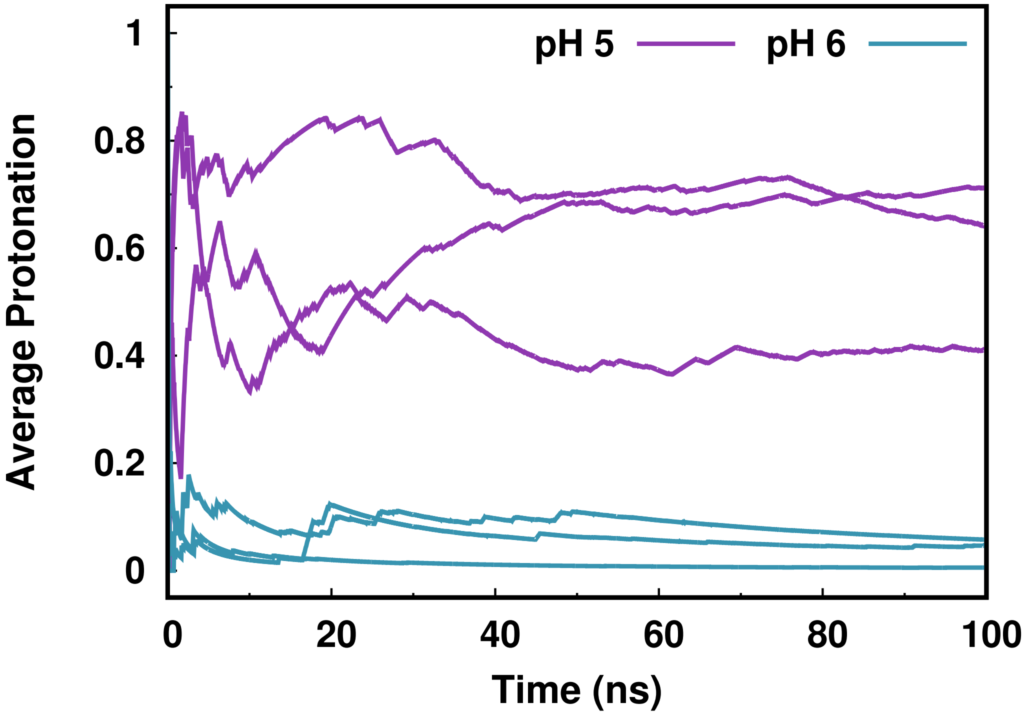

Figure S20: Accumulated average protonation over time of HEWL E35. Triplicates of simulations with pH 5 and 6 (values closest to the estimated pK_a) are represented.

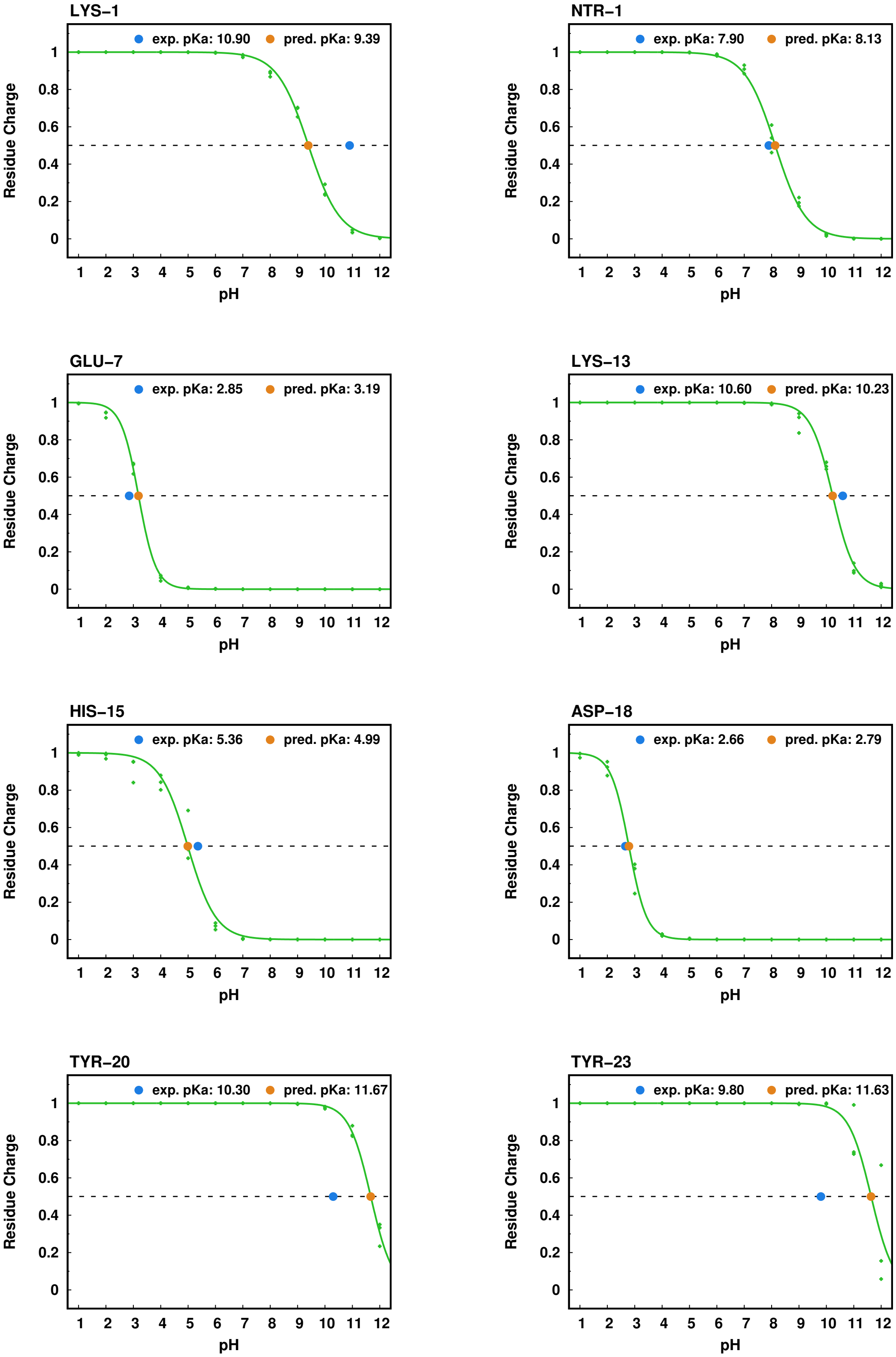

LYS–1
exp. pKa: 10.90
pred. pKa: 9.39
NTR–1
exp. pKa: 7.90
pred. pKa: 8.13
GLU–7
exp. pKa: 2.85
pred. pKa: 3.19
LYS–13
exp. pKa: 10.60
pred. pKa: 10.23
HIS–15
exp. pKa: 5.36
pred. pKa: 4.99
ASP–18
exp. pKa: 2.66
pred. pKa: 2.79
TYR–20
exp. pKa: 10.30
pred. pKa: 11.67
TYR–23
exp. pKa: 9.80
pred. pKa: 11.63
Residue Charge
pH

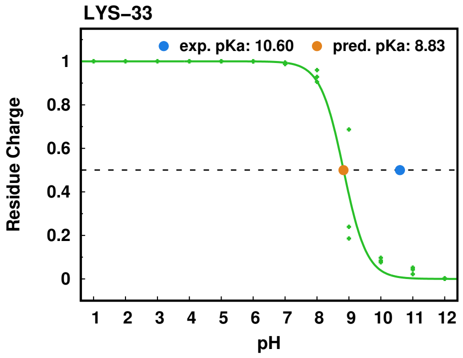
LYS–33
exp. pKa: 10.60
pred. pKa: 8.83
Residue Charge
pH

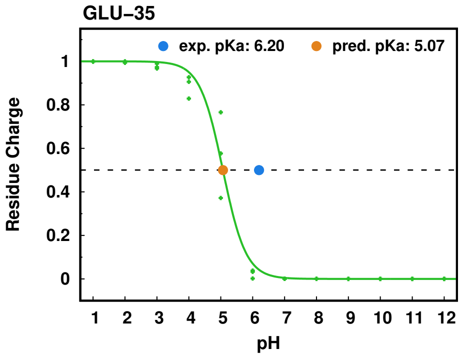
GLU–35
exp. pKa: 6.20
pred. pKa: 5.07
Residue Charge
pH

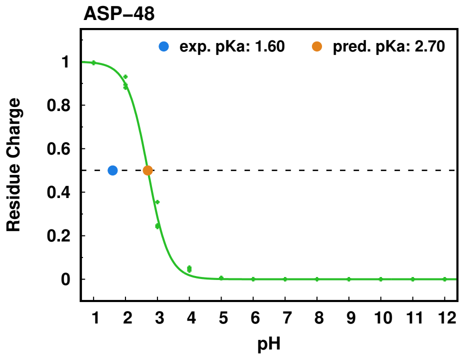
ASP–48
exp. pKa: 1.60
pred. pKa: 2.70
Residue Charge
pH

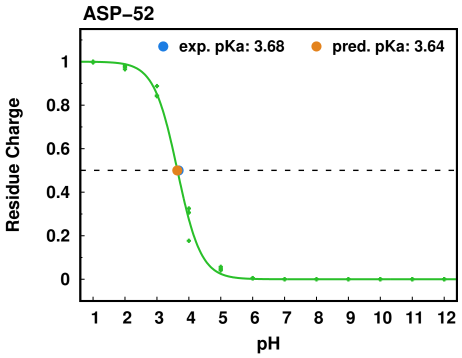
ASP–52
exp. pKa: 3.68
pred. pKa: 3.64
Residue Charge
pH

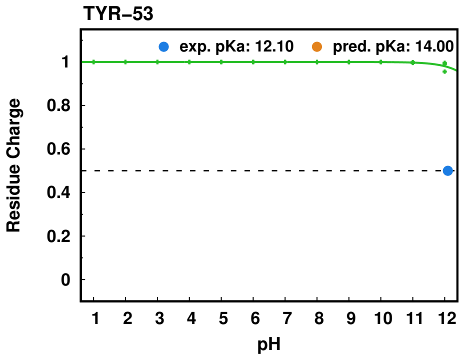
TYR–53
exp. pKa: 12.10
pred. pKa: 14.00
Residue Charge
pH

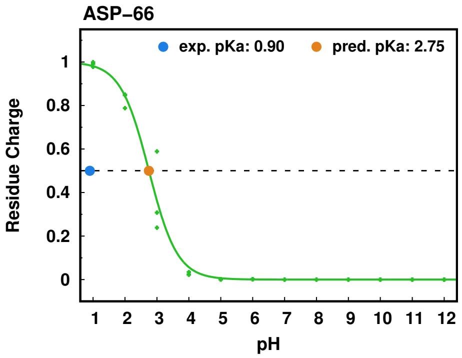
ASP–66
exp. pKa: 0.90
pred. pKa: 2.75
Residue Charge
pH

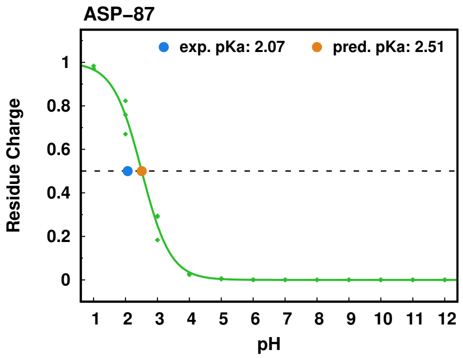
ASP–87
exp. pKa: 2.07
pred. pKa: 2.51
Residue Charge
pH

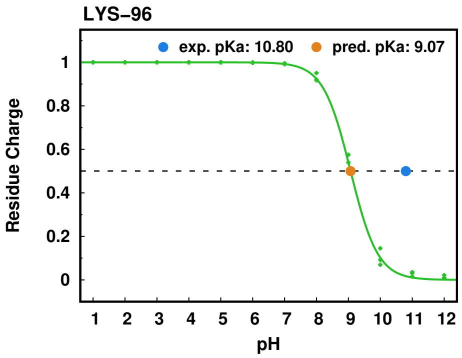
LYS–96
exp. pKa: 10.80
pred. pKa: 9.07
Residue Charge
pH

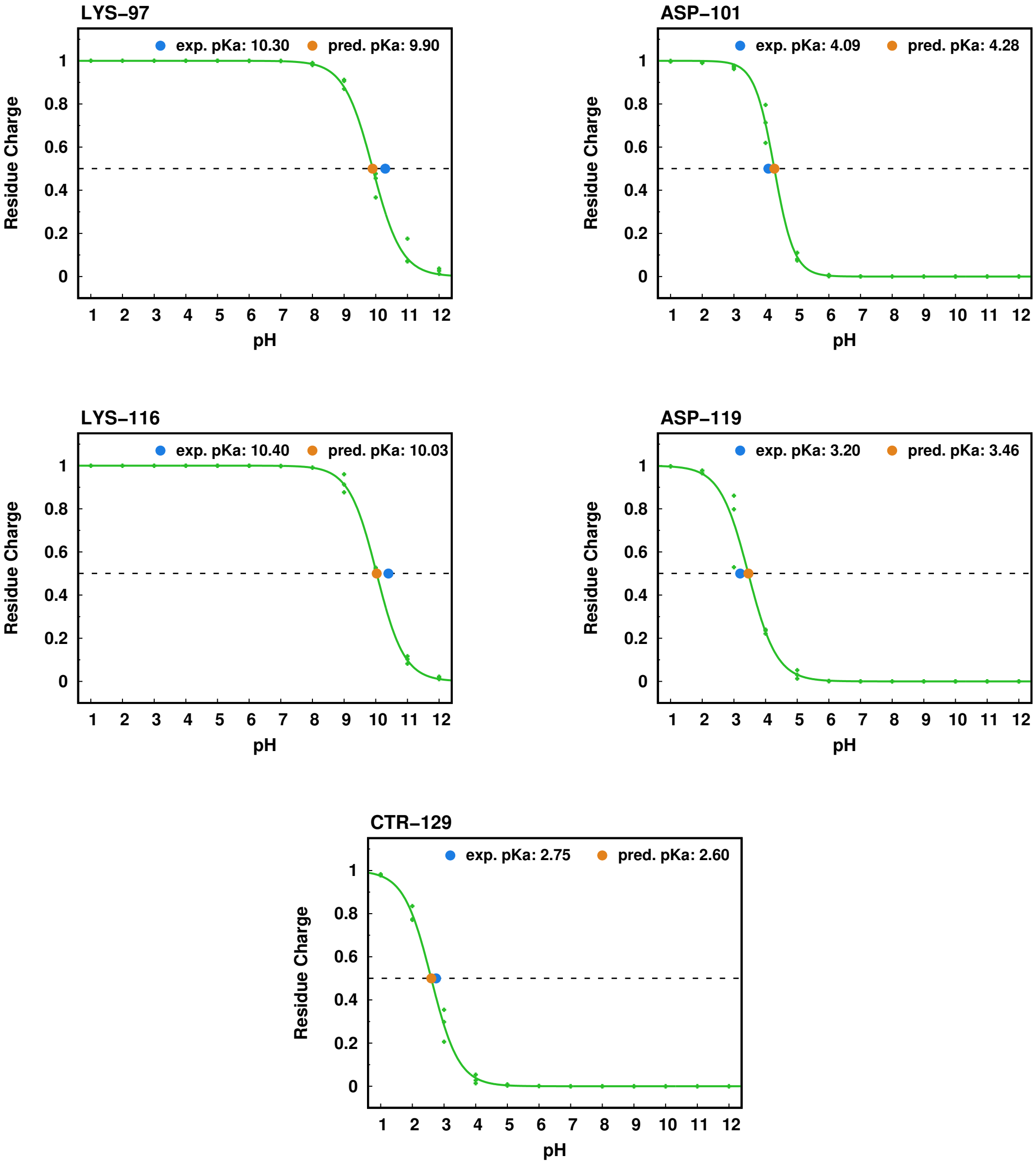

Figure S21: Titration curves for the titrating residues in the HEWL simulations, comparing experimental (blue dots, when available) and predicted (orange dots) pK_{a} values.

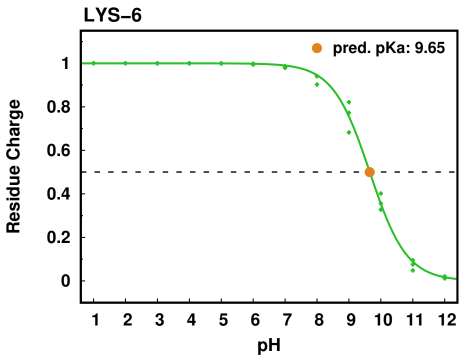
LYS−6
pred. pKa: 9.65
Residue Charge
pH

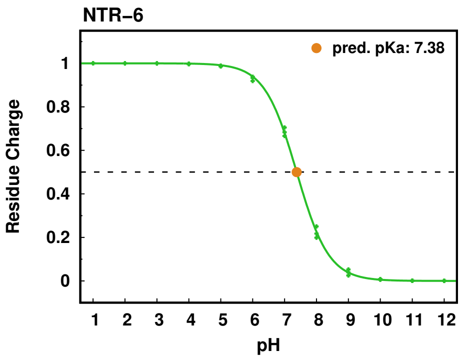
NTR−6
pred. pKa: 7.38
Residue Charge
pH

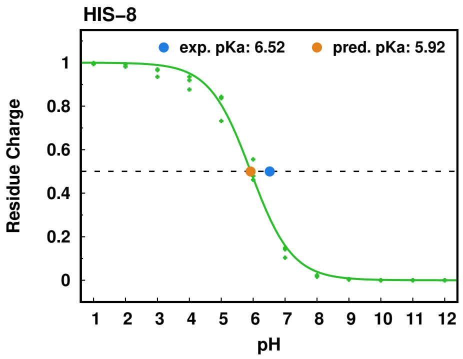
HIS−8
exp. pKa: 6.52
pred. pKa: 5.92
Residue Charge
pH

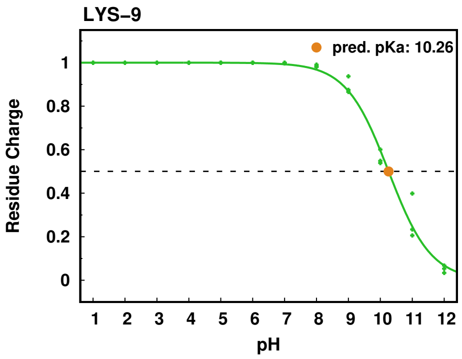
LYS−9
pred. pKa: 10.26
Residue Charge
pH

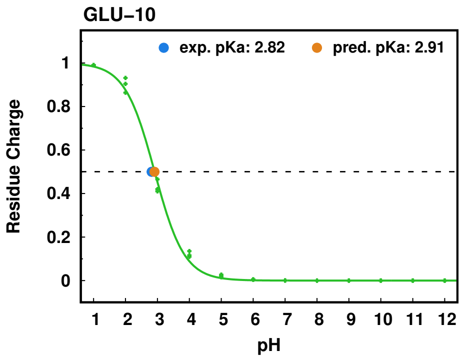
GLU−10
exp. pKa: 2.82
pred. pKa: 2.91
Residue Charge
pH

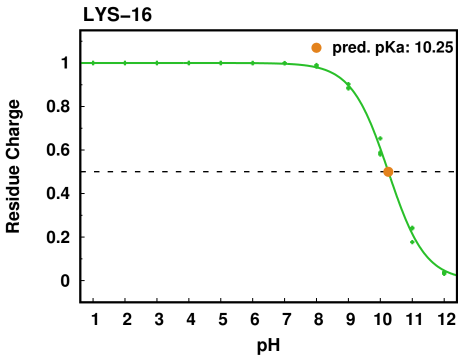
LYS−16
pred. pKa: 10.25
Residue Charge
pH

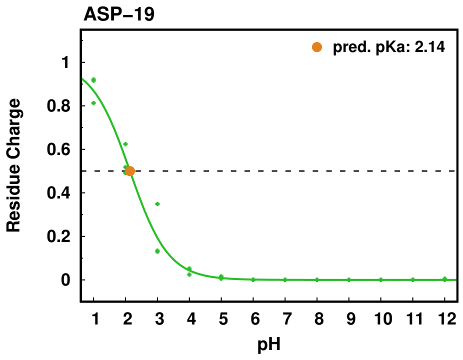
ASP−19
pred. pKa: 2.14
Residue Charge
pH

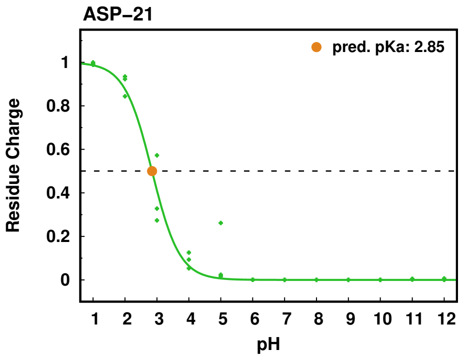
ASP−21
pred. pKa: 2.85
Residue Charge
pH

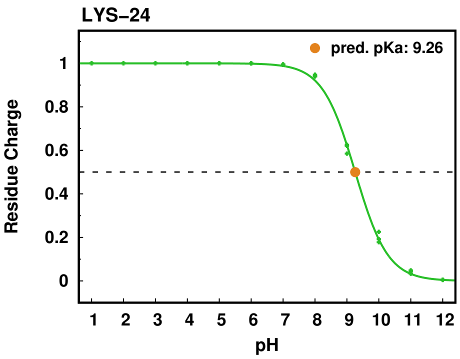
LYS–24
pred. pKa: 9.26
Residue Charge
pH

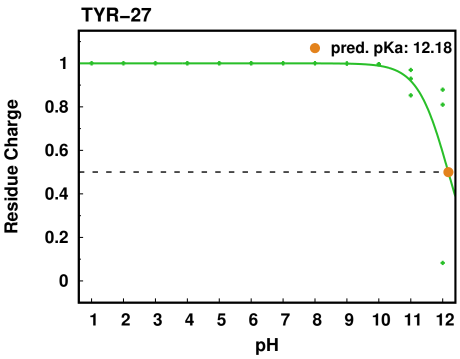
TYR–27
pred. pKa: 12.18
Residue Charge
pH

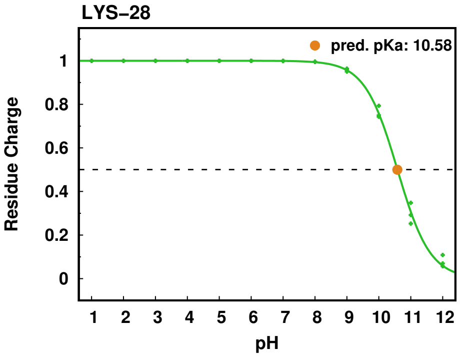
LYS–28
pred. pKa: 10.58
Residue Charge
pH

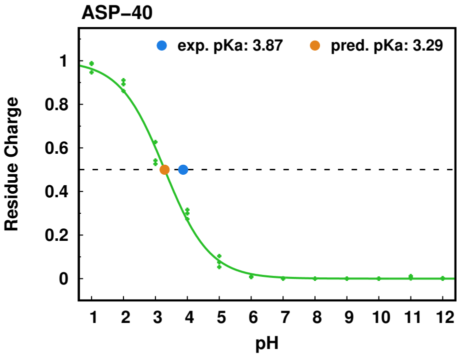
ASP–40
exp. pKa: 3.87
pred. pKa: 3.29
Residue Charge
pH

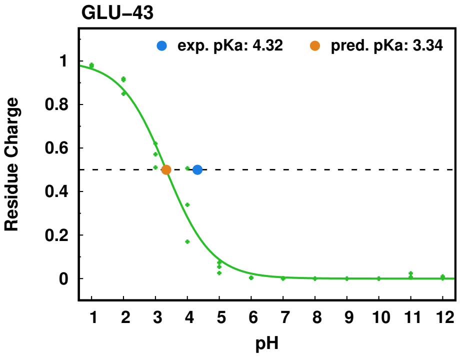
GLU–43
exp. pKa: 4.32
pred. pKa: 3.34
Residue Charge
pH

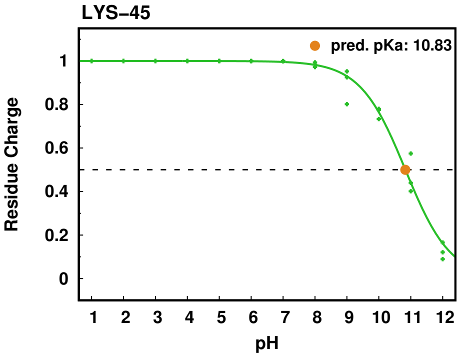
LYS–45
pred. pKa: 10.83
Residue Charge
pH

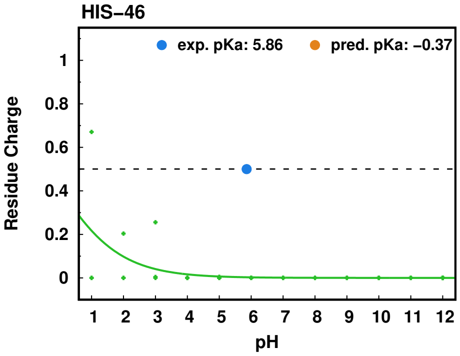
HIS–46
exp. pKa: 5.86
pred. pKa: –0.37
Residue Charge
pH

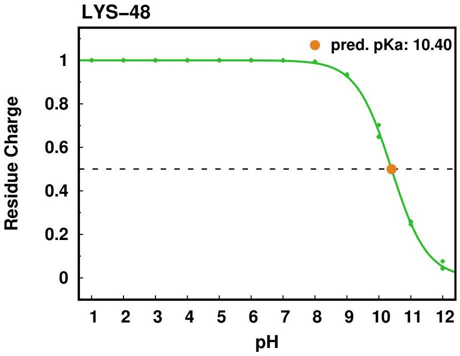
LYS–48
pred. pKa: 10.40
Residue Charge
pH

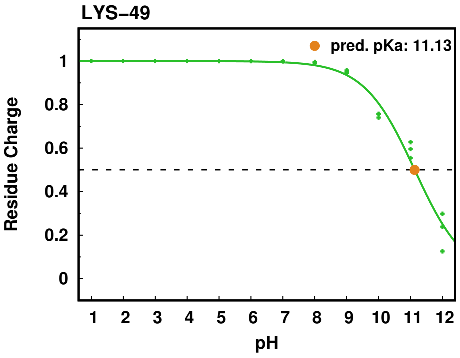
LYS–49
pred. pKa: 11.13
Residue Charge
pH

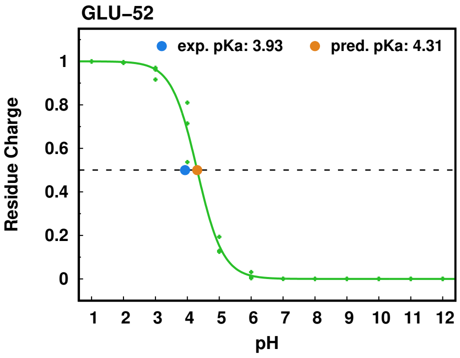
GLU–52
exp. pKa: 3.93
pred. pKa: 4.31
Residue Charge
pH

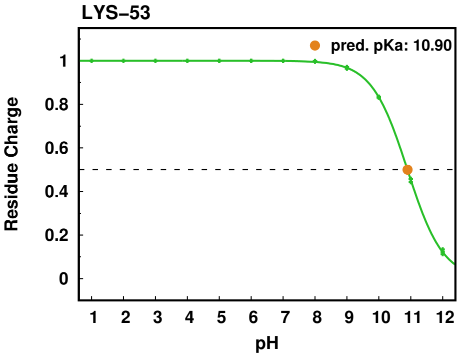
LYS–53
pred. pKa: 10.90
Residue Charge
pH

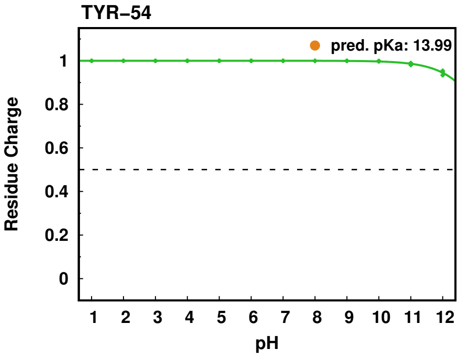
TYR–54
pred. pKa: 13.99
Residue Charge
pH

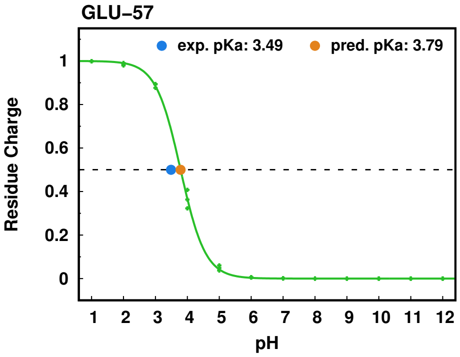
GLU–57
exp. pKa: 3.49
pred. pKa: 3.79
Residue Charge
pH

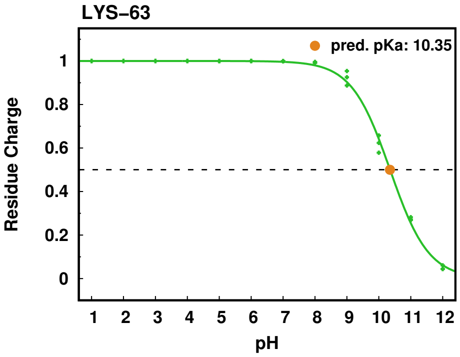
LYS–63
pred. pKa: 10.35
Residue Charge
pH

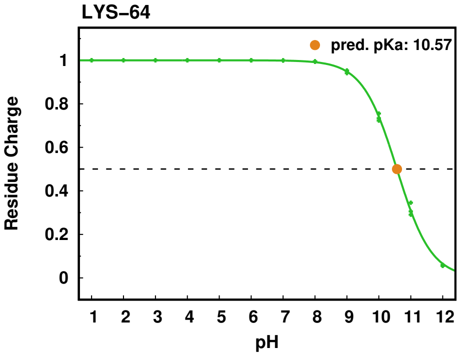
LYS–64
pred. pKa: 10.57
Residue Charge
pH

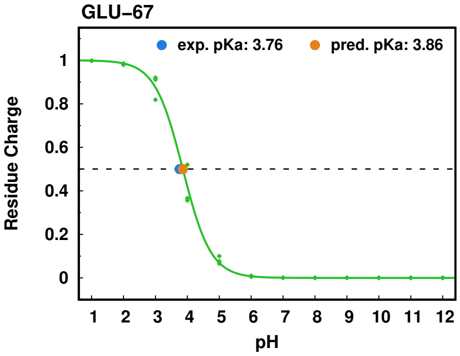
GLU–67
exp. pKa: 3.76
pred. pKa: 3.86
Residue Charge
pH

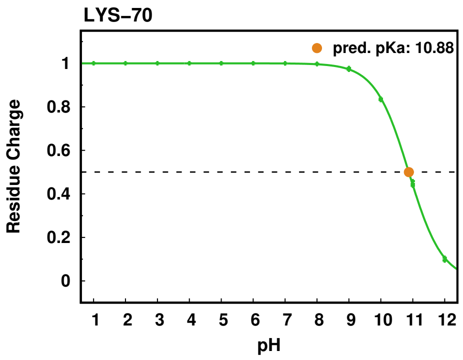
LYS–70
pred. pKa: 10.88
Residue Charge
pH

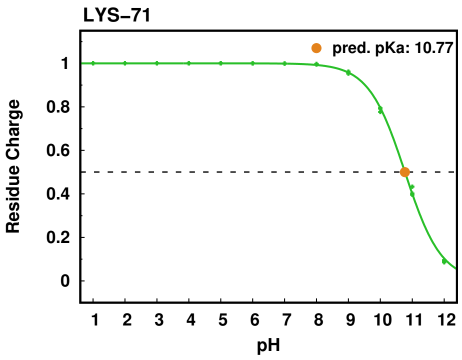
LYS–71
pred. pKa: 10.77
Residue Charge
pH

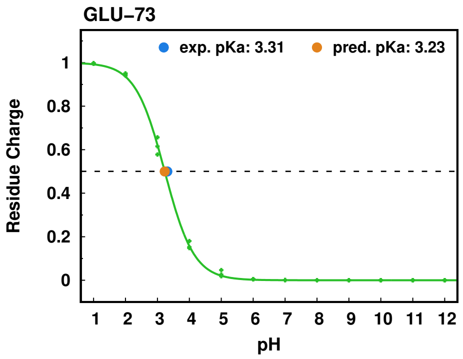
GLU–73
exp. pKa: 3.31
pred. pKa: 3.23
Residue Charge
pH

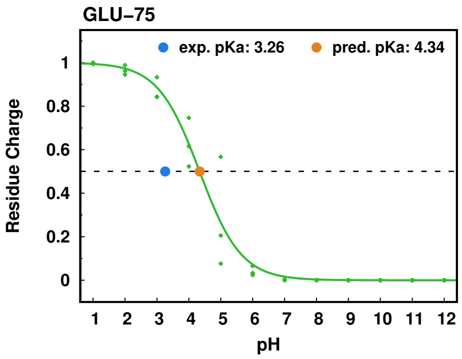
GLU–75
exp. pKa: 3.26
pred. pKa: 4.34
Residue Charge
pH

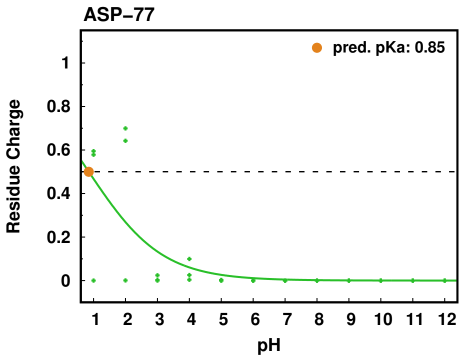
ASP–77
pred. pKa: 0.85
Residue Charge
pH

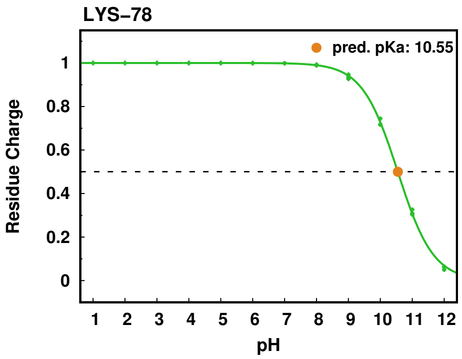
LYS–78
pred. pKa: 10.55
Residue Charge
pH

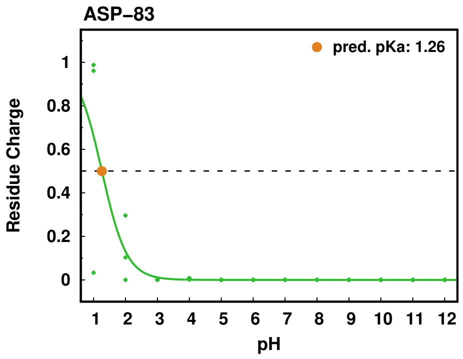
ASP–83
pred. pKa: 1.26
Residue Charge
pH

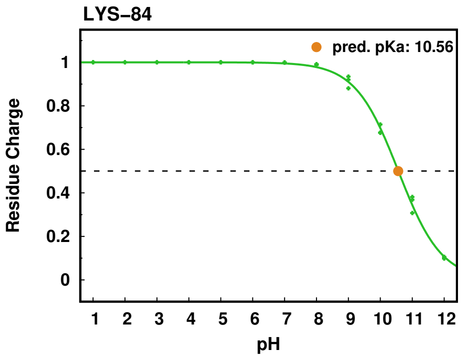
LYS–84
pred. pKa: 10.56
Residue Charge
pH

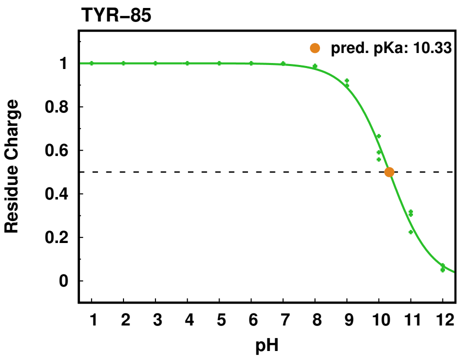
TYR–85
pred. pKa: 10.33
Residue Charge
pH

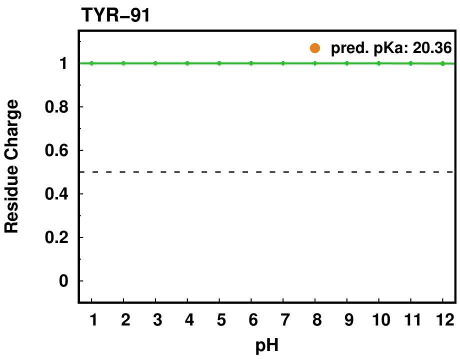
TYR–91
pred. pKa: 20.36
Residue Charge
pH

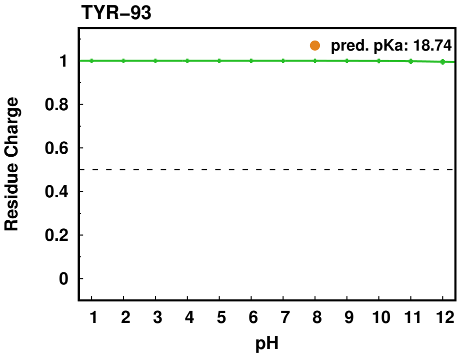
TYR–93
pred. pKa: 18.74
Residue Charge
pH

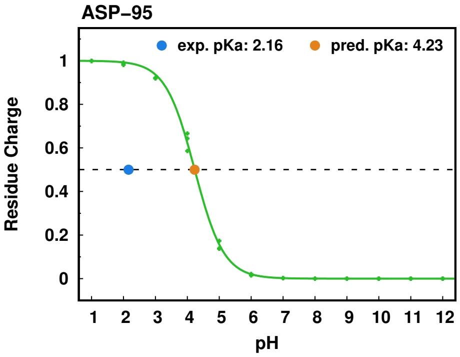
ASP–95
exp. pKa: 2.16
pred. pKa: 4.23
Residue Charge
pH

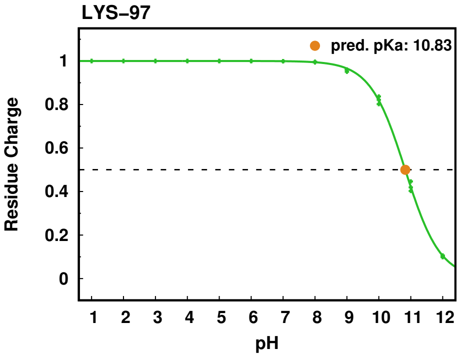
LYS–97
pred. pKa: 10.83
Residue Charge
pH

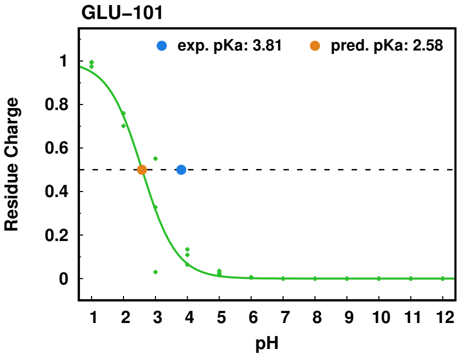
GLU–101
exp. pKa: 3.81
pred. pKa: 2.58
Residue Charge
pH

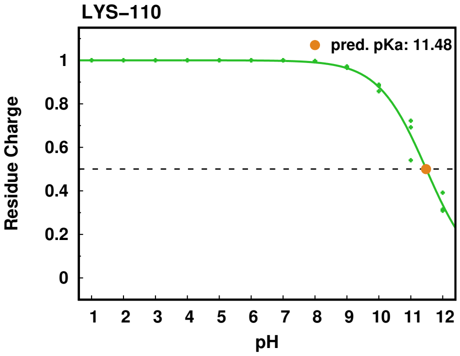
LYS–110
pred. pKa: 11.48
Residue Charge
pH

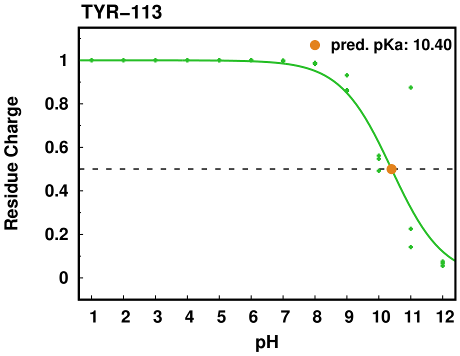
TYR–113
pred. pKa: 10.40
Residue Charge
pH

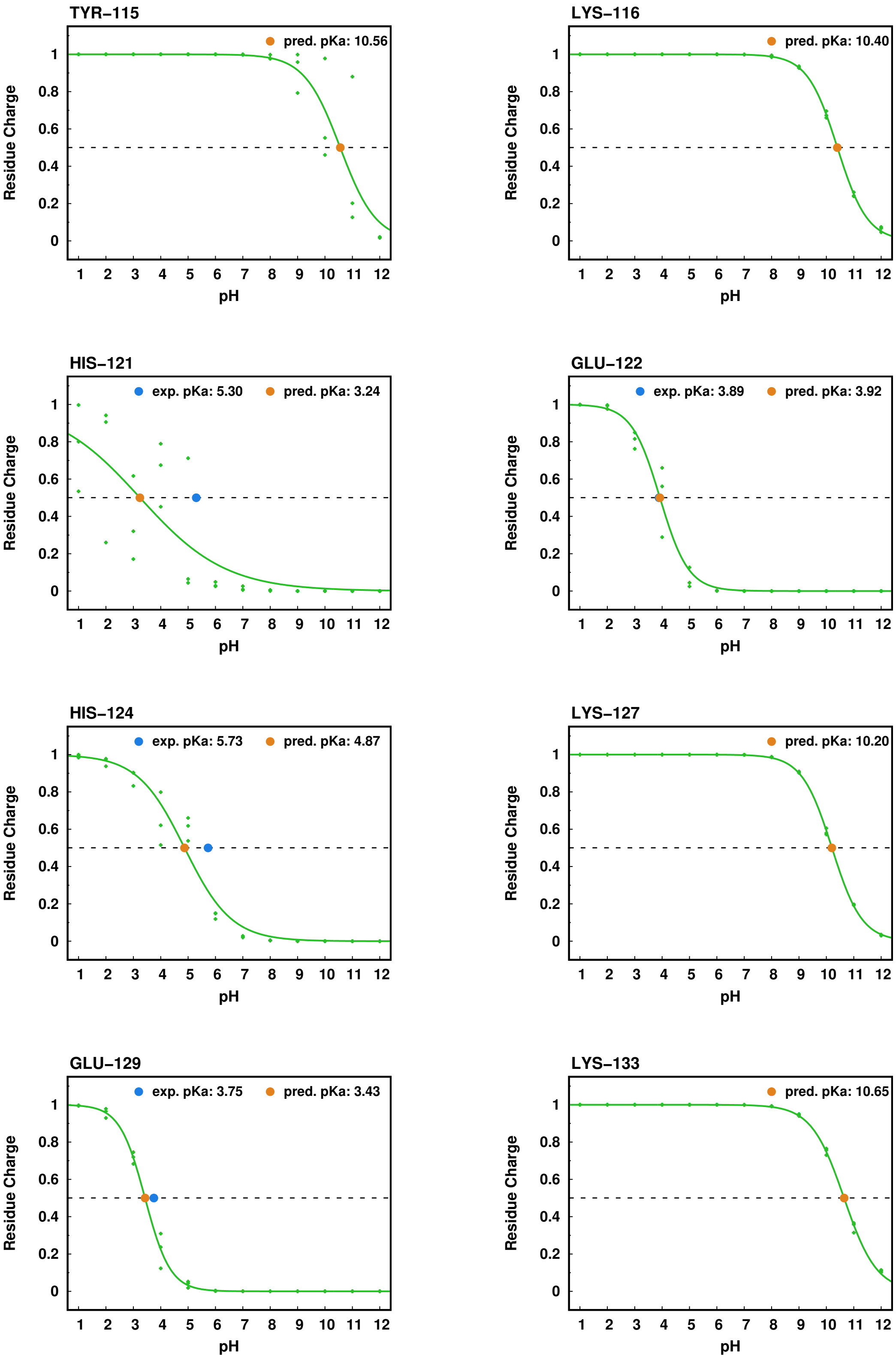
TYR–115
pred. pKa: 10.56
LYS–116
pred. pKa: 10.40
HIS–121
exp. pKa: 5.30
pred. pKa: 3.24
GLU–122
exp. pKa: 3.89
pred. pKa: 3.92
HIS–124
exp. pKa: 5.73
pred. pKa: 4.87
LYS–127
pred. pKa: 10.20
GLU–129
exp. pKa: 3.75
pred. pKa: 3.43
LYS–133
pred. pKa: 10.65
Residue Charge
pH

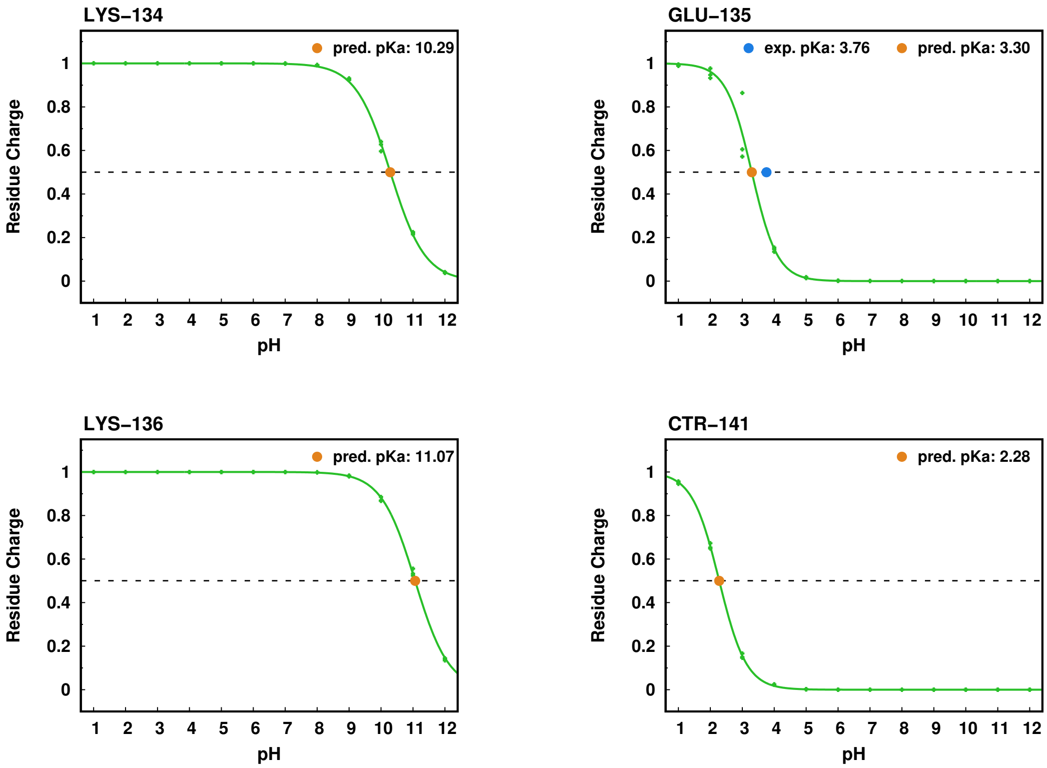

Figure S22: Titration curves for the titrating residues in the SNase simulations, comparing experimental (blue dots, when available) and predicted (orange dots) pK_a values.

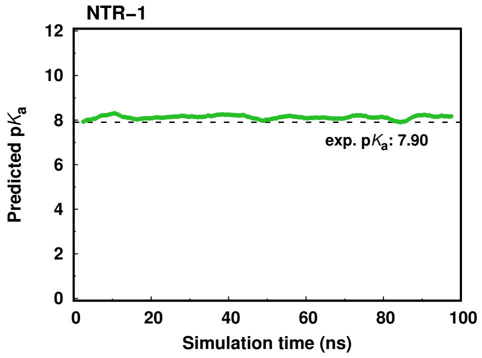
NTR−1
Predicted pK_a
exp. pK_a: 7.90
Simulation time (ns)

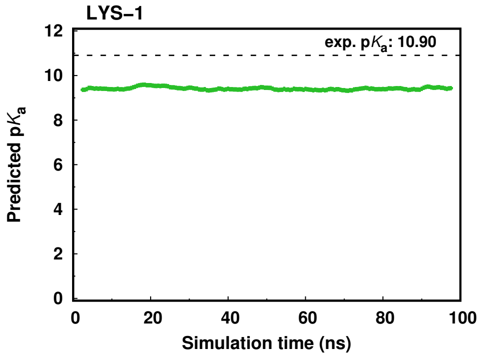
LYS−1
Predicted pK_a
exp. pK_a: 10.90
Simulation time (ns)

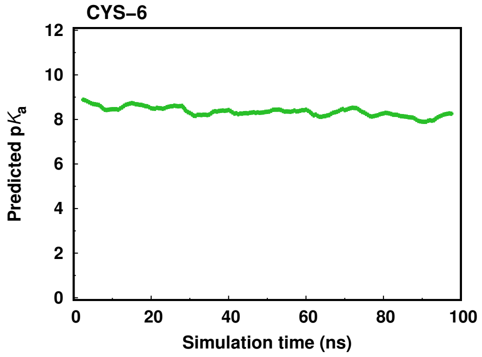
CYS−6
Predicted pK_a
Simulation time (ns)

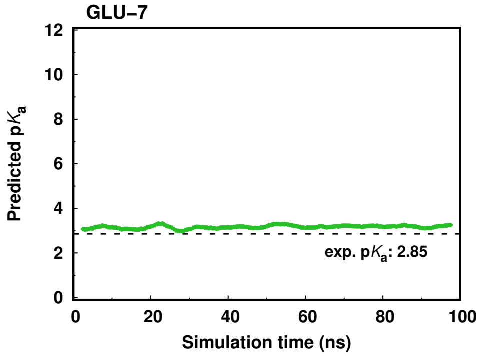
GLU−7
Predicted pK_a
exp. pK_a: 2.85
Simulation time (ns)

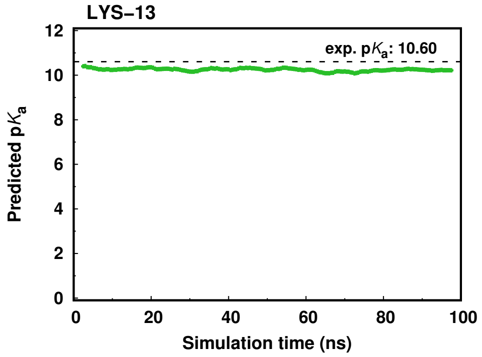
LYS−13
Predicted pK_a
exp. pK_a: 10.60
Simulation time (ns)

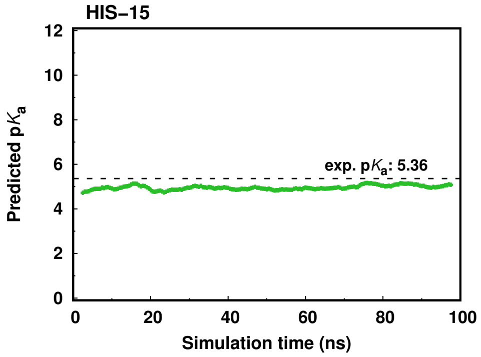
HIS−15
Predicted pK_a
exp. pK_a: 5.36
Simulation time (ns)

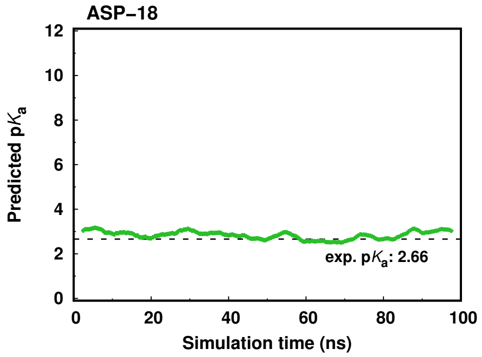
ASP−18
Predicted pK_a
exp. pK_a: 2.66
Simulation time (ns)

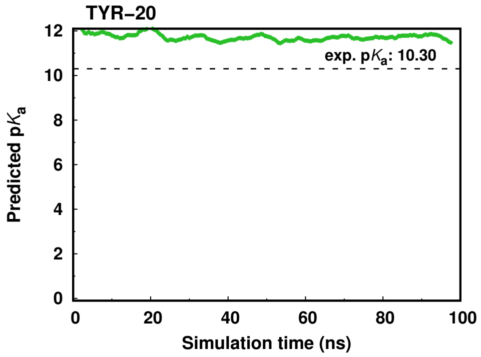
TYR−20
Predicted pK_a
exp. pK_a: 10.30
Simulation time (ns)

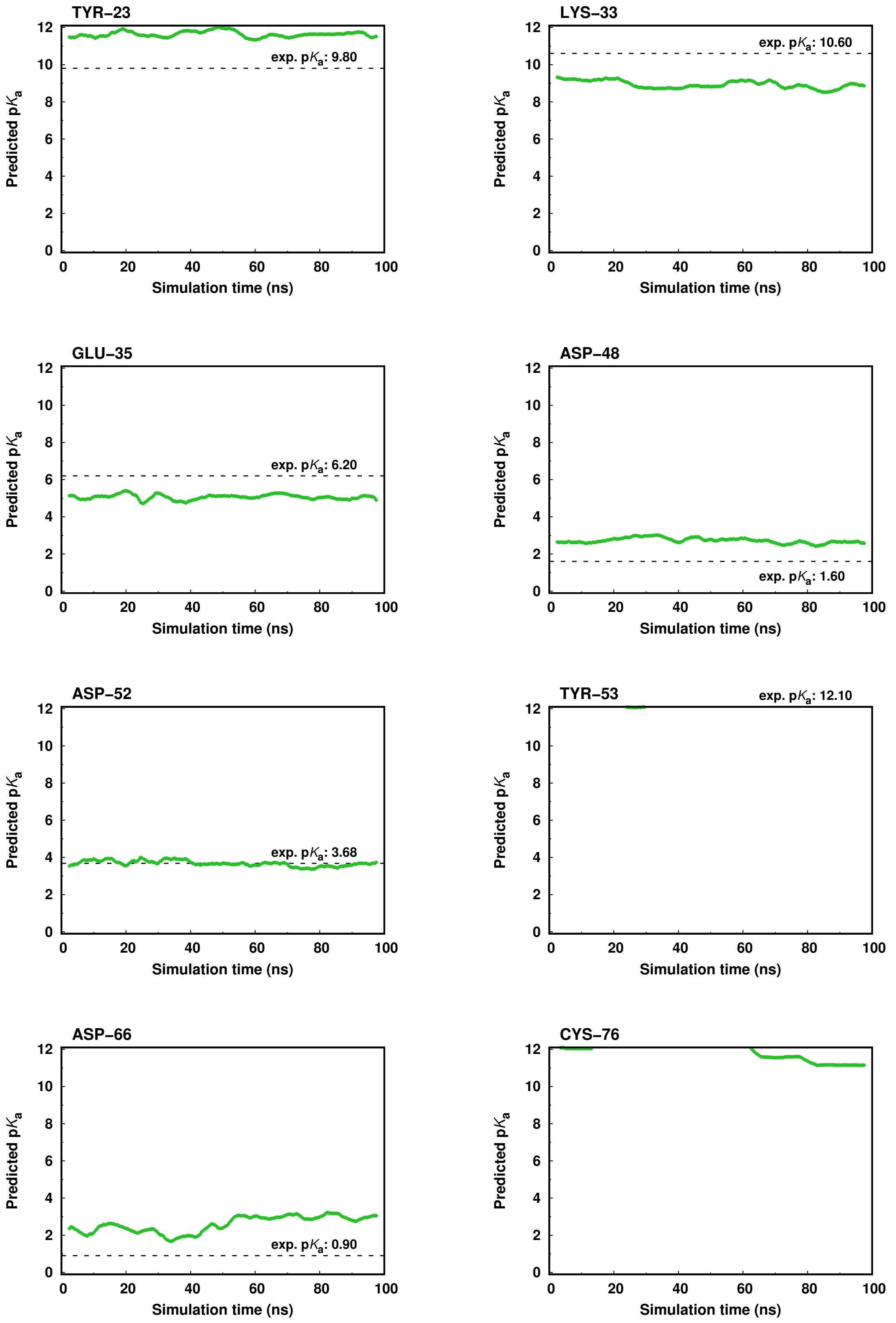

TYR–23
exp. pKa: 9.80
LYS–33
exp. pKa: 10.60
GLU–35
exp. pKa: 6.20
ASP–48
exp. pKa: 1.60
ASP–52
exp. pKa: 3.68
TYR–53
exp. pKa: 12.10
ASP–66
exp. pKa: 0.90
CYS–76
Predicted pKa
Simulation time (ns)
0
2
4
6
8
10
12
20
40
60
80
100

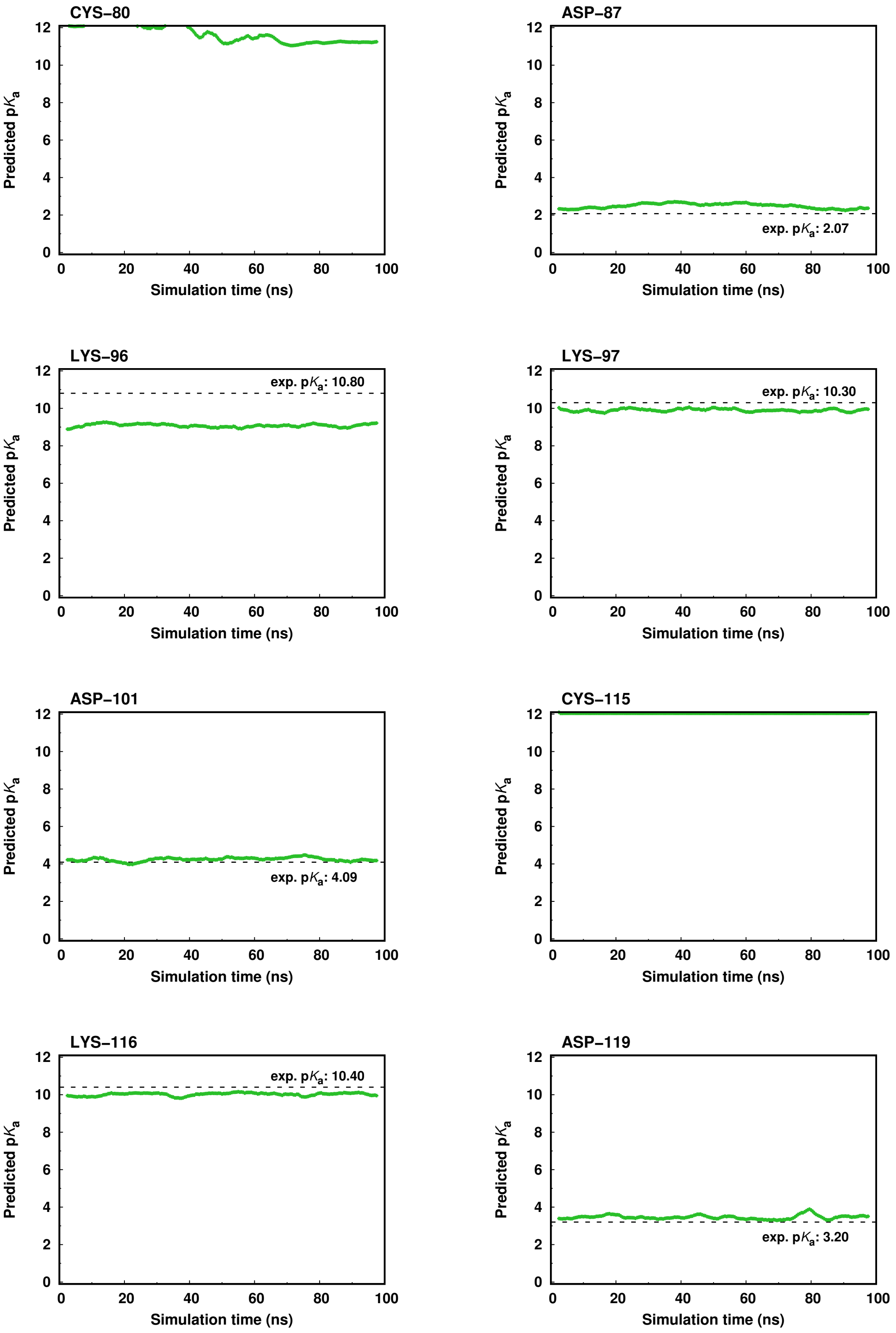
CYS−80
ASP−87
exp. pK_a: 2.07
LYS−96
exp. pK_a: 10.80
LYS−97
exp. pK_a: 10.30
ASP−101
exp. pK_a: 4.09
CYS−115
LYS−116
exp. pK_a: 10.40
ASP−119
exp. pK_a: 3.20
Predicted pK_a
Simulation time (ns)

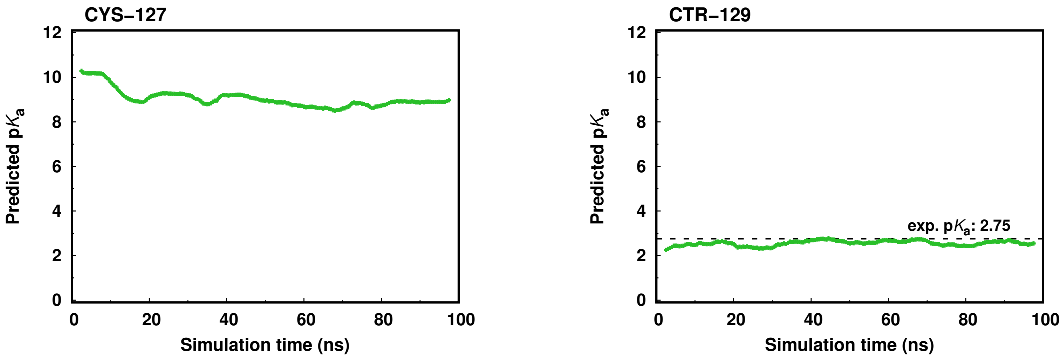

Figure S23: pK_a values calculated in 5 ns windows over time, for all HEWL titratable residues. When available, experimental pK_a values are shown as a dotted line. Since the predicted pK_a values for Cys-30, Cys-64, and Cys-94 were >12, the plots for these residues were omitted.

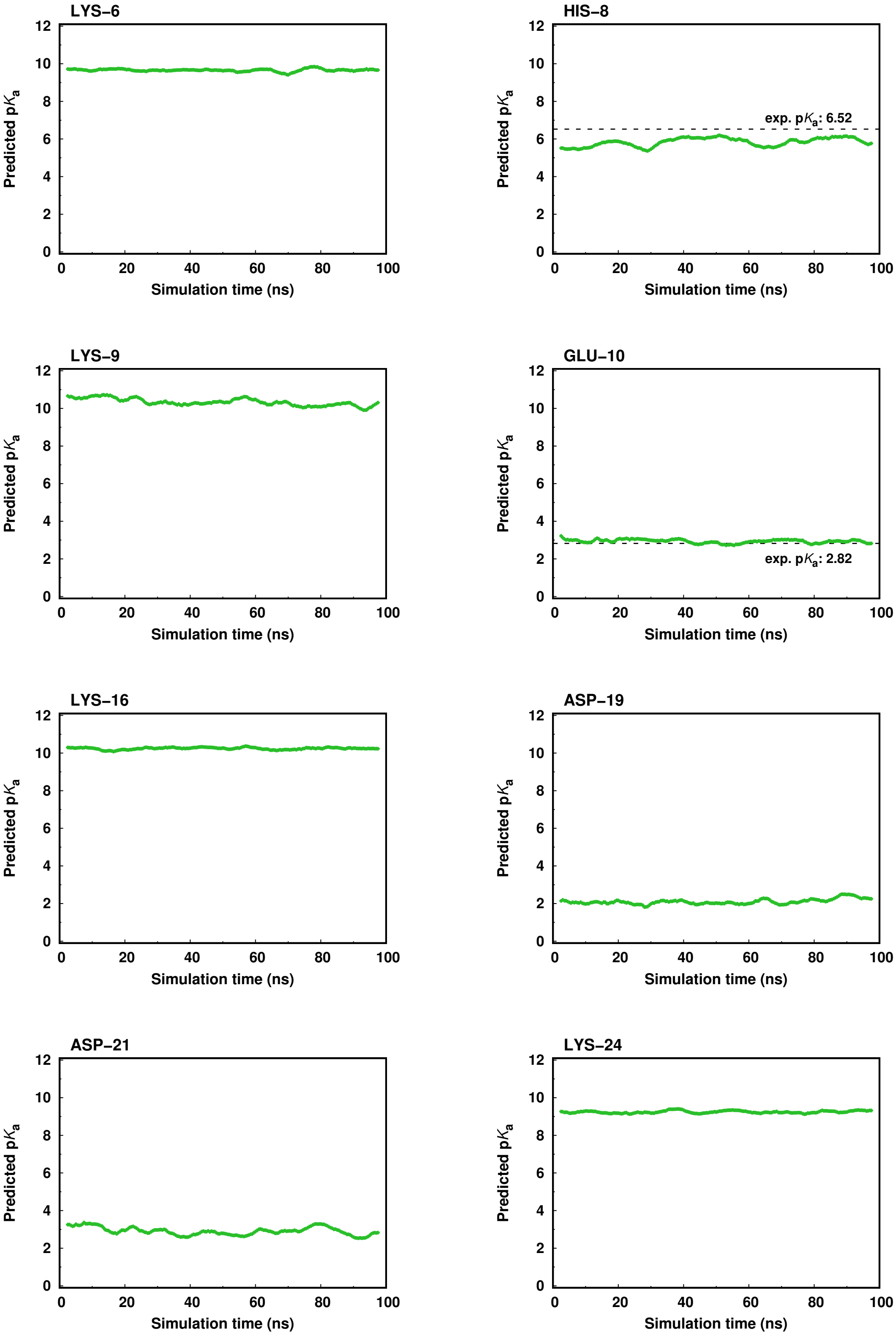
LYS–6
HIS–8
exp. pK_a: 6.52
LYS–9
GLU–10
exp. pK_a: 2.82
LYS–16
ASP–19
ASP–21
LYS–24
Predicted pK_a
Simulation time (ns)
0
2
4
6
8
10
12
20
40
60
80
100

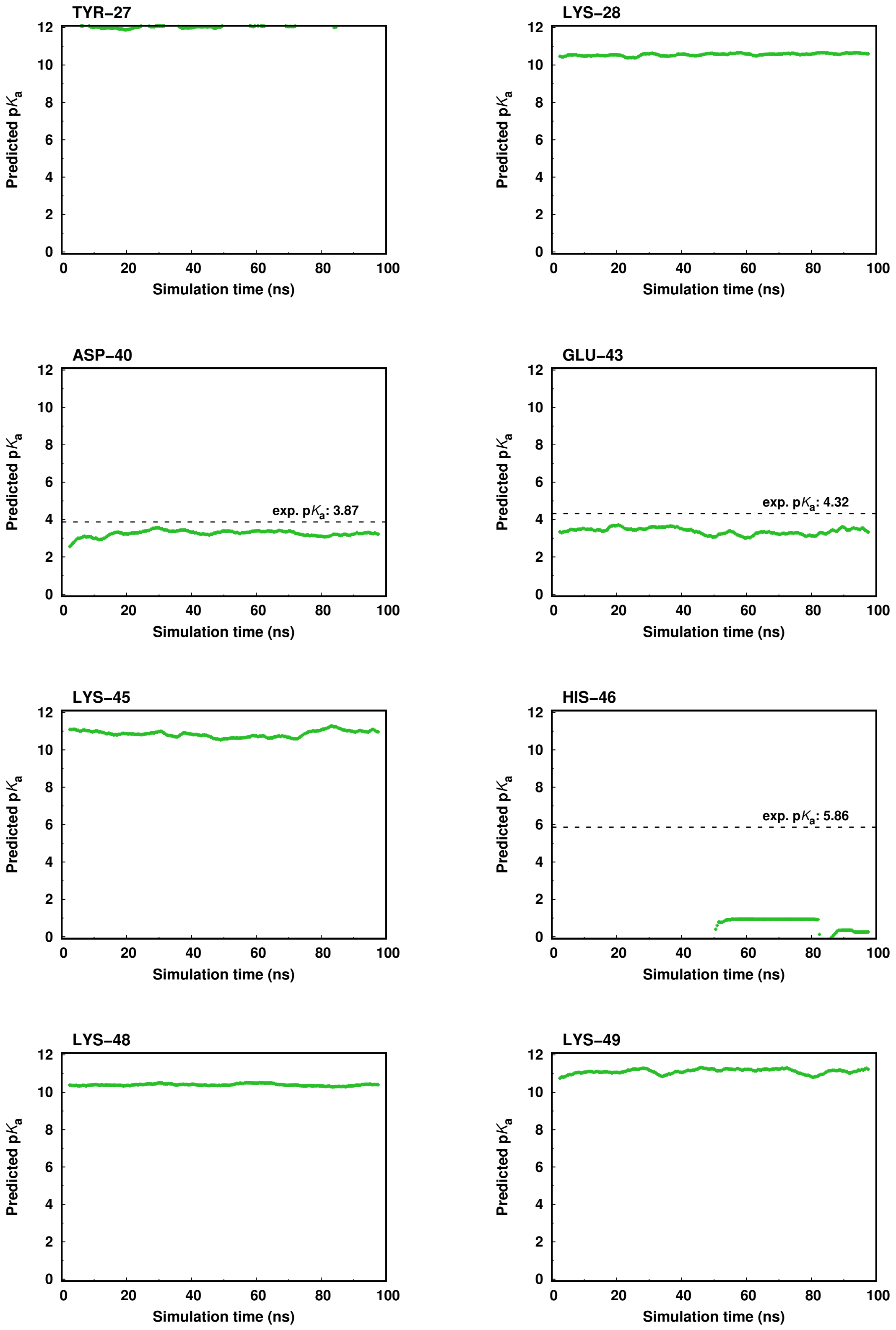
TYR–27
LYS–28
ASP–40
exp. pK_a: 3.87
GLU–43
exp. pK_a: 4.32
LYS–45
HIS–46
exp. pK_a: 5.86
LYS–48
LYS–49
Predicted pK_a
Simulation time (ns)
0
2
4
6
8
10
12
20
40
60
80
100

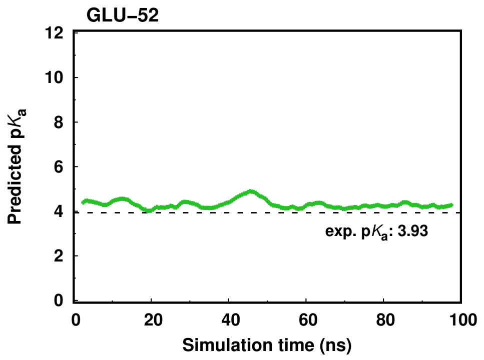
GLU–52
Predicted pK_a
Simulation time (ns)
exp. pK_a: 3.93

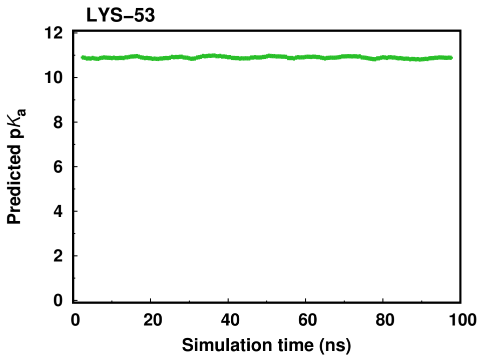
LYS–53
Predicted pK_a
Simulation time (ns)

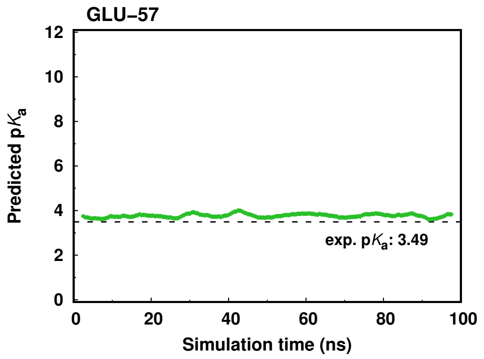
GLU–57
Predicted pK_a
Simulation time (ns)
exp. pK_a: 3.49

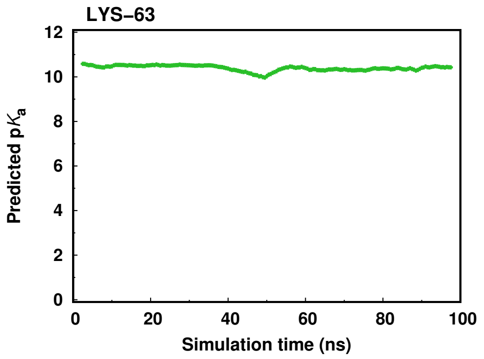
LYS–63
Predicted pK_a
Simulation time (ns)

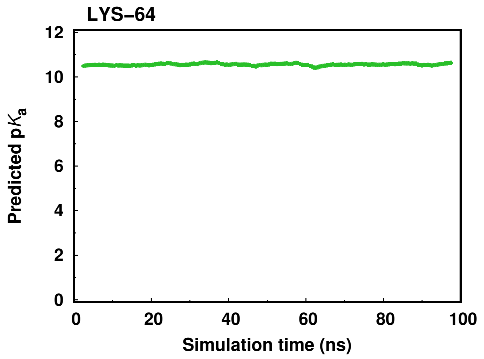
LYS–64
Predicted pK_a
Simulation time (ns)

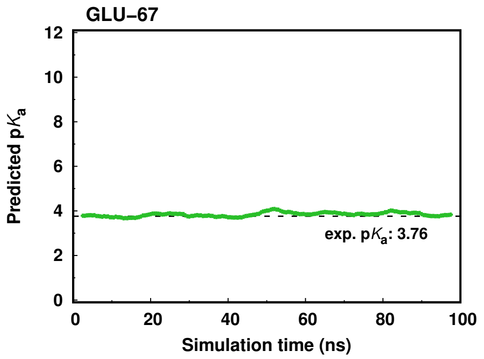
GLU–67
Predicted pK_a
Simulation time (ns)
exp. pK_a: 3.76

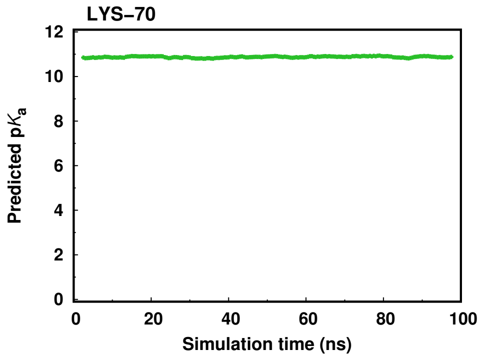
LYS–70
Predicted pK_a
Simulation time (ns)

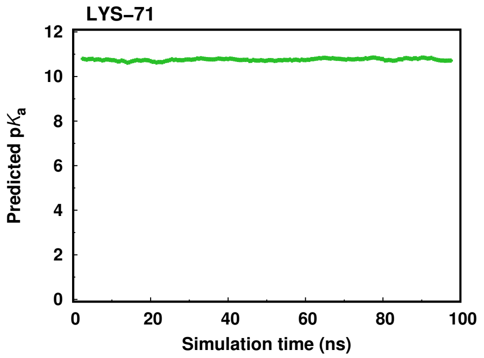
LYS–71
Predicted pK_a
Simulation time (ns)

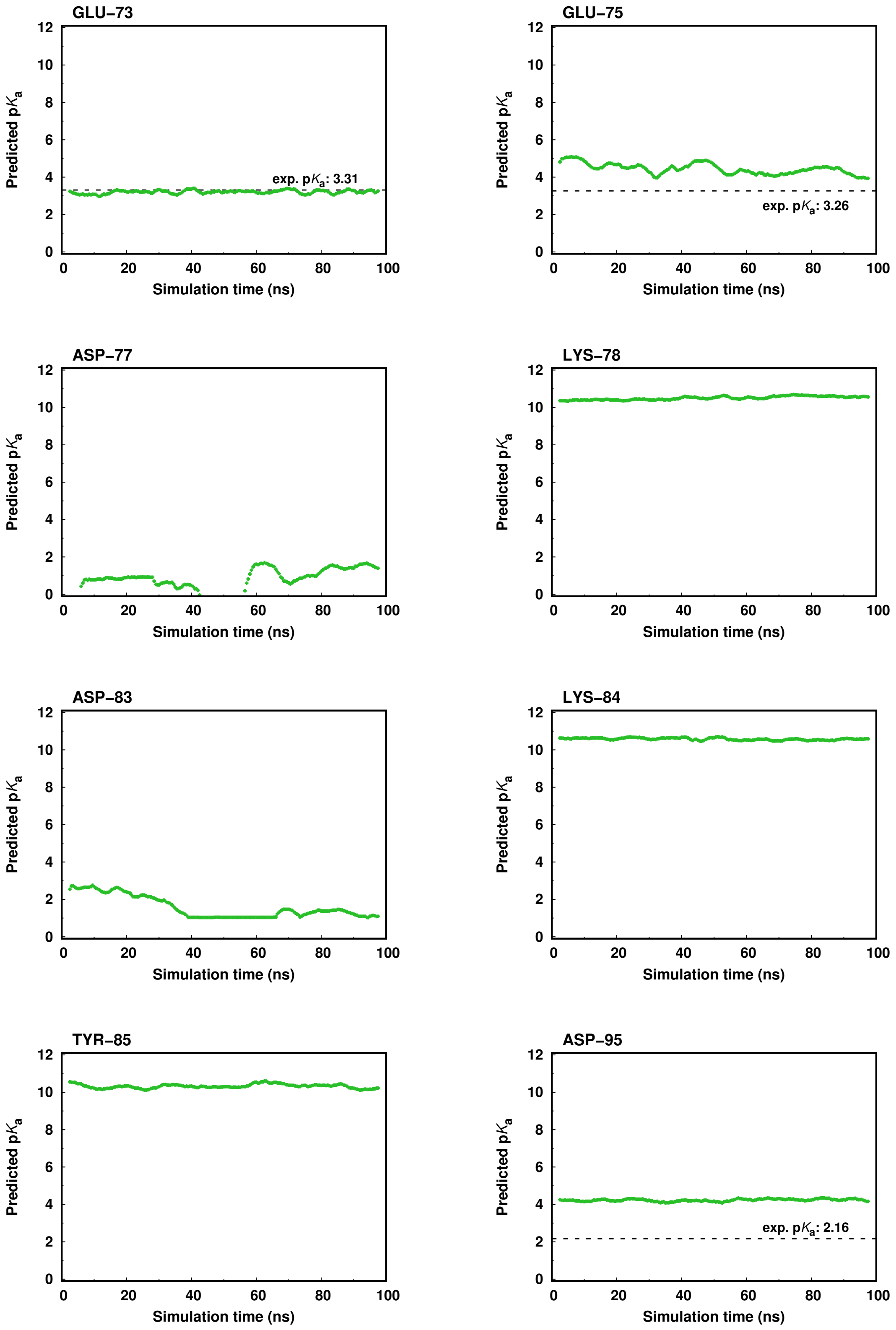
GLU–73
exp. pK_a: 3.31
GLU–75
exp. pK_a: 3.26
ASP–77
LYS–78
ASP–83
LYS–84
TYR–85
ASP–95
exp. pK_a: 2.16
Predicted pK_a
Simulation time (ns)

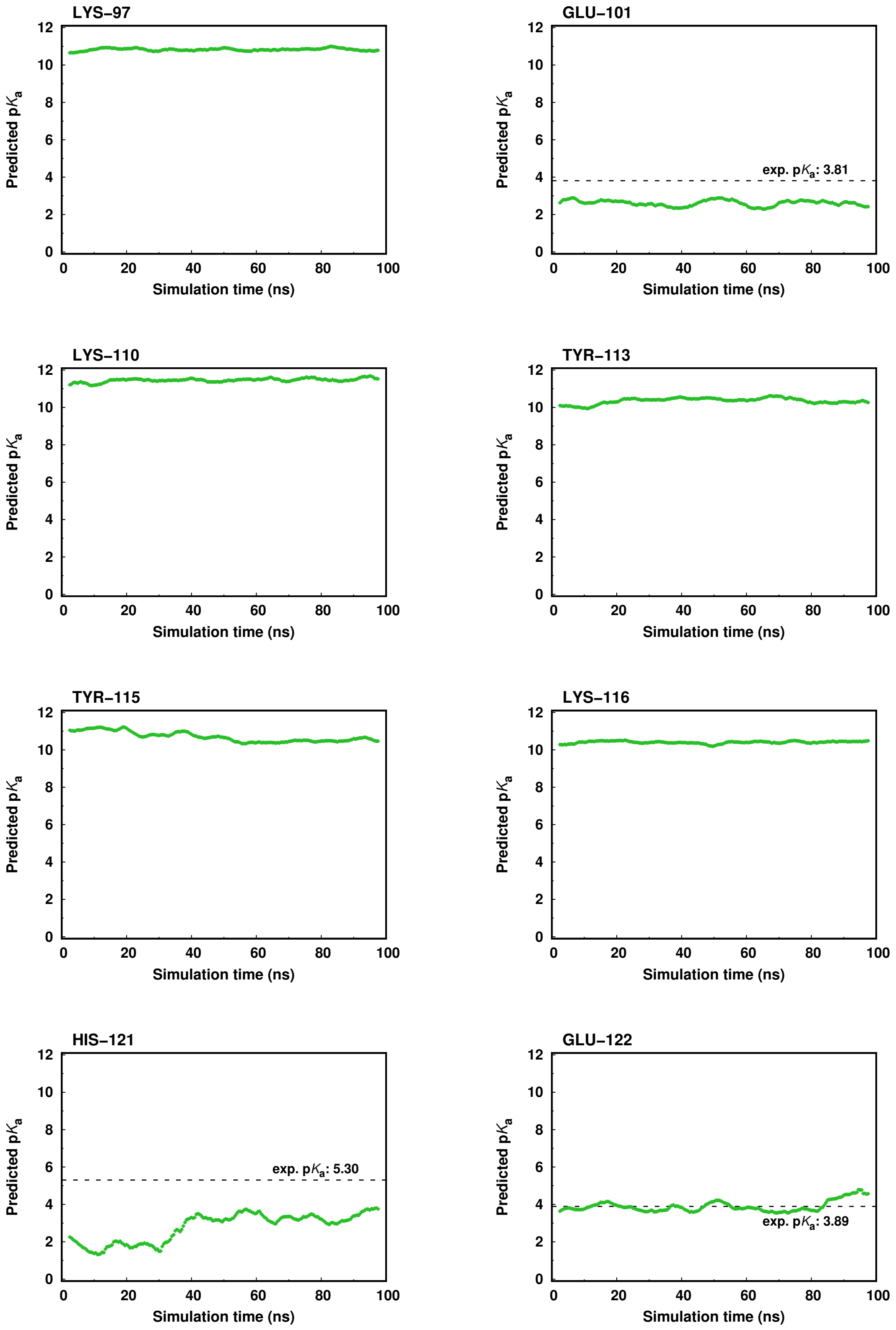
LYS−97
GLU−101
exp. pK_a: 3.81
LYS−110
TYR−113
TYR−115
LYS−116
HIS−121
exp. pK_a: 5.30
GLU−122
exp. pK_a: 3.89
Predicted pK_a
Simulation time (ns)
0
2
4
6
8
10
12
0
20
40
60
80
100

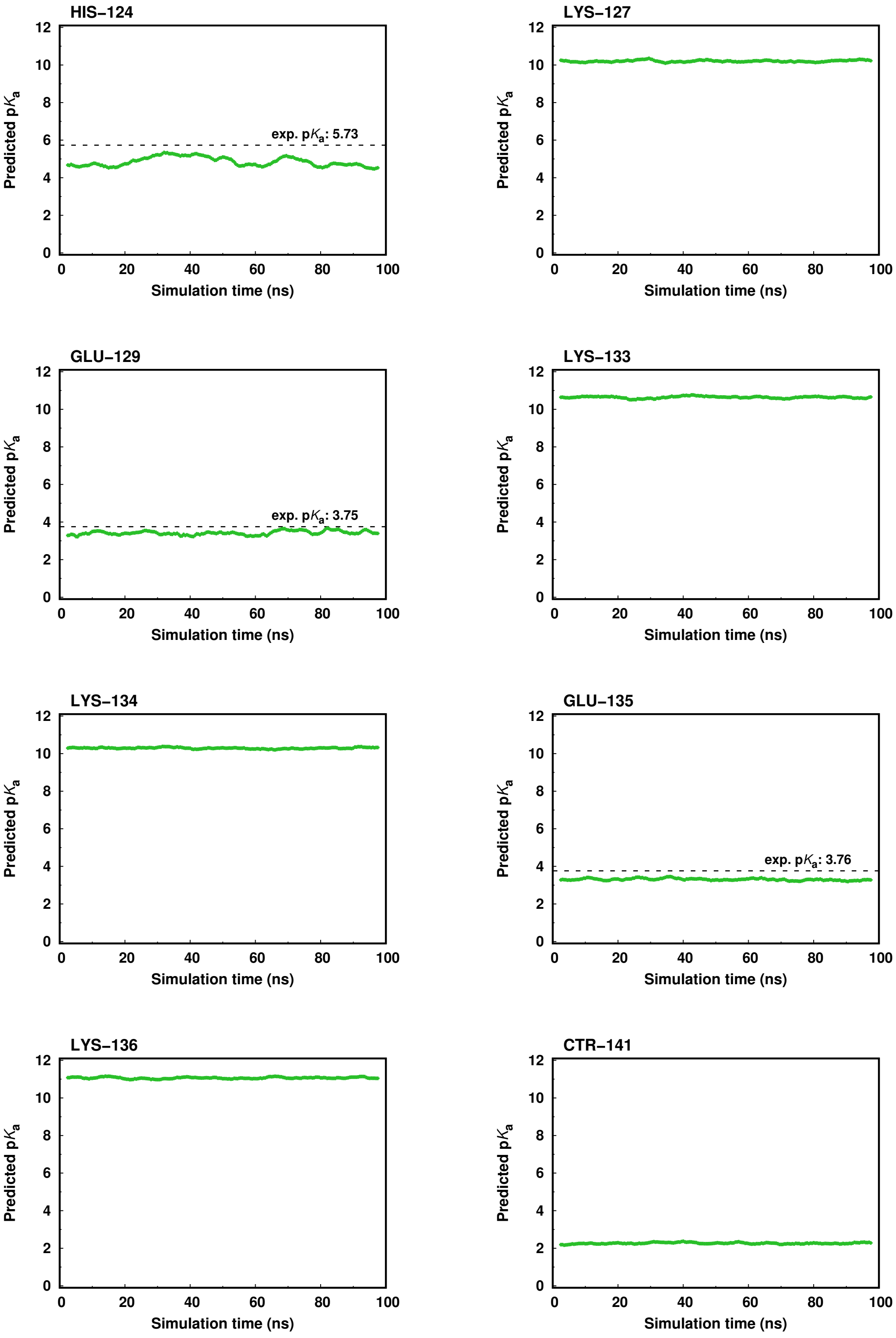

Figure S24: pK_a values calculated in 5 ns windows over time, for all SNase titratable residues. When available, experimental pK_a values are shown as a dotted line. Since the predicted pK_a values for Tyr-54, Tyr-91, and Tyr-93 were >12, the plots for these residues were omitted.

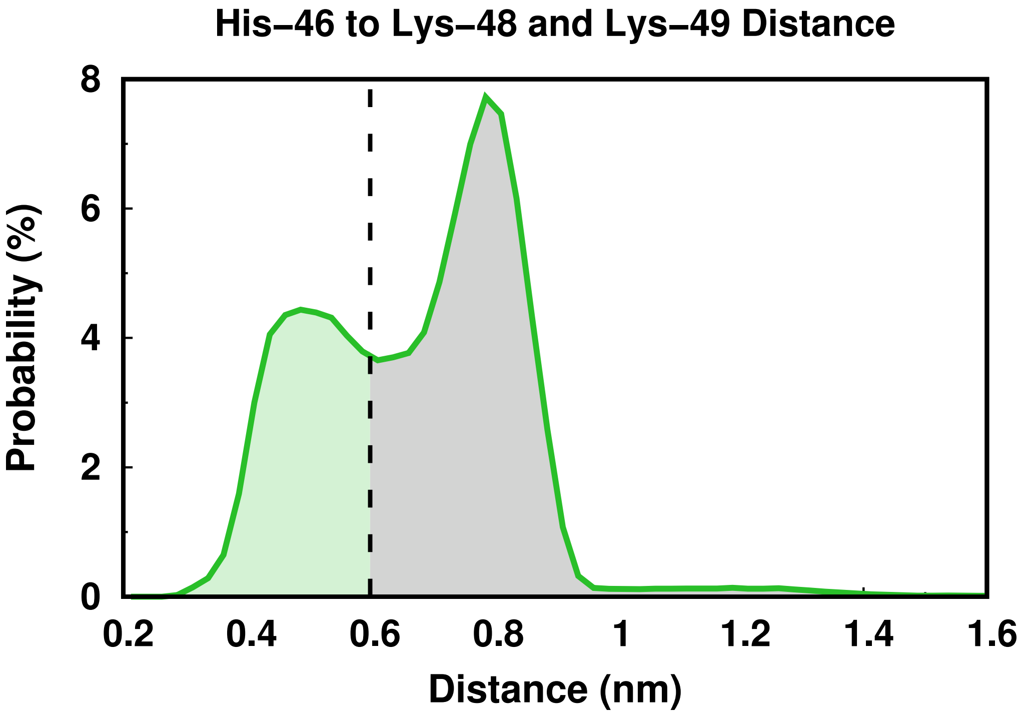

Figure S25: Distance histogram between His-46 and both Lys-48 and Lys-49, for all simulated pH values. The green area shows where His-46 and Lys interact (side chain distance < 0.6 nm), while the grey area indicates where they do not.

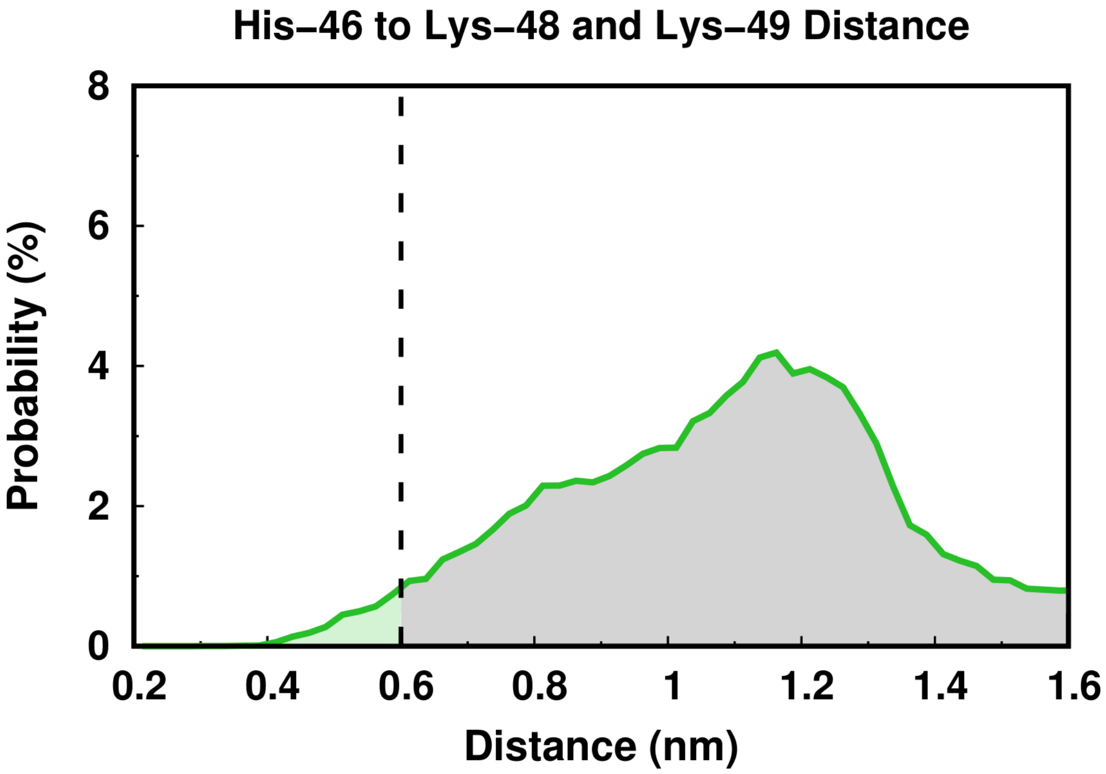

Figure S26: Distance histogram between His-46 and both Lys-48 and Lys-49, for the second set of simulations at pH 2, starting from a conformation where His-46 was not interacting with any Glu residue. The green area shows where His-46 and Glu interact (side chain distance < 0.6 nm), while the grey area indicates where they do not.

References

(S1) Thurlkill, R. L.; Grimsley, G. R.; Scholtz, J. M.; Pace, C. N. pK values of the ionizable groups of proteins. *Protein Sci.* **2006**, *15*, 1214–1218.

(S2) Grimsley, G. R.; Scholtz, J. M.; Pace, C. N. A summary of the measured pK values of the ionizable groups in folded proteins. *Protein Sci.* **2009**, *18*, 247–251.

(S3) Machuqueiro, M.; Baptista, A. M. Is the prediction of pK_a values by constant-pH molecular dynamics being hindered by inherited problems? *Proteins Struct. Funct. Bioinf.* **2011**, *79*, 3437–3447.

(S4) Tanford, C.; Roxby, R. Interpretation of protein titration curves. Application to lysozyme. *Biochemistry* **1972**, *11*, 2192–2198.

(S5) Visser, A.; van Engelen, J.; Visser, N.; van Hoek, A.; Hilhorst, R.; Freedman, R. Fluorescence dynamics of staphylococcal nuclease in aqueous solution and reversed micelles. *Biochim. Biophys. Acta (BBA) Protein Struct. Mol. Enzymol.* **1994**, *1204*, 225–234.

(S6) Sequeira, J. G.; Rodrigues, F. E.; Silva, T. G.; Reis, P. B.; Machuqueiro, M. Extending the stochastic titration CpHMD to CHARMM36m. *J. Phys. Chem. B* **2022**, *126*, 7870–7882.

(S7) Pahari, S.; Sun, L.; Alexov, E. PKAD: a database of experimentally measured pK_a values of ionizable groups in proteins. *Database* **2019**, *2019*.

(S8) Kullback, S.; Leibler, R. A. On information and sufficiency. *Ann. Math. Stat.* **1951**, *22*, 79–86.
